# Effect of a Telephone Health Coaching Intervention on Hypertension Control in Young Adults: The MyHEART Randomized Clinical Trial

**DOI:** 10.1001/jamanetworkopen.2022.55618

**Published:** 2023-02-03

**Authors:** Kara K. Hoppe, Maureen Smith, Jennifer Birstler, KyungMann Kim, Lisa Sullivan-Vedder, Jamie N. LaMantia, Megan R. Knutson Sinaise, Matthew Swenson, Jennifer Fink, Ryan Haggart, Patrick McBride, Diane R. Lauver, Heather M. Johnson

**Affiliations:** 1 Department of Obstetrics & Gynecology, School of Medicine and Public Health, University of Wisconsin–Madison, Madison; 2 Departments of Population Health Sciences and Family Medicine & Community Health, School of Medicine and Public Health, University of Wisconsin–Madison, Madison; 3 Department of Biostatistics and Medical Informatics, University of Wisconsin–Madison, Madison; 4 Aurora Health Care Department of Family Medicine, Family Care Center–Milwaukee, Milwaukee, Wisconsin; 5 Department of Medicine, University of Wisconsin–Madison, Madison; 6 Medical College of Wisconsin, Milwaukee; 7 Department of Health Informatics and Administration, University of Wisconsin–Milwaukee, Milwaukee; 8 Department of Urology, University of Minnesota, Minneapolis; 9 School of Nursing, University of Wisconsin–Madison, Madison; 10 Christine E. Lynn Women’s Health & Wellness Institute, Baptist Health South Florida, Boca Raton; 11 Charles E. Schmidt College of Medicine, Florida Atlantic University, Boca Raton

## Abstract

**Importance:**

Uncontrolled hypertension (ie, a 24-hour ambulatory systolic blood pressure of ≥130 mm Hg and diastolic blood pressure of ≥80 mm Hg or clinic systolic blood pressure of ≥140 mm Hg and diastolic blood pressure of ≥90 mm Hg) in young adults is a US public health burden.

**Objective:**

To evaluate the effect of a telephone coaching and blood pressure self-monitoring intervention compared with usual care on changes in systolic and diastolic blood pressures and behaviors at 6 and 12 months.

**Design, Setting, and Participants:**

This randomized clinical trial included male and female participants aged 18 to 39 years with uncontrolled hypertension confirmed by 24-hour ambulatory blood pressure testing. This was a geographically diverse, multicentered study within 2 large, Midwestern health care systems. Data were collected from October 2017 to February 2022 and analyzed from February to June 2022.

**Interventions:**

The My Hypertension Education and Reaching Target (MyHEART) intervention consisted of telephone coaching every 2 weeks for 6 months, with home blood pressure monitoring. Control participants received routine hypertension care.

**Main Outcomes and Measures:**

The co-primary clinical outcomes were changes in 24-hour ambulatory and clinic systolic and diastolic blood pressure at 6 and 12 months. The secondary outcomes were hypertension control (defined as ambulatory systolic blood pressure <130 mm Hg and diastolic blood pressure <80 mm Hg or clinic systolic blood pressure <140 mm Hg and diastolic blood pressure <90 mm Hg) and changes in hypertension self-management behavior.

**Results:**

A total of 316 participants were randomized (159 to the control group and 157 to the intervention group) from October 2017 to December 2020. The median (IQR) age was 35 (31-37) years, 145 of 311 participants (46.6%) were female, and 166 (53.4%) were male; 72 (22.8%) were Black, and 222 (70.3%) were White. There were no differences in baseline characteristics between groups. There was no significant difference between control and intervention groups for mean 24-hour ambulatory systolic or diastolic blood pressure or clinic systolic or diastolic blood pressure at 6 or 12 months. However, there was appreciable clinical reduction in blood pressures in both study groups (eg, mean [SD] change in systolic blood pressure in intervention group at 6 months, −4.19 [9.77] mm Hg; *P* < .001). Hypertension control did not differ between study groups. Participants in the intervention group demonstrated a significant increase in home blood pressure monitoring at 6 and 12 months (eg, 13 of 152 participants [8.6%] checked blood pressure at home at least once a week at baseline vs 30 of 86 [34.9%] at 12 months; *P* < .001). There was a significant increase in physical activity, defined as active by the Godin-Shephard Leisure-Time Physical Activity Questionnaire, in the intervention group at 6 months (69 of 100 [69.0%] vs 51 of 104 [49.0%]; *P* = .004) but not at 12 months (49 of 86 [57.0%] vs 49 of 90 [54.4%]; *P* = .76). There was a significant reduction in mean (SD) sodium intake among intervention participants at 6 months (3968.20 [1725.17] mg vs 3354.72 [1365.75] mg; *P* = .003) but not 12 months. There were no significant differences in other dietary measures.

**Conclusions and Relevance:**

The MyHEART intervention did not demonstrate a significant change in systolic or diastolic blood pressures at 6 or 12 months between study groups; however, both study groups had an appreciable reduction in blood pressure. Intervention participants had a significant reduction in dietary sodium intake, increased physical activity, and increased home blood pressure monitoring compared with control participants. These findings suggest that the MyHEART intervention could support behavioral changes in young adults with uncontrolled hypertension.

**Trial Registration:**

ClinicalTrials.gov Identifier: NCT03158051

## Key Points

**Question:**

Is a telephone coaching and blood pressure self-monitoring intervention effective in reducing blood pressure compared with usual care among young adults with uncontrolled hypertension?

**Findings:**

In this randomized clinical trial of 316 participants, the intervention did not demonstrate a significant difference in systolic or diastolic blood pressures at 6 or 12 months between the intervention and control groups; however, both groups experienced blood pressure reduction. Compared with the control group, participants in the intervention group demonstrated significant behavior changes, including increased physical activity, reduction in dietary sodium intake, and increased frequency in home blood pressure monitoring.

**Meaning:**

In this study, intervention participants did not experience a significant difference in blood pressure reduction when compared with control participants but did demonstrate behavior changes.

## Teaser

This randomized clinical trial evaluates the effect of the My Hypertension Education And Reaching Target (MyHEART) intervention, a telephone coaching and blood pressure self-monitoring program, compared with usual care on changes in systolic and diastolic blood pressures and behaviors at 6 and 12 months among young adults with uncontrolled hypertension.

## Introduction

More than 10 million individuals aged 18 to 39 years (1 in 5 men; 1 in 6 women) have hypertension,^1,2,3^ which increases the risk of premature heart failure, stroke, and chronic kidney disease.^4,5^ Hypertension control reduces morbidity, mortality, and future health care costs^6,7,8,9,10^; yet less than 50% of young adults have achieved blood pressure (BP) control, even with the former BP guidelines (<140 mm Hg for systolic BP and <90 mm Hg for diastolic BP).^1,11,12,13,14^

Prior to the My Hypertension Education and Reaching Target (MyHEART) program, there were limited clinical trials dedicated to young adults and hypertension control. Previously, trials predominantly targeted adults aged 50 years or older and focused on medication initiation and titration. In contrast, aggressive lifestyle modifications are commonly the initial treatment step among young adults.^1,15^ There is limited guidance on hypertension self-management for young adults.^16,17,18,19^ The content and method of delivery of hypertension self-management must be individualized to young adults and address barriers specific to this population.^20,21,22^ This trial focused on hypertension self-management in young adults, including lifestyle changes, home BP monitoring, and helping young adults understand the role of antihypertensive medication as initiated through clinical care. The MyHEART study tried to address weaknesses in prior studies by (1) targeting barriers identified by young adults in preliminary research, (2) tailoring the mode of delivery to preferences expressed by young adults, and (3) individualizing action plans.

To address the critical need for hypertension control in young adults, we conducted focus groups with young adults documenting barriers and knowledge deficits in key elements of hypertension self-management, which informed development of the MyHEART program. MyHEART is a multicomponent, patient-centered, and theoretically based; it includes 4 evidence-based self-management components: (1) telephone-based health coaching with adult education specialists to teach and monitor self-management skills, (2) documentation of coach-patient telephone contacts, (3) individualized hypertension education materials, and (4) home BP monitoring.^23^ A nonrandomized, single-center pilot study of MyHEART was conducted and established feasibility, satisfaction, and informed the design of this trial.^24^ The primary aim of this study was to evaluate the effect of MyHEART on clinical outcomes, ie, the change in systolic and diastolic BP (primary) and hypertension control (secondary) after 6 and 12 months, compared with usual care.

## Methods

### Trial Design

This multicenter randomized clinical trial was designed to support recruitment of a geographically and racially and ethnically diverse group of young adults. Additional details of the rationale and study design has been previously published.^23^ Institutional review board approval was obtained from the University of Wisconsin–Madison School of Medicine and Public Health, and all participants provided written informed consent. The Consolidated Standards of Reporting Trials (CONSORT) reporting guideline for randomized clinical trials was followed to report this study. Supplement 1 contains the trial protocol.

### Study Outcomes

The co-primary (clinical) outcomes were changes in systolic and diastolic BP after 6 and 12 months. The secondary clinical outcome was hypertension control at 6 and 12 months. Hypertension control was defined as 24-hour ambulatory BP (AMBP) of less than 130 mm Hg for systolic BP and less than 80 mm Hg for diastolic BP or clinic BP of less than 140 mm Hg for systolic BP and less than 90 mm Hg for diastolic BP. Additionally, the effect of MyHEART on hypertension self-management behavioral outcomes was evaluated at 6 and 12 months.

### Participants

Participants were recruited between October 2017 and December 2020 from 2 large, Midwestern health care systems. Potential participants were identified using the electronic health record linked with the Wisconsin Collaborative for Health Quality (WCHQ) hypertension registry.^25,26^ The inclusion criteria included (1) aged 18 to 39 years at enrollment; (2) had a minimum of 2 hypertension *International Statistical Classification of Diseases and Related Health Problems, Tenth Revision *(*ICD-10*) coded office visits on different dates 24 months prior to eligibility assessment, with at least 1 code in the past 18 months; and (3) received their medical care in the study institutions.^27^ Two clinic BP measurements determined eligibility for a study invitation (ie, systolic BP ≥140 mm Hg and/or diastolic BP ≥90 mm Hg),^6^ and the last BP measurement must have been within 90 days. BP measurements from inpatient, emergency department, urgent care, and self-report were excluded. The 2017 American Heart Association/American College of Cardiology guidelines were published during the conduct of this trial, lowering the BP threshold for a diagnosis of hypertension^1^; however the diagnostic BP of 140 mm Hg or greater for systolic BP and/or 90 mm Hg or greater for diastolic BP was maintained throughout the study, as this was the standard diagnostic threshold for hypertension per US guidelines when the study was initiated.^6,12^

Potential participants were mailed an introductory packet summarizing the study with a prepaid opt-out postcard. Individuals who did not opt-out were contacted for telephone screening. If inclusion criteria were met, the individual was invited for an in-person visit (visit 1) for eligibility assessment. Written informed consent was obtained at the start of visit 1. Data on self-reported race (Black, White, or other [Asian, American Indian or Alaska Native, Native Hawaiian or other Pacific Islander, unknown, or not reported]) and ethnicity (Hispanic or non-Hispanic) were collected to describe the population characteristics and to ensure we had a representative and diverse population. BPs were measured with an automated sphygmomanometer (Dinamap; GE)^28^ after confirmation of appropriate cuff size and after 5 minutes seated. Participants were excluded if body habitus was not compatible with cuff sizes. BP readings were acquired in the right and left arm, which determined which arm (highest BP) would be used for future readings. Three BP measurements were then taken on the selected arm, and the mean of the readings defined the baseline systolic and diastolic BPs.^29^ Participants were excluded if the between-arm BP difference was 20 mm Hg or greater.^16,29,30^ All remaining eligible participants who completed visit 1 received placement of a 24-hour AMBP monitor (Space Labs 90277) to confirm a hypertension diagnosis (ie, exclude white coat hypertension).^1,14,31,32^

### Participant Enrollment and Randomization

Participants were eligible if the mean 24-hour AMBP was systolic 130 mm Hg or greater and/or diastolic 80 mm Hg or greater and/or the mean awake AMBP was a systolic 135 mm Hg or greater and/or diastolic 85 mm Hg or greater.^32,33^ Participants were ineligible if the 24-hour AMBP demonstrated white coat hypertension or hypertension control on medications. Eligible participants were enrolled and randomized (visit 2).^34^ Randomization assignments were generated by the statistical data analysis center stratified by research site in block sizes of 4 and 6.^24^

The visit 2 assessment also included self-report of baseline medical history, social history (tobacco [cigarette and vaping], education, alcohol intake), Medicaid coverage, family history, social support, current antihypertension medication use, biometric assessments (height, weight, waist circumference), and baseline self-management behavior of dietary intake and physical activity using validated, self-administered surveys including (1) the National Institute of Health/National Cancer Institute Automated Self-Administered 24-hour (ASA24) Dietary Assessment Tool (NIH-ASA24 Dietary Recall)^35^ and (2) the Godin-Shephard Leisure-Time Physical Activity Questionnaire.^36^

Due to the study design, visit assessors could not be blinded to study assignments.^37,38^ Study coordinators were trained to treat participants in both groups identically. The statistical data assessors were blinded to study assignments.

### Study Protocol

Intervention participants received up to 12 coaching calls (20 min/call), scheduled every 2 weeks for the first 6 months. Coaches were trained on self-determination theory and motivational interviewing. A random sampling of coaches’ calls was assessed with a fidelity checklist, and coaches were given feedback monthly with a behavioral scientist.^39^

Intervention participants received an upper arm BP monitor (Omron, 7 Series) and were instructed to take 2 BP measurements separated by 1 minute, 3 days per week that were shared with the health coach during calls. Detailed instructions on conducting home BP monitoring were provided.^1^ Intervention participants were also instructed to continue receiving usual clinical care.

Control participants received a handout providing an overview on the diagnosis of hypertension at enrollment, and it was recommended to continue receiving usual clinical care^40,41^ This included the possibility of receiving untailored self-management resources (ie, dietician referral) at the clinician’s discretion.^40,41^ Home BP monitors were not provided.

All study participants returned for 6-month (visit 3 and 4) and 12-month (visit 5 and 6) follow-up. These visits included in-office BP measurement; height, weight, and waist circumference measurement; participant-level data collection; and completion of surveys referenced previously. At the end of each visit, participants were fitted with the AMBP monitor to return the following day for interpretation.

### Sample Size Estimation

The power analysis used prior trials to determine clinically meaningful reductions in systolic and diastolic BP.^42^ With an effective sample size of 264, there is power 1 − β of 0.94 and 0.87 for the co-primary 6-month efficacy end points, of a clinically significant mean systolic BP change of 6.4 mm Hg and mean diastolic BP change of 4.0 mm Hg, with SDs of 13.6 and 9.7 mm Hg, respectively, each at a 2-tailed .025 level test for an overall significance level of α = .05. In the pilot study, there was 100% retention through 3 months.^24^ For this trial, a 15% dropout rate was assumed at the 6-month study end point, so the target sample size was increased to 310. The study protocol established patients would be withdrawn after randomization if they no longer received care at the study institution or became pregnant.

### Statistical Analysis

The study analysis followed intention-to-treat principles. Continuous variables are described by mean (SD) and tested with analysis of covariance. Categorical variables are described by count (percentage) and tested with χ^2^ tests, with the exception of education, number of hypertension medications, and self-perceived health status, which were ordered and tested with Mann-Whitney-Wilcoxon tests.

Analysis of covariance was run for the following outcomes: change in systolic and diastolic AMBP at 6 and 12 months and change in systolic and diastolic clinic BP at 6 and 12 months. Paired *t* tests were also performed to assess changes in AMBP and clinic systolic and diastolic BP measurements within each study group. Lastly, a sensitivity analysis was performed including insufficiently active (per the Godin-Shephard Leisure-Time Physical Activity Questionnaire), alcohol use, and sodium intake as indicators for poor health behaviors at baseline for the primary outcome to see whether some subgroups could benefit more from the intervention. Any interaction test resulting in a heterogeneity *P* < .15 may be further evaluated for clinical plausibility. Sequential hypothesis testing was performed for hypertension control at 6 months and 12 months and for BP changes at 12 months using an α-reuse approach. Each co-primary outcome at 6 months between study groups, BP changes from baseline within each group, and secondary outcomes were assessed at the .05 level, and all hypothesis tests were 2-sided. Sequential hypothesis testing was performed for hypertension control at 6 months and 12 months and for BP changes at 12 months using an α-reuse approach. The statistical software used to perform the analysis was R Core Team (2022; R Project for Statistical Computing).

## Results

### Recruitment and Participant Characteristics

**Figure 1.**
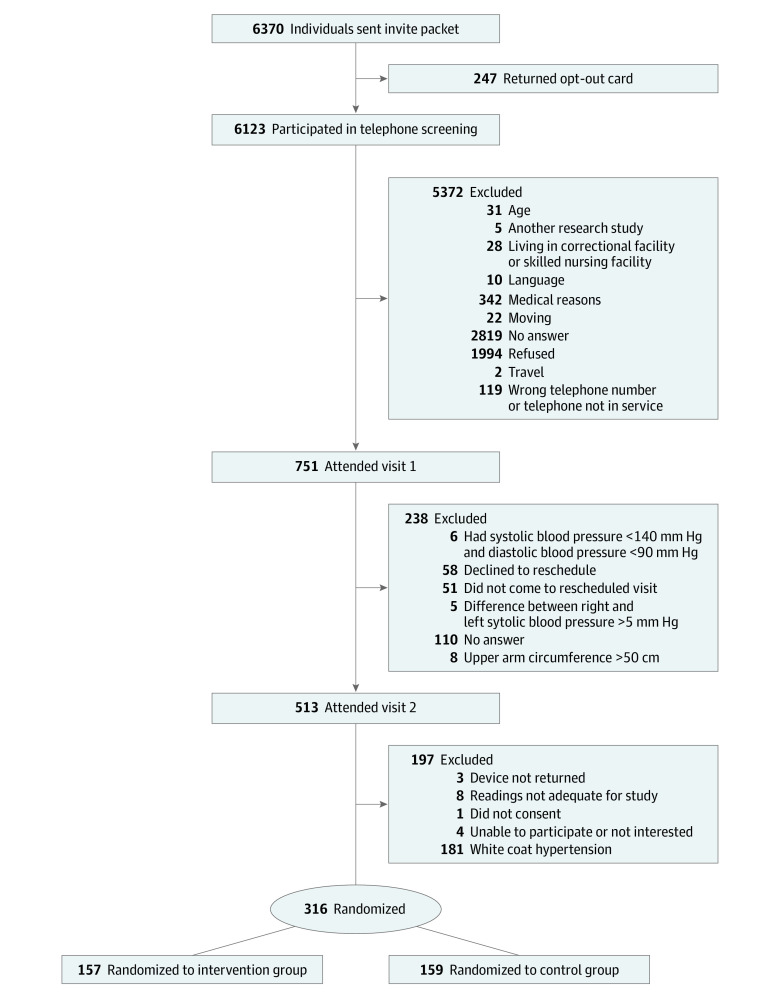
Study Recruitment and Randomization

As outlined in the study flow diagram, 6385 invitations were sent. After completion of preliminary study visits, 316 participants were randomized, with 157 to the intervention group and 159 to the control group. (Figure 1).

### Retention and Adherence

**Figure 2.**
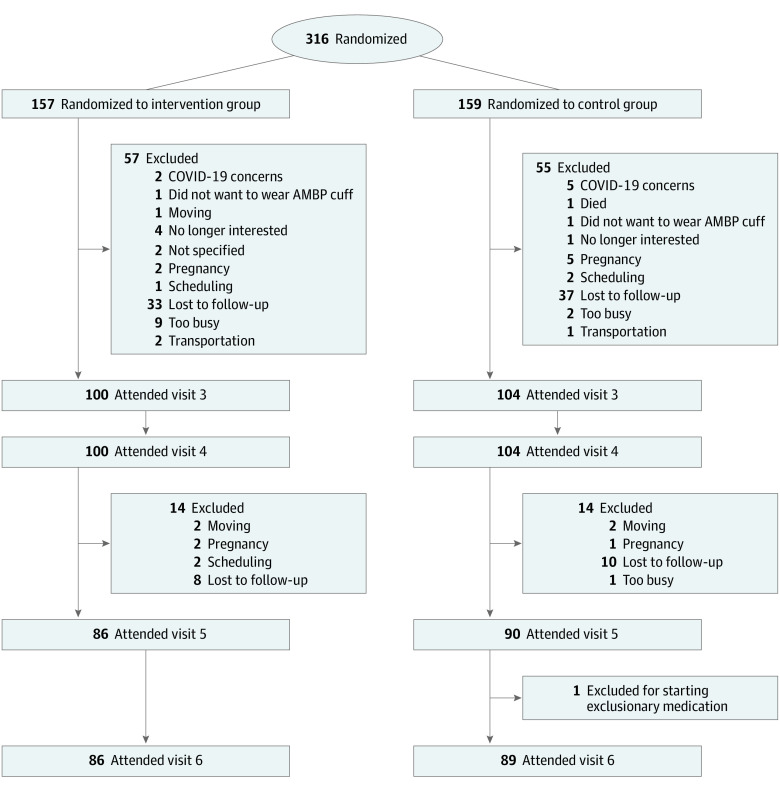
Study Follow-up, Including Detailed Loss to Follow-up AMBP indicates ambulatory blood pressure.

After randomization, 57 intervention participants (36.3%) and 55 control participants (34.6%) were withdrawn, dropped out, or were lost to follow-up by the 6-month end point and an additional 14 intervention participants (8.9%) and 17 control participants (10%) by the 12-month end point. There were 86 intervention and 89 control participants who completed the 12-month study visits. Detailed reasons for study dropout are detailed in Figure 2. The reasons for dropout were similar by study group and did not appear to differ before and after the COVID-19 pandemic. The study team did not have information on causes for loss to follow-up. In comparison with those retained at 6 months, we discovered those who did not complete the study were more likely to belong to a minoritized racial or ethnic group and use tobacco cigarettes (eTable 1 in Supplement 2).

### Primary Results

A total of 316 participants (159 control and 157 intervention) were randomized. There was equal distribution of male (166 [52.5%]) and female (145 [45.9%]) participants; 72 (22.8%) were Black, and 222 (70.3%) were White. The median (IQR) age was 35 (31-37) years. The baseline mean (SD) 24-hour AMBPs were systolic 133.00 (12.67) mm Hg and diastolic 87.04 (8.16) mm Hg for the control group and systolic 132.59 (11.36) mm Hg and diastolic 86.45 (7.76) mm Hg for the intervention group. There were no significant differences in baseline sociodemographic or health behavior characteristics. At baseline, 162 participants (51.3%) were not receiving antihypertensive medication (78 [49.1%] control and 84 [53.5%] intervention) (Table 1).

**Table 1.** Summary of Baseline Characteristics of Intervention and Control Participants

Characteristic	Participants, No. (%)		
	Overall (N = 316)	Control (n = 159)	Intervention (n = 157)
Age, median (IQR), y	35 (31-37)	35 (30-38)	35 (31-37)
Sex			
Female	145 (45.9)	74 (46.5)	71 (45.2)
Male	166 (52.5)	85 (53.5)	81 (51.6)
Race			
Black	72 (22.8)	35 (22.0)	37 (23.6)
Other^a^	22 (7.0)	9 (5.7)	13 (8.3)
White	222 (70.3)	115 (72.3)	107 (68.2)
Ethnicity			
Hispanic	16 (5.1)	8 (5.0)	8 (5.1)
Non-Hispanic	300 (94.9)	151 (95.0)	149 (94.9)
Alcohol beverages/wk, median (IQR)	3 (1-7)	3 (1-6)	3 (1-8)
ASA24, median (IQR)			
Saturated fat, % of kcal	12 (9-15)	12 (10-15)	12 (9-14)
Sodium intake, mg	3316 (2420-4400)	3446 (2462-4568)	3213 (2293-4281)
Whole grain, oz	0 (0-1.2)	0 (0-1.4)	0 (0-0.8)
Fruit and vegetable servings, cup equivalent	2.0 (1.0-3.4)	2.0 (1.0-3.5)	2.0 (1.0-3.2)
Ambulatory BP, median (IQR), mm Hg			
Diastolic	85.0 (81.0-90.2)	85.0 (81.0-90.0)	85.0 (82.0-91.0)
Systolic	131.0 (125.0-138.0)	131.0 (124.0-138.0)	131.0 (125.0-138.0)
BMI, median (IQR)	32.8 (28.0-39.5)	33.1 (28.0-39.2)	32.3 (27.9-39.7)
No. of children at home, median (IQR)	0 (0-2)	0 (0-2)	0 (0-2)
Clinic BP, median (IQR), mm Hg			
Diastolic	90.0 (83.7-97.8)	90.3 (84.3-97.2)	89.3 (82.3-98)
Systolic	135.0 (128-146)	135.0 (128-143)	136.0 (129-148)
Waist circumference, median (IQR), cm	106 (97-122)	106 (96-124)	105 (97-120)
Weight, median (IQR), kg	99 (83-119)	99 (83.5-122)	98 (83-116)
Cigarette tobacco status			
I currently smoke cigarettes	40 (12.7)	19 (11.9)	21 (13.4)
I have never smoked cigarettes	212 (67.1)	115 (72.3)	97 (61.8)
I used to smoke cigarettes	59 (18.7)	25 (15.7)	34 (21.7)
Godin-Shephard Leisure-Time Physical Activity			
Active	149 (47.2)	81 (50.9)	68 (43.3)
Insufficiently active	162 (51.3)	78 (49.1)	84 (53.5)
Highest level of education			
I have not finished high school	15 (4.7)	8 (5.0)	7 (4.5)
I have completed high school	42 (13.3)	20 (12.6)	22 (14.0)
I have not finished college or vocation school	52 (16.5)	24 (15.1)	28 (17.8)
I finished college or vocational school	137 (43.4)	74 (46.5)	63 (40.1)
I have attended some or completed graduate or professional school	65 (20.6)	33 (20.8)	32 (20.4)
Marital status			
Single	104 (32.9)	54 (34.0)	50 (31.8)
Married or partnered	200 (63.3)	101 (63.5)	99 (63.1)
Divorced or widowed	7 (2.2)	4 (2.5)	3 (1.9)
No. of antihypertensive medications			
0	162 (51.3)	78 (49.1)	84 (53.5)
1	97 (30.7)	51 (32.1)	46 (29.3)
2	43 (13.6)	21 (13.2)	22 (14.0)
3	11 (3.5)	8 (5.0)	3 (1.9)
≥4	3 (0.9)	1 (0.6)	2 (1.3)
Self-perceived health status			
Excellent	7 (2.2)	4 (2.5)	3 (1.9)
Very good or good	153 (48.4)	83 (52.2)	70 (44.6)
Fair	111 (35.1)	50 (31.4)	61 (38.9)
Poor or no response	39 (12.3)	21 (13.2)	18 (11.5)
Comorbidities			
Anxiety and/or depression	160 (50.6)	78 (49.1)	82 (52.2)
Chronic kidney disease	3 (0.9)	2 (1.3)	1 (0.6)
Diabetes	18 (5.7)	9 (5.7)	9 (5.7)
Dyslipidemia	73 (23.1)	32 (20.1)	41 (26.1)
Other chronic comorbidity	121 (38.3)	59 (37.1)	62 (39.5)
Risk factors			
e-Cigarette or vaping use in past 6 mo	26 (8.2)	13 (8.2)	13 (8.3)
Ever received Medicaid	67 (21.2)	33 (20.8)	34 (21.7)
Family history of heart disease or stroke	113 (35.8)	61 (38.4)	52 (33.1)
Financial status: inadequate income	248 (78.5)	122 (76.7)	126 (80.3)

Abbreviations: ASA24, Automated Self-Administered 24-hour; BMI, body mass index (calculated as weight in kilograms divided by height in meters squared); BP, blood pressure.

^a^ Other race was a composite of the following self-reported races: American Indian or Alaska Native, Asian, Native Hawaiian or other Pacific Islander, unknown, not reported, or other.

At 6 months, there was no significant difference between the control and intervention groups for mean 24-hour AMBP systolic (130.69 [13.99] mm Hg vs 128.14 [11.36] mm Hg; *P* = .12) or diastolic (85.89 [9.19] mm Hg vs 84.61 [8.24] mm Hg; *P* = .16) BP at 6 months or clinic systolic (132.50 [15.83] mm Hg vs 130.84 [12.16] mm Hg; *P* = .44) or diastolic (86.70 [12.44] mm Hg vs 83.83 [9.36] mm Hg; *P* = .20) BP. Additionally, no differences were identified between groups at 12 months (Table 2).

**Table 2.** Summary of Clinic Blood Pressure and AMBP at Baseline, 6-Month, and 12-Month Visits

Visit	Participants, No.	Mean (SD)			
		Measured BP, mm Hg^a^		Change from baseline^b^	
		Systolic	Diastolic	Systolic	Diastolic
**AMBP**					
Baseline					
Control	159	133.01 (12.67)	87.04 (8.16)	[Reference]	[Reference]
Intervention	157	132.59 (14.27)	86.45 (7.76)	[Reference]	[Reference]
6-mo					
Control	102	130.69 (13.99)	85.89 (9.19)	−2.20 (10.72)^c^	−0.74 (7.86)^d^
Intervention	100	128.14 (11.36)	84.61 (8.24)	−4.19 (9.77)^e^	−2.17 (7.04)^f^
*P* value	NA	.12	.16	NA	NA
12-mo					
Control	88	129.47 (14.71)	85.52 (9.43)	−2.23 (13.82)^d^	−0.36 (8.94)^d^
Intervention	85	128.54 (11.95)	85.34 (9.32)	−3.21 (10.72)^f^	−1.14 (8.52)^d^
ANCOVA *P* value		.59	.58	NA	NA
**Clinic**					
Baseline					
Control	159	137.97 (14.33)	91.77 (11.26)	[Reference]	[Reference]
Intervention	157	138.30 (14.27)	90.81 (11.42)	[Reference]	[Reference]
6-mo					
Control	104	132.50 (15.83)	86.70 (12.44)	−5.59 (14.29)^e^	−4.86 (9.79)^e^
Intervention	100	130.84 (12.16)	83.83 (9.36)	−6.62 (12.52)^e^	−5.70 (9.87)^e^
ANCOVA *P* value	NA	.41	.20	NA	NA
12-mo					
Control	90	130.7 (15.22)	85.15 (12.31)	−6.41 (14.67)^e^	−5.56 (12.22)^e^
Intervention	86	131.16 (12.77)	85.05 (10.56)	−5.78 (13.53)^e^	−4.29 (10.31)^e^
ANCOVA *P* value	NA	.78	.69	NA	NA

Abbreviations: AMBP, ambulatory BP; ANCOVA, analysis of covariance; BP, blood pressure; NA, not applicable.

^a^ ANCOVA test was used to compare measured BP at each point between study groups.

^b^ Paired *t* test was used to compare changes from baseline in BP within each study group.

^c^ *P* ≤ .05.

^d^ Not significant.

^e^ *P* ≤ .01.

^f^ *P *≤ .001.

In both study groups, there was an appreciable decrease from baseline 6- and 12-month systolic and diastolic clinic BPs (eg, mean [SD] change in systolic blood pressure in intervention group at 6 months, −4.19 [9.77]; *P* < .001) (Table 2). In a similar analysis to assess for changes in systolic and diastolic BP within study groups, significant reductions were identified from baseline clinic BP and 24-hour AMBP across all time points, except for diastolic BP at 12 months. The control group demonstrated a significant reduction in only clinic BP assessments across all time points. For 24-hour AMBP values in the control group, no significant reductions were found, except for systolic BP at 6 months (Table 2).

The sensitivity analysis suggests participants who were insufficiently active at baseline had larger decreases in systolic and diastolic 24-hour AMBP at 6 months. It also appears that alcohol use at baseline was associated with larger decreases in AMBP systolic and diastolic at 6 months (eTable 3 in Supplement 2). This change did not persist in any of the clinical BPs or at 12 months for any AMBP measures.

### Prespecified Secondary Outcomes

Hypertension control improved in both groups throughout the study; however, there was no significant change comparing groups. Hypertension control using AMBP values of systolic BP less than 130 mm Hg and diastolic BP less than 80 mm Hg between study groups was achieved in 21 of 102 control participants (20.6%) and 20 of 100 intervention participants (20.0%) at 6 months (*P* > .99) and 18 of 88 control participants (20.5%) and 18 of 85 intervention participants (21.2%) at 12 months (*P* > .99). Hypertension control using clinic BP values of systolic BP less than 140 mm Hg and diastolic BP less than 90 mm Hg among study groups were 60 of 104 control participants (57.7%) and 60 of 100 intervention participants (60.0%) at 6 months (*P* = .77) and 54 of 90 control participants (60.0%) and 56 of 86 intervention participants (65.1%) at 12 months (*P* = .54). Use of antihypertensive medication from baseline to 6 and 12 months increased in both study groups. At baseline, 73 of 152 intervention participants (48.0%) and 81 of 159 control participants (51.0%) of the control group reported use. This increased to 59 of 100 (59.0%) and 59 of 104 (56.7%) at 6 months and 50 of 86 (58.1%) and 52 of 90 (57.8%) at 12 months. Due to the higher than anticipated rates of dropout, these data should be interpreted with caution.

There were significant changes in hypertension self-management behaviors between groups. There was an increase in home BP monitoring between the intervention and control groups at 6 and 12 months (eg, 13 of 152 participants [8.6%] checked blood pressure at home at least once a week at baseline vs 30 of 86 [34.9%] at 12 months; *P* < .001) (Table 3). There was a significant difference in mean (SD) dietary sodium intake at 6 (3968.20 [1725.17] mg vs 3354.72 [1365.75] mg; *P* = .003) but not 12 months (4213.67 [1972.37] mg vs 3682.94 [1874.53] mg; *P* = .14). There was also a difference in physical activity using Godin-Shephard Leisure-Time Physical Activity Questionnaire active status at 6 months (69 of 100 [69.0%] vs 51 of 104 [49.0%]; *P* = .004) but not at 12 months (49 of 86 [57.0%] vs 49 of 90 [54.4%]; *P* = .76). No significant changes were identified in mean (SD) weight between study groups at 6 months (103.80 [25.34] kg vs 98.54 [26.19] kg; *P* = .43) or 12 months (103.45 [25.49] kg vs 97.91 [26.07] kg; *P* = .39). There was no difference in combined fruit and vegetable servings, whole grain intake, or saturated fat intake at 6 or 12 months (eTable 2 in Supplement 2).

**Table 3.** Home Blood Pressure Monitoring Frequency

Frequency	No./total No. (%)		
	Baseline	6-mo	12-mo
**Intervention group**			
1, Not on a regular basis	109/152 (71.7)	14/100 (14)	32/86 (37.2)
2, At least once per month	23/152 (15.1)	9/100 (9)	20/86 (23.3)
3, At least once a week	13/152 (8.6)	64/100 (64)	30/86 (34.9)
4, At least once a day	7/152 (4.6)	13/100 (13)	4/86 (4.7)
Missing	5	57	71
**Control group**			
1, Not on a regular basis	118/159 (74.2)	67/104 (64.4)	67/90 (74.4)
2, At least once per month	20/159 (12.6)	22/104 (21.2)	11/90 (12.2)
3, At least once a week	17/159 (10.7)	10/104 (9.6)	8/90 (8.9)
4, At least once a day	4/159 (2.5)	5/104 (4.8)	4/90 (4.4)
Missing	0	55	69
**Difference between groups** ^a^			
*P* value, raw score	.63	<.001	<.001
*P* value, change from baseline	NA	<.001	<.001

Abbreviation: NA, not applicable.

^a^ Blood pressure monitoring frequency with Mann-Whitney-Wilcoxon *P* values.

### Health Coaching

The mean (SD) duration for health coaching calls was 19.8 (6.8) minutes. Participants who reached the 6-month study end point participated in more mean (SD) coaching calls compared with participants who did not (10.89 [2.81] vs 4.93 [4.14] of 12 total calls). The random sampling of coaches’ calls demonstrated the majority of call items assessed were met, demonstrating high fidelity to the coaching protocol.

### Adverse Events

Adverse events were experienced by 41 control participants (26.1%) and 56 intervention participants (35.2%). The most commonly reported adverse event was skin and subcutaneous tissue irritation from the 24-hour AMBP cuff (24 control participants and 31 intervention participants), which has been previously reported.^43^ Most remaining events were deemed unrelated to the study, and participants recovered without sequelae.

## Discussion

### Summary of Findings

The MyHEART study represents successful recruitment, enrollment, and engagement of racially and geographically diverse young adults. The trial did not demonstrate a significant change between study groups in systolic or diastolic BP or the secondary outcome of hypertension control at 6 or 12 months. However, both study groups demonstrated an overall decrease in systolic and diastolic BPs at 6 and 12 months. The interval change in clinic and 24-hour AMBP systolic and diastolic from baseline to 6 and 12 months was evaluated, and we found the intervention group had a significant reduction in all 6- and 12-month BP values except the 12-month 24-hour AMBP results in comparison with the baseline values. The control group demonstrated a significant reduction in all of the clinic BP values and only the systolic 24-hour AMBP result at 6 months. Compared with the control group, intervention participants demonstrated a significant increase in physical activity, reduction in dietary sodium intake, and increase in home BP monitoring at 6 months. The increase in physical activity and reduction in dietary sodium intake was not maintained at 12 months. Participants still had an increase in performing home BP monitoring at 12 months, albeit at a lower rate.

### BP Values at End Points

There was always a lower BP value in the intervention group compared with the control group during follow-up, with the exception of the 12-month clinic systolic BP. The fact that the control group only demonstrated a significant reduction in clinic BP results and systolic AMBP values may suggest that using only clinic BP measurements may be misleading, as they are a cross-sectional value at a single point compared with the larger range of BP measurements acquired with 24-hour AMBP testing. Given the limited feasibility of 24-hour AMBP, this highlights the importance of incorporating home BP monitoring in hypertension management for a more accurate reflection of BP values across time.

Because between study-group differences in BPs were not observed and both study groups improved hypertension control over time, an explanation is warranted. First, the loss to follow-up in this trial was similar in both study groups but 2-fold higher at 6 months than accounted for in our sample size calculation. The study team cannot be sure that that the null results are due to the higher than expected dropout rates; however, a meaningful reduction in BPs in both study groups is appreciated. Second, the MyHEART study design was such that participants received three 24-hour AMBP and 3 in-office study visit BP assessments (at baseline, 6 months, and 12 months). Although the research staff followed protocols, the staff communicated with participants regarding BP results and weight. Perhaps the staff communications served to stimulate motivation or behaviors in the comparison group. These evaluations may have resulted in more attention to both study groups’ hypertension, in addition to prompt potential interactions with the participants’ usual care team. Although enhanced care beyond recommending engagement with routine care with a health care professional was not provided, the unexpected decrease in BPs in both study groups, specifically the control group, may be due to the Hawthorne effect, which refers to the alteration of study participant behavior solely due to being observed rather than the result of an intervention.

The rate of clinical hypertension control among study participants at the 6- and 12-month study end points in both study groups and was higher than national estimates, where less than 50% of young adults with hypertension in the US have achieved BP control, even with the former BP guidelines (systolic <140 mm Hg and diastolic <90 mm Hg).^1,11,12,13,14^ On a larger population level, this may have a significant effect, as a 2–mm Hg decrease in systolic BP is estimated to lower mortality from stroke and ischemic heart disease.^44^

Per US guidelines, lifestyle modifications are recommended for the treatment of stage 1 hypertension, in populations with a 10-year atherosclerotic cardiovascular disease risk score of less than 10% and without diabetes or chronic kidney disease.^1^ An overall increase in antihypertension medication use in both groups at the 6- and 12-month study end points was observed when compared with baseline, which supports a combined effort in lifestyle modifications and medication use if lifestyle modifications alone are not effective. These findings are supported by prior studies demonstrating improved hypertension control rates with more frequent hypertension follow-up and with increased awareness of BP outside the clinical setting.^45,46^ Physical activity, sodium reduction, and home BP monitoring are guideline-recommended health behavior changes to improve hypertension control.

### Limitations

This study has limitations. The dropout rate was higher than expected but did not differ by study group. The latter part of the study overlapped with the COVID-19 pandemic; however, there was greater study dropout or loss to follow-up prior to the pandemic. It is possible that stay-at-home orders may have provided participants with more time to invest in health or research studies. The reasons identified for study dropout were more likely related to social determinants of health and other routine life factors experienced in the young adult population, such as pregnancy, relocation, or the perception of being too busy. Transportation was also a reported reason for study dropout. In consideration of future trial designs and successful trial conduct in young adults, the study team recommends promoting ongoing engagement opportunities throughout the study and identifying and developing resources for supporting social determinants of health as well as social and community support unique to young adults.

Although our intervention did not include prescribing medication, the objective of this study was to demonstrate effective and sustainable resources to support routine clinical care. Since approximately 50% of participants from both groups were not receiving antihypertensive medication at baseline, future interventions combining components of the MyHEART program in a clinic with a partnered clinician to prescribe guideline-directed antihypertensive medication initiation and/or stepped titration may demonstrate more significant BP lowering and hypertension control.^1^ Additional limitations include the use of self-report survey for behavioral changes (eg, dietary changes). However, the study was designed to limit participant burden with additional testing for sodium assessment.

## Conclusions

The MyHEART intervention did not demonstrate a significant change in systolic and diastolic BP measurements or hypertension control at 6 or 12 months when compared with control participants. However, both groups had a reduction in systolic and diastolic BP and an increase in hypertension control. Intervention participants had a significant increase in active physical status, reduction in dietary sodium intake, and increase in home BP monitoring. These findings suggest that incorporating components of the MyHEART intervention with routine clinical care can support hypertension behavioral changes in young adults with uncontrolled hypertension to support BP lowering in this challenging population.

## Notes

Supplement 1.
Trial Protocol

Supplement 2. **eTable 1. **Baseline Participant Characteristics **eTable 2.** ASA-24 Dietary Component Evaluation of Sodium, Combined Fruit and Vegetables, Whole Grain, and Saturated Fat Intake, Comparing Baseline With 6 and 12 Months **eTable 3.** Sensitivity Analyses

Supplement 3.
Data Sharing Statement
