# Supplementary material for: Effect of a Telephone Health Coaching Intervention on Hypertension Control in Young Adults: The MyHEART Randomized Clinical Trial
Source: JAMA Netw Open. 2023 Feb 3;6(2):e2255618. doi: 10.1001/jamanetworkopen.2022.55618 (PMC9898821; doi:10.1001/jamanetworkopen.2022.55618)
Supplement: Supplement 1. — Trial Protocol [file jamanetwopen-e2255618-s001.pdf]

THE MYHEART STUDY: A YOUNG ADULT HYPERTENSION SELF-MANAGEMENT RANDOMIZED CONTROLLED TRIAL

A randomized, multi-center study of MyHEART (My Hypertension Education And Reaching Target) on the change in systolic and diastolic blood pressure, hypertension control and hypertension self-management behavior compared to usual clinical care in young adults (18-39 years) with hypertension.

Principal Investigator: Kara Hoppe

This document is confidential.
No part of it may be transmitted, reproduced, published, or used by other persons without prior written authorization from the Sponsor or Principal Investigator.

PROTOCOL VERSION and AMENDMENTS

Protocol Version	Date	Change Initiated (Initials)	Brief description of protocol modification/actions requested, if any
Template V6	9/6/16	TNK	Input from IRB, OCT, MARCH, IND/IDE services, PIs
V1	3/23/17	HJ, JL, VC (HIP); TNK (OCT)	Version sent to the IRB with initial application
V2	4/4/17	HJ, VC, JL (HIP)	Responded to IRB's pre-review comments
V3	4/19/17	HJ, JL, VC (HIP)	Responded to IRB committee's modification requests
V4	5/16/2017	HJ, JL, VC (HIP)	Aurora Health Care added as a study site
V5	6/22/17	JL, HJ (HIP)	Responded to IRB's pre-review comments
V6	12/4/17	HJ	Protocol change
V7	9/10/18	HJ	Protocol change
V8	11/22/19	HJ	Principal Investigator Change from Heather Johnson to Kara Hoppe
V9	04/28/22	SB	Change of storage of study documents

STATEMENT OF COMPLIANCE

The research will be conducted in accordance with the Code of Federal Regulations on the Protection of Human Subjects (45 CFR Part 46), and the NIH NHLBI Terms of Award. The Principal Investigator will assure that no deviation from, or changes to the protocol will take place without documented approval from the Institutional Review Board (IRB), except where necessary to eliminate an immediate hazard(s) to the trial participants. All personnel involved in the conduct of this study have completed Human Subjects Protection Training.

I agree to ensure that all staff members involved in the conduct of this study are informed about their obligations in meeting the above commitments.

Principal Investigator: _Kara Hoppe, DO
Print/Type Name

Signed: ______________________

**Funding Sponsor:**	National Heart, Lung, and Blood Institute<br>NHLBI Health Information Center<br>P.O. Box 30105<br>Bethesda, MD 20824-0105<br>(301) 592-8573
**Study Product:**	Not applicable
**Protocol Number:**	UW-Madison HS IRB#: 2017-0372<br>Aurora site approval UW-Madison HS IRB#: 2017-0372 -CP001
**IND/IDE Number:**	Not applicable
**Participating sites:**	UW Health; Madison, WI<br>Aurora Health Care; Milwaukee, WI

Figure. MyHEART R01 Organizational Chart as submitted to NIH/NHLBI

Note: This protocol reflects the addition of the Aurora Health Care site and approval from Aurora Health Care and the Wisconsin IRB Consortium (WIC) to allow UW-Madison to serve as the IRB of record for this study. The appendix has also been updated to include Aurora site-specific recruitment and consent materials.

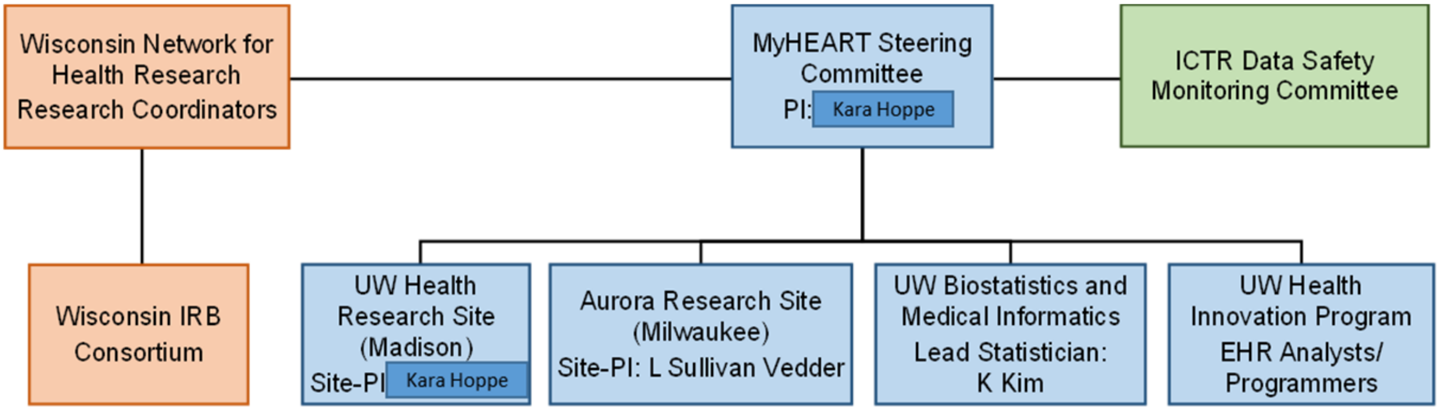

MyHEART is a randomized, controlled trial with two IRB-approved participating healthcare systems. The Steering Committee is the primary decision-making body for the MyHEART trial. Membership to the Steering Committee includes the study principal investigator (PI) and site-PI, lead statistician from the UW Biostatistics and Medical Informatics, research coordinators from the Wisconsin Network for Health Research, and data programmers/analysts from the UW Health Innovation Program (HIP). The ICTR Data Safety and Monitoring Committee (ICTR DSMC; https://ictr.wisc.edu/DMC) will serve as an independent data and safety monitoring board.

All versions of this protocol must be approved by the UW Health Sciences IRB. At the start of the MyHEART trial, study binders will be set up at each study site with the current protocol version (will include protocol number and date), and it will be available on a secure study intranet through HIP. If changes are necessary for the project, protocol modifications will be submitted to the UW Health Sciences IRB to change the protocol to fit the required changes. When the UW Health Sciences IRB approves amendments to the protocol, a copy of the approval will accompany the

summary of changes approved and the new protocol version sent to the study sites. Electronic copies of the approval documents will be forwarded to each site with the other electronic documents and stored electronically and in paper form in an IRB folder at each study site. At each WiNHR coordinator conference call, reconciliation of all current approvals will be performed by all the investigators in their study binders and electronic files.

List of Abbreviations

**AE**	Adverse event
**AMBP**	ambulatory blood pressure
**ASA24**	Automated Self-Administered 24-hour Dietary Assessment
**BCG**	Bioinformatics Computing Group
**BMI**	body mass index
**BP**	blood pressure
**CARDIA**	Coronary Artery Risk Development in Young Adults
**cm**	centimeters (used in measurements of height)
**CV**	cardiovascular
**DASH**	Dietary Approaches to Stop Hypertension diet
**DBP**	diastolic blood pressure
**DMC**	data monitoring committee
**DO**	doctor of osteopathic medicine
**DSMB**	Data Safety and Monitoring Board
**DSMP**	Data Safety and Monitoring Plan
**eCRF**	electronic case report forms
**EHR**	electronic health record
**FDA**	Food and Drug Administration
**HCCQ**	Health-Care Climate Questionnaire
**HEDIS**	Healthcare Effectiveness Data and Information Set
**HIP**	Health Innovation Program (at UW-Madison)
**HIPAA**	Health Insurance Portability and Accountability Act
**ICD-10**	International Classification of Disease, Tenth Edition
**ICTR**	Institute for Clinical and Translational Research (at UW-Madison)
**IRB**	Institutional Review Board
**J2EE**	Java 2 Platform, Enterprise Edition
**JDBC API**	Java Database Connectivity Application Program Interface
**JNC V**	The Fifth Report of the Joint National Committee on the Detection, Evaluation and Treatment of High Blood Pressure
**JNC VI**	The Sixth Report of the Joint National Committee on Prevention, Detection, Evaluation, and Treatment of High Blood Pressure
**JNC VII**	The Seventh Report of the Joint National Committee on Prevention, Detection, Evaluation, and Treatment of High Blood Pressure
**kcal/kg/hr**	kilo calories per kilogram per hour (used in the measurement of METs; one MET is defined as 1 *kcal/kg/hr* and is roughly equivalent to the energy cost of sitting quietly)
**kg**	kilograms (used in measurements of weight)
**kg/m^2**	kilograms per meters squared (used in measurements of body mass index)
**MD**	medical doctor
**MET**	Metabolic Equivalent
**mmHg**	millimeters of Mercury (used in measurements of blood pressure

**MyHEART**	My Hypertension Education and Reaching Target
**NHANES**	National Health and Nutrition Examination Survey
**NP**	nurse practitioner
**OSA**	obstructive sleep apnea
**PA**	physician assistant
**PCS**	Perceived Competence Scale
**PI**	principal investigator(s)
**REALM-R**	Rapid Estimate of Adult Literacy in Medicine-Revised
**REDCap**	Research Electronic Data Capture
**SAE**	serious adverse event
**SBP**	systolic blood pressure
**SDT**	self-determination theory
**SV**	Screening Visit
**TSRQ**	Treatment Self-Regulation Questionnaires
**USDA**	United States Department of Agriculture
**UW**	University of Wisconsin
**WCHQ**	Wisconsin Collaborative for Healthcare Quality
**WIC**	Wisconsin IRB Consortium
**WiNHR**	Wisconsin Network for Health Research

Study Summary

Title	The MyHEART Study: A Young Adult Hypertension Self-Management Randomized Controlled Trial
Short Title and Precis	MyHEART R01
Protocol Number	UW-Madison HS IRB#: 2017-0372
ClinicalTrials.gov number	ClinicalTrials.gov Identifier: NCT03158051
Phase	not applicable
Methodology	Randomized controlled Trial
Study Duration	5 years
Study Center(s)	Two Healthcare Systems<br>- UW Health (Madison, WI)<br>- Aurora Health Care (Milwaukee, WI)
Objectives	Aim 1. To evaluate the effect of MyHEART (home blood pressure monitor distribution and heath coaching) on clinical outcomes, the change in systolic and diastolic blood pressure (primary) and hypertension control (secondary) after 6 and 12 months, compared to usual clinical care. We hypothesize that MyHEART will significantly decrease systolic and diastolic blood pressures (clinic and 24-hour ambulatory) in young adults, compared to usual clinical care.<br>Aim 2. To evaluate the effect of MyHEART on hypertension self-management behavior (behavioral outcomes) at 6 and 12 months, compared to usual clinical care. We hypothesize that MyHEART will increase the frequency of home blood pressure monitoring and lifestyle modifications (increased physical activity, decreased sodium intake).<br>Aim 3. To examine whether MyHEART's effects on self-management behavior are mediated through variables of perceived competence, autonomy, motivation, and activation (mediation outcomes). Based on our pilot study and theoretical framework, we hypothesize that MyHEART's effects will be mediated through perceived competence, autonomy, internal motivation, and patient activation.
Number of Subjects	N=310
Diagnosis	uncontrolled hypertension
Main Inclusion Criteria	18-39 years with a diagnosis of "elevated blood-pressure reading, without diagnosis of hypertension" or a hypertension diagnosis, uncontrolled blood pressure (≥140/90 mmHg), and medically homed at one of the included clinical sites
Main Exclusion Criteria	Any of the following diagnoses: chronic kidney disease, congestive heart failure, sickle cell anemia, cystic fibrosis, stroke, myocardial infarction, coronary artery revascularization, or prior/planned organ transplant; inability to provide informed consent or read or communicate in English; residence at skilled nursing or correctional facility; prescription of warfarin, novel oral anticoagulant, planned chemotherapy, planned radiation therapy, plan to move out of area in next 6 months; pregnant or plan to become pregnant in next year; illegal drug use other than marijuana in past 30 days; syncope in past 12 months
Study Product, Dose, Route, Regimen	Health coach visits, with follow-up telephone calls, self-management support, and primary care provider feedback
FDA status of product	not applicable

Duration of administration	6-month intervention; 6-month maintenance evaluation (12-month total follow-up)
Reference therapy	Usual Clinical Care
Statistical Methodology	The primary comparisons for the co-primary outcomes of systolic and diastolic blood pressure change from baseline to 6 months will be done using analysis of covariance with MyHEART and baseline blood pressure as independent covariates. The primary comparisons for the secondary outcome of hypertension control will be based on Fisher's exact tests. Mediation analysis will be performed for Aim 3.

Schematic of Study Design

Figure: Eligibility, Enrollment, Randomization, Follow-up and Analysis

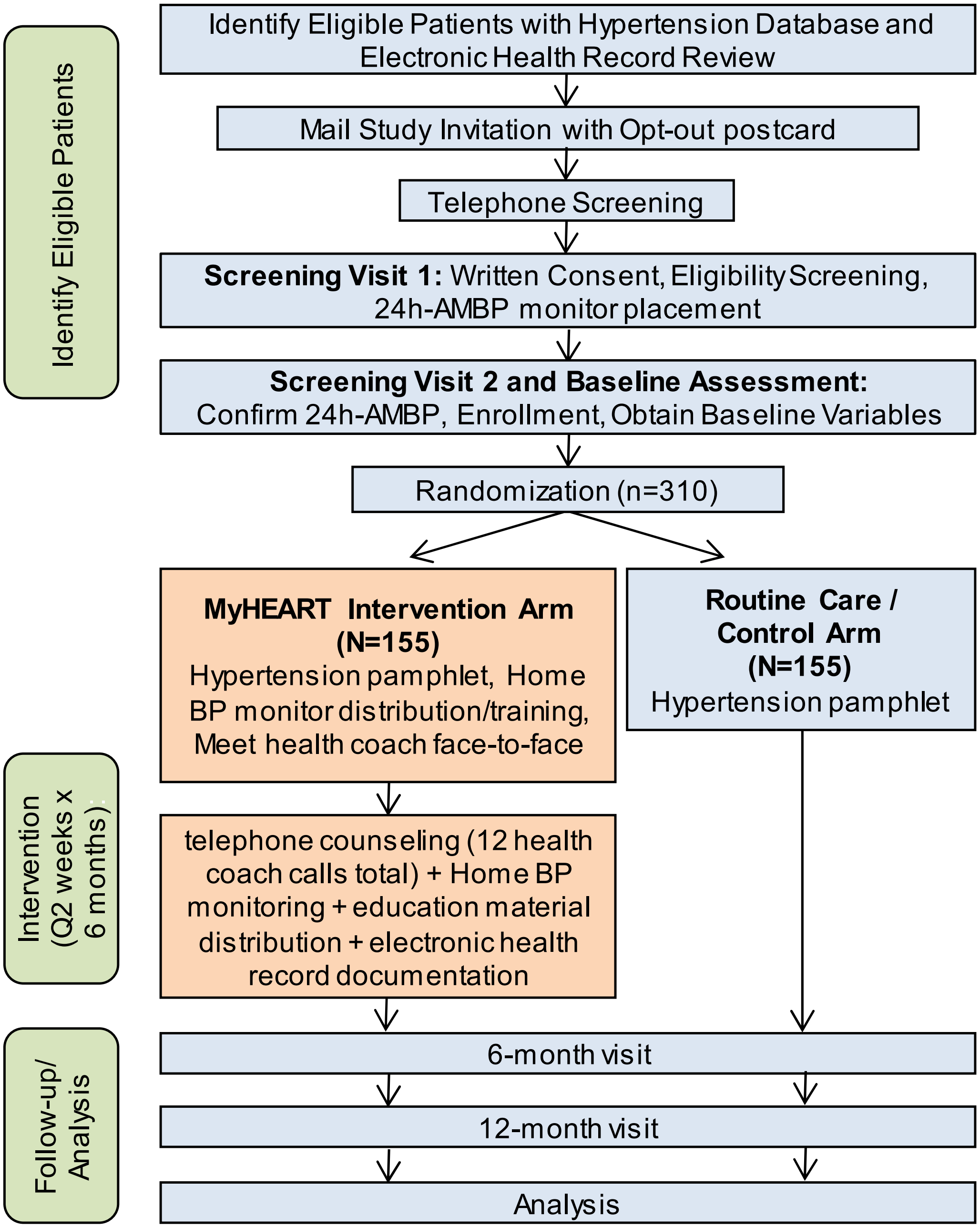

Contents

Study Summary 6
1 Key Roles 12
2 Background and Introduction 12
2.1 Background and Rationale 13
2.1.1 Development of MyHEART 13
2.2 Hypothesis 14
2.3 Study Agent 14
2.4 Summary of Clinical Data 15
2.5 Potential Risk and Benefits to Subjects 16
2.5.1 Known Potential Risks 16
2.5.2 Alternative to Study Participation 17
2.5.3 Protection Against Risks 17
2.5.4 Adverse Event Reporting 19
2.5.5 Potential Benefits to the Subjects 20
2.5.6 Risk Minimization 20
3 Study Design and Endpoints 21
3.1 General Design 21
3.1.1 Primary Study Endpoints 22
3.1.2 Secondary Study Endpoints 22
3.1.3 Primary Safety Endpoints 22
4 Study Subjects – Enrollment and Withdrawal 22
4.1 Subject Population 22
4.2 Inclusion Criteria 23
4.3 Exclusion Criteria 23
4.4 Subject Screening for Recruitment 24
4.4.1 Subject Identification 24
4.4.2 Recruitment and Retention Strategies 24
4.5 Vulnerable Populations 25
4.5.1 Subject Capacity 25
4.5.2 Subject/Representative Comprehension 25
4.6 Informed Consent 26
4.6.1 Process of Consent 26
4.6.2 Consent Form 26
4.6.3 HIPAA 26
4.6.4 Revoking Consent 27
4.6.5 Costs to the Subject 27
4.6.6 Payment for Participation 27
4.7 Early Withdrawal of Subjects 27

4.7.1 Premature termination of study 27
4.7.2 When and How to Withdraw Subjects 28
4.7.3 Data Collection and Follow-up for Withdrawn Subjects 28
5 Study Procedures 28
5.1 Prior and Concomitant Therapy 28
5.2 Randomization and Blinding of Intervention 28
5.3 Established Standard of Care 29
5.4 Study Visits 29
5.4.1 Screening/Baseline: 29
The same procedures will be followed at both research sites (Madison and Milwaukee). 29
5.4.2 Intervention 32
5.4.3 Follow up: 34
5.4.4 Unscheduled: 34
5.4.5 Final Study Visit 34
6 Study Analysis 35
6.1 Sample Size Determination 35
6.2 Statistical Methods 35
6.3 Planned Interim Analysis: 35
7 Data Collection, Handling, and Record Keeping 36
7.1 Data Confidentiality 36
7.1.1 Confidentiality of Subject Records 38
7.2 Data Capture 38
7.2.1 Source Documents 38
7.2.2 Case Report Forms 39
7.3 Data Management 39
7.4 Data Monitoring 39
7.5 Records Retention 39
8 Assessment of Safety 40
8.1 Specifications of Safety Parameters 40
8.1.1 Definition of Adverse Events (AE) 40
8.1.2 Definition of Serious Adverse Events (SAE) 40
8.1.3 Definition of Unanticipated Problems (UP) 41
8.2 Classification of an Adverse Event 41
8.2.1 Severity of Event 41
8.2.2 Expectedness 41
8.3 Time Period and Frequency for Event Assessment and Follow-Up 42
8.4 Reporting procedures 42
8.4.1 Adverse Event (AE) Reporting 42
8.4.2 Serious Adverse Event (SAE) Reporting 42

8.4.3 Unanticipated Problem (UP) Reporting ... 43
8.4.4 Reporting of Pregnancy ... 43
8.5 Study Halting Rules ... 43
8.6 Safety Oversight ... 43
8.7 Unblinding Procedure ... 44
9 Study Monitoring, Auditing, and Inspecting ... 44
9.1 Medical Monitoring ... 44
9.1.1 Study Monitoring Plan ... 44
9.2 Protocol Deviations ... 44
9.2.1 Internal Data and Safety Monitoring Board ... 45
9.3 Auditing and Inspecting ... 45
9.4 Subject Compliance Monitoring ... 45
10 Ethical Considerations ... 45
11 Study Finances ... 46
11.1 Funding Source ... 46
11.2 Conflict of Interest ... 46
12 Publication Plan ... 46
13 References ... 46

1 Key Roles

See Appendix for the Delegation of Authority log.

Following is a list of all personnel in key roles:

Kara Hoppe, DO, Principal Investigator; Site Principal Investigator – UW Health; Madison, WI
University of Wisconsin-Madison,
University of Wisconsin (UW) School of Medicine & Public Health, Dept. of Obstetrics & Gynecology
McConnell Hall, 1010 Mound Street
Madison, WI 53715
Office: (608) 417-4224
khoppe2@wisc.edu

Lisa Sullivan-Vedder, MD, Site Principal Investigator – Aurora Health Care; Milwaukee, WI
Aurora Health Care,
Family Care Center
1020 N. 12th Street
Milwaukee, WI 53233
Office: (414) 219-5970
Lisa.Vedder@aurora.org

UW Department of Biostatistics and Medical Informatics
KyungMann Kim, PhD, Statistician
256a Warf Office Building
610 Walnut St
Madison, WI 53726
(608) 265-6380; (608) 263-1706
Kyungmann.kim@wisc.edu

UW Institute for Clinical and Translational Research (ICTR) Data Monitoring Committee (DMC)
University of Wisconsin School of Medicine and Public Health
2112 Health Sciences Learning Center
750 Highland Avenue
Madison, WI 53705
(608) 263-3804

Aurora Research Institute
960 N 12th Street, Suite 4120
Milwaukee, WI 53233
(414) 219-4763

Paula Einhorn, MD, MS
NIH/ National Heart, Lung, and Blood Institute Project Officer
Division of Cardiovascular Sciences
6701 Rockledge Drive, Room 10222
Bethesda, MD 20892
(301) 435-0563
einhorn@nhlbi.nih.gov

2 Background and Introduction

This document is a protocol for a human research study. This study is to be conducted according to NIH and Institutional research policies and procedures.

2.1 Background and Rationale

Uncontrolled hypertension in young adults is an enormous public health burden.[1,2] In the U.S., over 10 million 18-39 year-olds (1 in 5 men; 1 in 6 women) have hypertension,[3-6] increasing their risk of heart failure, stroke, and chronic kidney disease.[5,7-10] The medical costs of hypertension and its complications are $131 billion/year,[11,12] contributing to 1,000 deaths per day.[3,5,11,13-15] Young adults with hypertension have a higher lifetime risk for cardiovascular disease, especially with complicated hypertension (hypertension with diabetes or chronic kidney disease).[9,14-20] Hypertension control reduces morbidity, mortality, and future healthcare costs;[21-25] yet, only 35% of young adults with hypertension in the U.S. have achieved blood pressure control (blood pressure <140/90 mmHg).[26-29]

While hypertension self-management programs targeted towards adults ≥50 years old reduce blood pressure,[30-48] they primarily focus on medication titration.[49] In contrast, a trial of lifestyle modifications is commonly the initial hypertension treatment step, rather than a medication, among young adults.[50] Medication initiation is also not enough to achieve long-term blood pressure control in young adults. Even with medication adherence, systolic blood pressure continues to increase from younger to older age.[51] Therefore, young adults need additional hypertension self-management (home blood pressure monitoring and lifestyle modifications) to lower blood pressures and reduce the amount of medication they may need over a lifetime.[52-55]

The Institute of Medicine[56] highlighted the importance of self-management support to learn and effectively perform self-management.[30,34,36,57,58] In multiple studies among older adults, hypertension self-management is superior to office-based management.[30,34,36,57-59] However, in contrast to older adults, young adulthood is a time of frequent healthcare and vocational transitions, new life responsibilities, and less interest in health-related goals (*i.e.*, prevention of heart attack and stroke).[60,61] Thus, the content and method of delivery of hypertension self-management must be individualized to young adults and address barriers specific to this population.[62-64]

Our previous research highlighted the critical need to design hypertension programs targeted at young adults. An analysis of 48 months of data (Johnson HM, et al.), published in the *Journal of Hypertension,*[60] evaluated diagnosis rates of 4,023 young adults with regular primary care visits and ongoing elevated blood pressures, but without a hypertension diagnosis. Among adults (≥18 years) only ~59% of young adults (18-39 year-olds) received an initial hypertension diagnosis as compared to over 70% of ≥40 year-olds ($p<0.001$). After adjustment for patient comorbidities, sociodemographic, and provider characteristics, young adults had a 33% lower rate of receiving a hypertension diagnosis compared to ≥60 year-olds ($p=0.007$). In a separate study, we demonstrated that young adults with isolated systolic hypertension had a 50% slower diagnosis rate than young adults with isolated diastolic or combined systolic/diastolic hypertension ($p<0.001$).[65] Our publication in the *Journal of General Internal Medicine*[50] demonstrated similar disparities in hypertension treatment. Adjusted analysis demonstrated that young adults had a 44% lower rate of medication initiation (HR 0.56; 0.47-0.67, $p<0.001$) than ≥60 year-olds. Thus, many more young adults continued on with uncontrolled blood pressure and no medication.

We also published the first U.S. analysis describing electronic health record documentation of lifestyle education for young adults with incident (new) hypertension (Johnson HM, et al.).[66] Among 500 randomly selected 18-39 year-olds, only 55% had documented lifestyle education within one year of developing hypertension. Critical provider and patient barriers included limited time to manage multiple co-morbidities and clinic visit non-adherence (no-shows, not scheduling follow-up visits), respectively. Therefore, out-of-clinic self-management support is needed to overcome these barriers. Of note, there were not significant race/ethnicity differences despite the higher prevalence of hypertension among Black young adults.[67]

2.1.1 Development of MyHEART

To address the unmet need in hypertensive care for young adults, we developed MyHEART (My Hypertension Education And Reaching Target), a multi-component, theoretically-based intervention designed to achieve self-management among young adults with uncontrolled hypertension. MyHEART is a patient-centered program that uses evidence-based health behavior approaches to lower blood pressure. MyHEART differs from previous interventions by: **1)** targeting barriers identified by young adults in our preliminary research, **2)** tailoring the mode of delivery to preferences expressed by members of this age group, and **3)** individualizing action plans to participants' motivations and behavior goals. To design this intervention, we conducted focus groups of young adults with

hypertension (45% Black)[61] and one-on-one interviews of providers[68] in Madison (academic community), Milwaukee (urban), and Richland Center (rural), Wisconsin. Our diverse young adult respondents expressed a strong interest in blood pressure self-monitoring and increased communication with their healthcare team. They helped us recognize important hypertension education topics that were not addressed in current healthcare system handouts. Stakeholders identified barriers to hypertension self-management and hypertension control among young adults, justifying the need for this intervention, and proposed specific solutions to the identified barriers.

MyHEART is founded on the Self-Determination Theory (SDT), which is used to support chronic disease self-management and lifestyle modifications.[69-73] SDT has been used across races/ethnicities and with young adults.[74,75] To reduce the risk of negative sequelae from long-term hypertension, we need to not only initiate behaviors for blood pressure control, but also foster maintenance of new behaviors. SDT acknowledges that young adults are more likely to adopt and maintain health behaviors with: **1)** autonomous (internal) motivation, instead of external motivation (*i.e.*, external pressure), **2)** relatedness (*i.e.*, supportive healthcare interactions), and **3)** perceived self-competence (*i.e.*, perceived self-efficacy;[76,77] confidence in starting and maintaining behaviors to reach a goal).[69,72,74,78-89] The information gained by applying a theoretical framework is crucial to designing interventions and understanding the translation of interventions to other chronic diseases and populations.[90]

MyHEART incorporates important hypertension education components implemented by a health coach: **1)** telephone-based self-management counseling, **2)** home blood pressure monitoring, and **3)** young adult-focused hypertension education. These components are recommended by the Institute of Medicine[91] and the American Heart Association;[1] in previous studies, the largest effect sizes were achieved by multi-component interventions that incorporated both behavioral and educational strategies.[92,93] Health coaching provides informational and emotional support for chronic disease management.[94-96] Telephone interventions increase contacts between patients and their healthcare team.[97-100] MyHEART uses telephone as the primary mode of communication between participants and coaches, because young adults indicated a preference for this mode of delivery. Contrary to our hypothesis, our focus group respondents disliked text messaging and social media for health coach communication or self-management reminders;[61] young adults were concerned that peers would see the hypertension communications.

MyHEART directly addresses NHLBI's Strategic Priority 3.1.a to: "Develop and evaluate new approaches to implement proven preventive and lifestyle interventions." MyHEART takes guideline-recommended hypertension self-management tools and, in contrast to previous interventions, targets the delivery to young adults to increase the initiation and maintenance of behaviors to lower blood pressure.

2.2 Hypothesis

Aim 1. To evaluate the effect of MyHEART (home blood pressure monitor distribution and heath coaching) on clinical outcomes, the change in systolic and diastolic blood pressure (primary) and hypertension control (secondary) after 6 and 12 months, compared to usual clinical care. **We hypothesize** that MyHEART will significantly decrease systolic and diastolic blood pressures (clinic and 24-hour ambulatory) in young adults, compared to usual clinical care.

Aim 2. To evaluate the effect of MyHEART on hypertension self-management behavior (behavioral outcomes) at 6 and 12 months, compared to usual clinical care. **We hypothesize** that MyHEART will increase the frequency of home blood pressure monitoring and lifestyle modifications (increased physical activity, decreased sodium intake).

Aim 3. To examine whether MyHEART's effects on self-management behavior are mediated through variables of perceived competence, autonomy, motivation, and activation (mediation outcomes). Based on our pilot study and theoretical framework, **we hypothesize** that MyHEART's effects will be mediated through perceived competence, autonomy, internal motivation, and patient activation.

2.3 Study Agent

No investigational drugs, devices, or biologics will be used in this study.

2.4 Summary of Clinical Data

Established Recommendations for Lifestyle Modifications[101]

The Fifth Report of the Joint National Committee on the Detection, Evaluation and Treatment of High Blood Pressure (JNC V) and the Working Group Report on Primary Prevention of Hypertension recommended four lifestyle modifications to reduce blood pressure: **1)** reduced sodium intake, **2)** weight loss, **3)** reduced alcohol consumption, and **4)** increased physical activity.[102] Based on the results of the Dietary Approaches to Stop Hypertension (DASH) clinical trial,[13] the Sixth Report of the Joint National Committee also recommends a diet rich in fruits, vegetables, and low-fat dairy products, and reduced in saturated fat, total fat, and cholesterol.[103]

Reduced Sodium Intake

Inter-population and intra-population observational studies have documented a positive, direct relationship between sodium intake and blood pressure, and experimental studies confirm this relationship.[104-107] Furthermore, from a population perspective, the benefits of a reduced sodium diet significantly outweigh possible risks. Since most adult Americans consume well over the maximum recommended daily intake of 2,300 mg of sodium, virtually all Americans are candidates for reducing sodium intake. Trials have demonstrated that behavior change interventions can reduce daily intake by approximately 690-1150 mg.[108] Results tend to differ by race-ethnicity and to a lesser extent by gender, such that sodium reduction is less in African Americans than in European Americans and less in women than in men; the latter is largely explained by lower baseline intakes of sodium.[109] Additional analyses of data indicate a dose response relationship between sodium reduction and the extent of blood pressure reduction and hypertension control.[13] Trials have also documented that a reduced sodium intake, once achieved, tends to be well maintained.[110]

Weight Loss

A strong and persuasive body of evidence from both observational and experimental studies indicates that weight is positively (directly) associated with blood pressure and hypertension.[111] The relationship is present in both genders and in most ethnic-racial groups. The importance of this relationship is reinforced by the high and increasing prevalence of obesity in the United States.[112] Virtually every major trial that has examined the influence of weight loss on blood pressure has documented a substantial and significant relationship between change in weight and change in blood pressure. Reductions in blood pressure occur even before (and without) attainment of desirable body weight. In studies that aggregated results across weight loss trials, the average SBP/DBP reduction per kg of weight loss was 1.6/1.1 mmHg.

Regular Physical Activity

Evidence from observational studies and experimental studies suggests that increased physical activity can lower blood pressure. Numerous studies have found a negative correlation between habitual physical activity and the development of hypertension. An inverse relationship between physical activity and blood pressure has been observed in both sexes, all age groups, and in both African American and European Americans.[113,114] In addition to the observational evidence, more than 30 experimental studies have evaluated the impact of physical activity on blood pressure.[115,116] Most of these studies used aerobic training protocols at vigorous intensities (*i.e.*, 60% maximal oxygen uptake or 70% maximal heart rate or greater). Fewer trials have evaluated lower intensity of exercise for blood pressure effects. Moderate-intensity activity has been shown to decrease blood pressure to an extent similar to, if not greater than, higher-intensity exercise in normotensive and hypertensive individuals.[117] The entirety of these studies indicates that regular, moderate to vigorous physical activity lowers blood pressure by 10/8 mmHg in hypertensives and 2/3 mmHg in normotensives. Policy-making bodies deem the evidence sufficient to advocate regular aerobic physical activity as a means to reduce blood pressure.[21,103,117]

Limitation of Alcohol Intake

The relationship between high alcohol intake (typically three or more drinks per day) and elevated blood pressure has been reported in a large number of observational studies.[118,119] In the Prevention and Treatment of Hypertension Study (PATHS), a reduction in alcohol intake among moderate drinkers also reduced blood pressure

to a small, albeit nonsignificant, extent.[120] A few trials have also demonstrated that reductions in alcohol intake among heavy drinkers can lower blood pressure in normotensive and hypertensive men.[121,122]

Dietary Patterns and Blood Pressure

Results from the Dietary Approaches to Stop Hypertension (DASH) clinical trial, in conjunction with previous studies of vegetarian diets, provide strong and persuasive evidence that modification of dietary patterns can have a profound influence on blood pressure.[107]

2.5 Potential Risk and Benefits to Subjects

2.5.1 Known Potential Risks

Blood Pressure Cuffs: There is a small immediate risk that there may be minor discomfort or bruising at the site of the upper arm automatic blood pressure cuff. To avoid this, patients will receive the appropriately sized cuff based on their upper arm circumference. In addition, the 24-hour ambulatory monitor will be started prior to leaving the research clinic to obtain at least two blood pressures to ensure comfort. Participants will also review information on how to contact the research staff in case additional questions, concerns, or new discomfort arise.

Muscle/Joint Discomfort: There is a small immediate risk that participants may experience new muscle and/or joint discomfort after starting a new exercise program. However, they will be instructed to consult their provider before starting a new exercise program.

Questionnaires: Multiple interviewer-administered and computer-administered questionnaires and surveys will be completed at each visit. We expect this will pose very minimal risk of physical or psychological discomfort.[123]

Breach of Confidentiality: The major potential social and psychological risk to participants is loss of confidentiality. As our standard operating procedure, we have policies and procedures in place to protect the confidentiality and security of subject data, see below in “Protection Against Risks”. Young adults may be concerned about social stigma associated with high blood pressure; therefore, immediate and/or long-range psychological risk is possible. However, to minimize this risk as much as possible we will protect the confidentiality of participants with a unique subject identifier number as outlined below in “Protection Against Risks”. We anticipate no additional psychological or legal consequence from study participation. Participants and their insurance plans will not be charged for any of the visits, telephone contacts, or 24-hour ambulatory blood pressure monitoring.

Reproductive risk: No reproductive risks are expected from this trial. This study does not start, change, or alter medications in any way. Patients are excluded if pregnant and if planning to become pregnant.

Hyperkalemia: The DASH diet may include a higher intake of potassium (due to a high content of daily fruits and vegetables).

Hypoglycemia: During or immediately after exercise, there is a risk of hypoglycemia for subjects with diabetes mellitus.

Clinically Significant Blood Pressure Findings: Clinically relevant results of blood pressure measurements will be released to all subjects, as well as to their specific provider if they opt to give the provider's information on the written consent form. Only clinically significant findings will be disclosed: research clinic blood pressure >/= 160/100 mmHg (during any research clinic visit), right/left arm blood pressure differential of >/= 20 mmHg (Visit 1), and normal mean 24-hour ambulatory blood pressure (Visit 2). These clinical significant findings will be noted immediately during the research clinic visit, and the research examiner will notify the subject verbally, complete the incidental finding form, and provide a completed copy to the subject during the same visit. A copy of the incidental finding form will be forwarded to the primary care provider (via fax) on the same day.

The protocol appendix has a table explaining recommended primary care follow-up based upon the research clinic blood pressures (for all subjects). This is separate from the incidental finding process. Since young adults will be arriving with elevated blood pressures, they should also continue to have routine clinical care per hypertension guideline recommendations. This recommendation will be given by the research examiner on the same day as the visit.

The same primary care follow-up (protocol appendix) will also be recommended based upon home blood pressure readings (intervention arm only) by the health coach before the end of that call. However, instead of the participant being "escorted" to the emergency department, they will be instructed to call 911.

Clinically significant findings could result in anxiety and psychological discomfort. Financial risks to clinically significant findings could mean that subjects and their insurance would have to pay for follow-up procedures or visits. False-positives are possible (*i.e.*, a clinically concerning blood pressure measurement in our research clinic), that is "white coat hypertension", not high blood pressure.

An additional risk is social stigma associated with high blood pressure and associated immediate and/or long-range psychological risks.

2.5.2 Alternative to Study Participation

The alternative to study participation for all subjects is to continue to receive blood pressure care from their healthcare providers, which would include blood pressure checks, blood pressure lifestyle counseling/education, antihypertensive medication initiation and/or titration, and following their provider's treatment recommendations. This may also include referrals to specialty care (example: dieticians). Participants may choose to not participate in the study and at any time may discontinue their participation in the study.

Rationale for the necessity of exposing human participants to such risks: It is expected that this study will pose minimal risks to participants and the intervention outlined in this proposal (24-hour, clinic, home blood pressure monitoring, behavior change) are the same clinical interventions that can be prescribed during usual clinical care.

Why the value of the information to be gained outweighs the risks involved: Young adults with uncontrolled hypertension are at increased risks for premature heart failure, stroke, and chronic kidney disease. Therefore, the value of the information gained from this study far outweighs the risks involved.

2.5.3 Protection Against Risks

As indicated above, we expect minimum risk to participants. The primary pre-defined serious adverse events related to this study includes hypoglycemia among patients with diabetes mellitus starting a new exercise program. Subjects with any form of diabetes will be instructed to talk with their diabetes care team about adjusting their diabetes medication before starting a new exercise regimen and to discuss ways to treat hypoglycemia. Additionally, these recommendations will be reinforced with a handout from our study team about risk of hypoglycemia, signs, symptoms, and treatment of hypoglycemia and how to decrease their risk of hypoglycemia.

Patients with advanced chronic kidney disease (on dialysis or seeing a nephrologist) will be excluded to avoid serious adverse events with the Dietary Approaches to Stop Hypertension (DASH) diet, which may cause hyperkalemia. Once enrolled, study protocols will monitor and assess the presence of adverse events (AEs) and serious adverse events (SAEs) at all follow-up contacts.

Should a serious adverse event or adverse event be identified, it will be immediately reported as outlined later in this protocol. Should excessive risk to study participants be determined, the study will be stopped and all participants notified in a manner appropriate to the nature of the risk.

Emails will be used for sending 1) research clinic visit appointment reminders for all subjects (day, time, location, directions to UWHC or UWSMPH) and 2) handouts on blood pressure topics to reinforce the health coach phone call (intervention arm only - if this method was selected by the subject over postal mail). Each email will contain the minimum amount of information needed. Emails will be sent individually, with no cc or bcc. No group emails or lists will be used for this study. Emails will not contain sensitive protected health information (PHI). Per the University of Wisconsin HIPAA policy (www.hipaa.wisc.edu), the subject of the email will be “Documents for your UW Study”.

Blood Pressure Cuffs: To minimize risks, patients will receive the appropriately sized cuff based on their upper arm circumference. In addition, the 24-hour ambulatory monitor will be started prior to leaving the research clinic to obtain at least two blood pressures to ensure comfort. Participants will also review information on how to contact the research staff in case additional questions, concerns, or new discomfort arise.

Muscle/joint discomfort: Participants will be instructed to consult their provider before starting a new exercise program.

Questionnaires/surveys: Participants will be informed that they can decline to answer any questions.

Health coach fidelity audio recordings:

- For Health Coach Calls Performed in Madison, WI: The audio digital recordings will be kept in a locked file cabinet in the PI’s office located in the Health Innovation Program (HIP) at 800 University Bay Drive Suite 210, Madison, WI 53705. The audio files will be electronically transmitted by the MyHEART research staff to a UW Box file storage (approved by Richard Konopacki, UW SMPH; May 2017) accessible to Dr. Diane Lauver (lead researcher for this analysis) and a School of Nursing research student (a personnel change IRB application will be submitted once determined). The original and transmitted digital audio copies will be deleted/destroyed at the end of this study upon publication of the fidelity data.
- For Health Coach Calls Performed in Milwaukee, WI: The audio digital recordings will be kept in a locked file cabinet in an Aurora Health Care research office. Encrypted audio files will be emailed from a password protected research computer at Aurora Health Care to the study PI and study research coordinator, using Aurora Health Care and UW-Madison/Department of Medicine emails. The audio files will then be saved on the HIP network and maintained separately from other study documents in a password-protected database on password-protected media. The audio files will then be electronically transmitted by the MyHEART research staff to a UW Box file storage (approved by Richard Konopacki, UW SMPH; May 2017) accessible to Dr. Diane Lauver (lead researcher for this analysis) and a School of Nursing research student (a personnel change IRB application will be submitted once determined).

All original and transmitted digital audio copies will be deleted/destroyed at both research sites at the end of this study upon publication of the fidelity data.

Breach of Confidentiality - Identifying information: Subject confidentiality will be protected with the coded patient IDs whenever possible. Linking information will be maintained separately from other study documents in a password-protected database on password-protected media.

- Madison site: When handwritten or printed documents are required that include PHI or non-PHI with identifiers, the paper copies will be secured in a locked file cabinet within a locked office within the HIP Suite at 800 University Bay Drive (Suite 210) or within the UW Hospital and Clinics, Division of Cardiovascular Medicine (H4/5). Access to the paper records will be restricted to the PI and project personnel. As of May 2022, all paper records will be stored at the State of WI long term storage.
- Aurora site: When handwritten or printed documents are required that include PHI or non-PHI with identifiers, the paper copies will be secured in a locked file cabinet within a locked office within a Aurora Health Care research office, with oversight by the site PI. Access to the paper records will be restricted to the PI, site PI, and project personnel.

Analysis Data: Direct identifiers are removed by HIP and Aurora Programmers prior to data delivery to minimize the risk of loss of confidentiality. The data is labeled with a pseudo-identifier that is not derived from a patient identifying number. Analysis data is stored on a secure server under the control of the Health Innovation Program and access to study data is limited to persons who are approved to access it. Datasets for day-to-day analysis will be limited datasets (no direct identifiers; include zip codes and dates only; labeled with a pseudoidentifier). Details of data security protections are found in the "Privacy/confidentiality" section. Analysis data are never stored on paper. No sensitive information will be included in the analysis dataset. Encrypted Aurora Health Care data sets with coded patient IDs will be transferred to HIP and stored separately on the HIP network from data sets with linking information.

As of May 2022, all data will be transferred from HIP and stored on the UW Department of Obstetrics and Gynecology server. The transfer will proceed as follows:

HIP will copy and save files to a HIPAA compliant, FIPS140-2, encrypted disk. This disk has been approved by UW Cybersecurity as an acceptable media source to store PHI. In fact, if this disk were to be lost or stolen, only the business unit leadership would need to be notified (to replace the disk); UW Cybersecurity would not consider the loss a reportable event. The hard drive meets the security and compliance standards needed to store PHI. The hard drive will be stored in a locked cabinet in a locked office at HIP until MyHEART could bring it to Ob/Gyn.

No individual PHI will be released in presentation or publication. Only aggregate statistical output representing groups of subjects will be released or removed from the secure HIP or UW Ob/Gyn department servers.

The Wisconsin Network for Health Research (WiNHR) of UW-Madison's Clinical and Translational Science Award (CTSA) will oversee regulatory processes for this study, with the principal Investigator, site principal investigators, and the ICTR Data Monitoring Committee (DMC). The WiNHR staff have developed plans for assuring data accuracy and protocol compliance that will include biannual reviews with all research staff and WiNHR research coordinators. All study data will be documented and monitored in ICTR's REDCap. Such plans will include data verification and protocol compliance checks. The Principal Investigator will also be responsible for ensuring that the data for the project are securely stored, that storage is in compliance with University and federal regulations, and that no unauthorized persons have access (electronic or physical) to any participant-identifiable data. All HIPAA regulations and guidelines will be followed, and all study staff must complete approved human subjects and HIPAA training programs.

2.5.4 Adverse Event Reporting

MyHEART's data safety monitoring plan requires that investigators (PI, site-PIs) notify the NIH, DMC, and the University of Wisconsin IRB in a timely manner (consistent with IRB and NIH policies) of the occurrence of any SAE or any AE which is severe, unexpected, and possibly related to the protocol. This study does not involve pharmaceutical agents. Examples of SAE would be untoward medical or intervention occurrences that result in death, are life-threatening, require hospitalization or prolonging of existing hospitalization, or create persistent or significant disability/incapacity. Unanticipated AEs would include less serious problems that merit reporting because they are severe, unexpected, and possibly related to study participation. Any SAE will be queried and reported even if it appears that the serious adverse event is unrelated to intervention participation. The Principal Investigators will also be responsible for the accurate documentation, investigation, and follow-up of all study-related adverse events.

Adverse event assessment, recording, reporting, and investigation will be accomplished through staff training, structured/standardized assessments of untoward occurrences/events, and regular monitoring by site principal investigators using REDCap. The Principal Investigators have ultimate responsibility for ensuring that SAEs are detected and reported in a timely manner. Additionally, the IRB will receive an annual report of all SAEs and AEs meeting the criteria listed above.

Important medical events are those that may not be immediately life threatening, but are clearly of major clinical significance. They may jeopardize the subject, and may require intervention to prevent one of the other serious outcomes noted above. For example, treatment of bronchospasm in an emergency department would typically be considered serious. The primary pre-defined serious adverse events related to this study include hypoglycemia among patients with diabetes mellitus starting a new exercise program. All adverse events that do not meet any of the criteria for 'serious' should be regarded as *non-serious adverse events*. Throughout the study, any new clinically significant findings/abnormalities that meet the definition of an adverse event will also be recorded and documented as an adverse event.

Adverse Event Reporting Period: Adverse events must be reported once the subject undergoes any study procedures, and adverse events must be reported during the entire active study period and for 30 days following the last administration of study treatment.

Withdrawal of subjects: Any subject who experiences an adverse event or serious adverse event will be immediately withdrawn from the study. All subjects have the option to withdraw from the study at any time. Subjects will also be immediately withdrawn from the study if they report being pregnant, planning to become pregnancy, or develop a medical condition (even if unrelated from this study) that prevents them from ongoing participation in the study.

Post-study Adverse Event: All unresolved adverse events will be followed by the investigator until the events are resolved, the subject is lost to follow-up, or the adverse event is otherwise explained. At the last scheduled visit, the investigator will instruct each subject to report any subsequent event(s) that the subject, or the subject's personal physician, believes might be related to participation in this study. The investigator should notify the study sponsor,

DMC, and IRB of any serious adverse event or death occurring up to 30 days after the subject has discontinued or terminated study participation that may be related to this study.

Hospitalization, Prolonged Hospitalization, or Surgery: Any adverse event that results in hospitalization or prolonged hospitalization will be documented and reported as a serious adverse event unless specifically instructed otherwise in this protocol. Any condition responsible for surgery should be documented as an adverse event if the condition meets the criteria for an adverse event.

Neither the condition, hospitalization, prolonged hospitalization, nor surgery are reported as an adverse event in the following circumstance: Hospitalization or prolonged hospitalization for diagnostic or elective surgical procedures for a preexisting condition. Surgery should ***not*** be reported as an outcome of an adverse event if the purpose of the surgery was elective or diagnostic and the outcome was uneventful.

Referral for treatment, counseling, or other necessary follow-up. Participants who report excess alcohol use, psychiatric illness (new or chronic), and/or willingness to quit smoking will be referred to their primary care provider for necessary follow-up. Subjects will also receive written handouts about alcohol/tobacco cessation resources (*e.g.*, Wisconsin Tobacco Quit Line). Charges for treatment, counseling, and/or follow-up will be the sole responsibility of the subject.

2.5.5 Potential Benefits to the Subjects

We do not expect any direct benefits to the study subjects randomized to the control group. We believe that those in the MyHEART intervention group will demonstrate lower systolic and diastolic blood pressures, but this cannot be guaranteed. If our results are positive, we are hopeful that our and other healthcare systems will use our results in planning interventions to improve the hypertension treatment for young adults. The minimal risks to the subjects are very reasonable in relation to the potential benefits to future young adults with hypertension if we find evidence that the MyHEART intervention is effective.

2.5.6 Risk Minimization

No procedures, situations, or materials are expected to be hazardous. Except for the questionnaires, the remaining study activities can be provided during routine clinical care. Introduction of the intervention will not increase the risk of the procedures involved in standard of care.

Study staff interacting with enrolled participants will be blinded to the randomization to decrease risk of potential biases.

3 Study Design and Endpoints

3.1 General Design

Figure: Eligibility, Enrollment, Randomization, Follow-up and Analysis

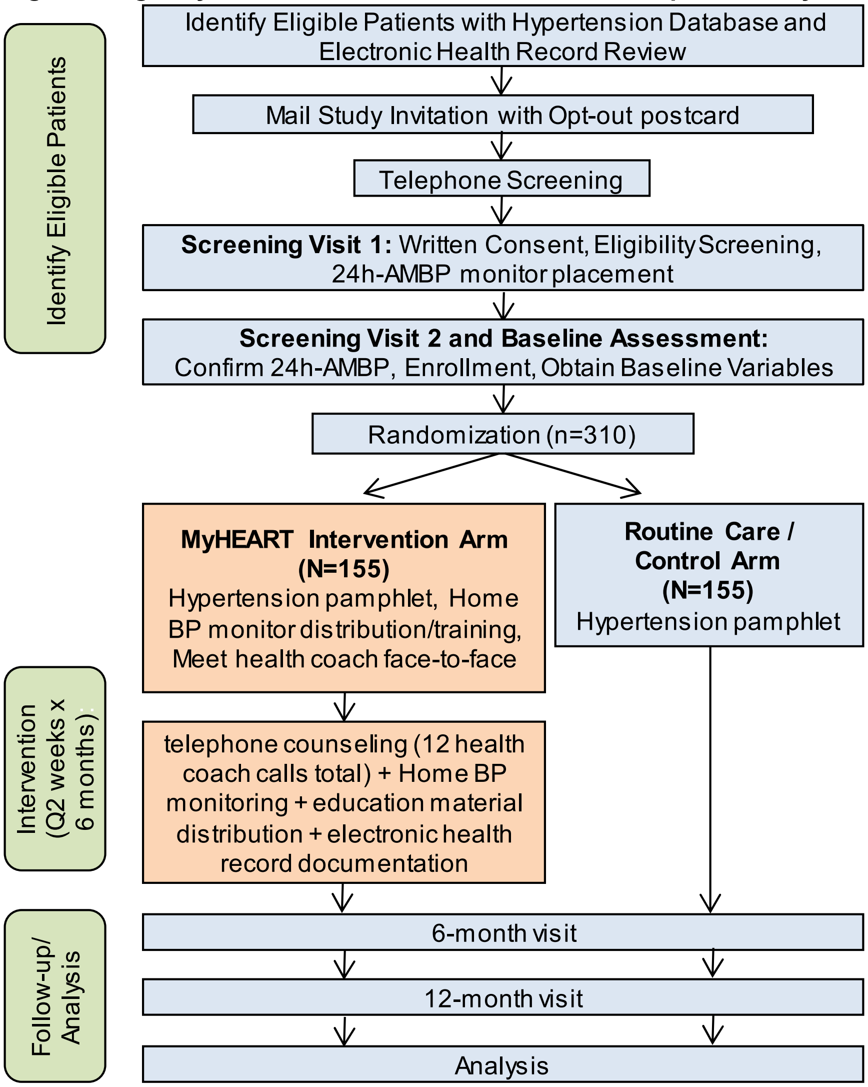

MyHEART is a randomized, controlled clinical trial, across two large IRB-approved healthcare systems that will examine the impact on several health outcomes of a self-monitoring intervention designed to decrease blood pressure in young adults with uncontrolled hypertension.

The study cohort will include 340 adults aged 18-39 years with documented hypertension. After screening for eligibility, participants will be randomly assigned to either the MyHEART intervention or usual (routine) care. In addition to their general medical care from the participants' own healthcare providers, those assigned to the MyHEART intervention will receive a hypertension education packet, a home blood pressure monitor, and 12 phone calls with a health coach to promote self-management of blood pressure.

The expected duration of subject participation, for all subjects, is 12 months.

3.1.1 Primary Study Endpoints

The co-primary clinical outcome is a significant, clinically important change in systolic and diastolic blood pressure at 6 and 12 months.[21,36] The 6-month outcome assesses the end of the 6-month MyHEART intervention.[43] The 12-month outcome (*i.e.*, 6 months post-intervention)[58] assesses maintenance of blood pressure and sustainability of behavior change after study completion. In contrast to the baseline assessment, follow-up questionnaires and physiologic measurements will be conducted, obtained, and documented during the first visit of the 6- and 12-month visits to decrease missing data.

3.1.2 Secondary Study Endpoints

- Hypertension Control: Percentage of participants that achieve hypertension control at 6 months. Hypertension control will be defined using ambulatory blood pressures as the gold standard (<130/80 mmHg); otherwise a clinic blood pressure of <140/90 mmHg will be used.
- Change in hypertension self-management behavior at 6 and 12 months compared to usual care
    - Dietary Changes (Automated Self-Administered 24-hour Dietary Assessment)
    - Change in physical activity (Godin Physical Activity Questionnaire)
    - Home Blood Pressure Monitoring Frequency[124]
    - Change in weight (kg)
    - Change in body mass index (BMI, kg/m^2)
- Change in perceived competence, autonomy, and motivation
    - Perceived Competence – Perceived Competence Scales (PCS)
    - Perceived autonomy/support form medical team - Health-Care Climate Questionnaire (HCCQ)
    - The Degree to which a person's motivation for a behavior is autonomous or self-determined: Treatment Self-Regulation Questionnaires (TSRQ)
    - Health Coach Fidelity

3.1.3 Primary Safety Endpoints

- Number and type of adverse events
- Number and type of serious adverse events
- Subject Withdrawal rate and reason

4 Study Subjects – Enrollment and Withdrawal

See Appendix for accrual expectations by research site

The recruitment and screening protocol will be the same at both sites. The accrual goal is 340 participants total (n=170 per arm) over 25 months of recruitment. Participants will be randomized to control or intervention arms at each site.

4.1 Subject Population

The age range of the subjects is 18-39 years. The young adult age range is based upon the hypertension guidelines for children and adolescents which applies to <18 year-olds.[125] The National Health and Nutrition Examination Survey (NHANES) data (as reported by the American Heart Association[67] and the Center for Disease Control[3]) limits young adults to <40 years old. Young adults were selected for this research since they have the lowest hypertension control rates compared to middle aged and older adults and lack effective hypertension interventions.

There are no enrollment restrictions based upon race or ethnic origins. We will target recruitment by minority status to ensure that we achieve our expected enrollment numbers. Nationally, the prevalence of hypertension among young adults is greater in young males and Blacks. We expect a higher recruitment of minority patients from an IRB-approved urban site based on the racial/ethnic distribution of patients. Women who are or become pregnant will be excluded to ensure we do not include patients with pregnancy-induced hypertension.

The information provided in the Targeted/Planned Enrollment Table represents the gender, ethnic, and racial make-up from a sample of potentially eligible subjects in the electronic health record:

UW Health: Sex: 62% male; Ethnicity: 5% Hispanic/Latino; the racial make-up is 82% white, 13% black, 2% Asian, 0.3% American Indian or Alaska Native, and 3% “more than one race”.

4.2 Inclusion Criteria

Inclusion Criteria
1. Willing and capable of giving written informed consent
2. Willing to comply with all study procedures and be available for the duration of the study
3. Males and females ages 18-39 years old at the start of the study (inclusive)
4. A single ICD-10 diagnosis code R03.0 (elevated blood pressure reading, without diagnosis of hypertension) in the last 24 months, OR, a minimum of two hypertension ICD-10 coded visits with a provider (MD, DO, PA, NP) on different dates in the last 24 months, with at least one code in the past 18 months
5. Medically homed at UW Health or Aurora Health Care

The inclusion criteria are designed to ensure that patients who have a single visit to a clinic but seek primary care permanently elsewhere are not included in our study.[126] The patient has to be managed at the same healthcare system prior to and during the study.

4.3 Exclusion Criteria

Exclusion Criteria
1. History of medically determined Congestive Heart Failure
2. Unable to provide informed consent (*i.e.,* activated healthcare power of attorney)
3. Unable or unwilling to travel to local clinic for research visits
4. Currently residing in a skilled nursing facility
5. Diagnosed with sickle cell anemia or cystic fibrosis
6. Diagnosed with stroke, myocardial infarction, and/or coronary artery revascularization in the past 2 years
7. Syncope while exercising or doing strenuous activity within past 12 months
8. Currently prescribed warfarin, novel oral anticoagulant, or insulin
9. Planned organ transplant or prior transplant in the past 5 years
10. Chemotherapy or radiation therapy within 6 the past months
11. Severely impaired hearing, vision, or speech, as determined by study staff responsible for enrollment
12. Current participation or planning to participate in another clinical trial in the next 12 months
13. Pregnant or planning to become pregnant in the next 12 months
14. Planning to leave the geographic area in the next 6 months
15. Health condition that will limit both increasing physical activity and changing diet
16. Illegal drug use (other than marijuana) in the past 30 days

17. Unable to read or communicate in English
18. Currently on dialysis or seeing a Nephrologist
19. Unaware or denies history of high blood pressure or hypertension
20. Between-arm blood pressure difference $\geq$20 mmHg
21. White Coat Hypertension (24-hour ambulatory monitoring)
22. Inability to comply with or complete the protocol or other reasons at the discretion of the principal and site investigators
23. Prisoners

This list of medical conditions or conflicting medications was selected to exclude patients who might experience harm from participation. Patients with advanced chronic kidney disease (on dialysis or seeing a nephrologist) or congestive heart failure are excluded due to stringent dietary restrictions. Illegal drug use (except marijuana) in the past 30 days will result in ineligibility; illegal drug use will not be recorded.

4.4 Subject Screening for Recruitment

4.4.1 Subject Identification

There will not be differences in recruitment methods between sites or by potential subject group (*e.g.*, intervention versus a control group).

Subject recruitment, screening, and enrollment between IRB approved sites will be coordinated by the Wisconsin Network for Health Research (WiNHR) of UW-Madison's Clinical and Translational Science Award (CTSA). Each IRB approved site will identify potentially eligible patients using the Wisconsin Collaborative for Healthcare Quality hypertension registry data within respective electronic health record systems. Each IRB approved site will have an electronic health record with a Wisconsin Collaborative for Healthcare Quality (WCHQ) hypertension registry that will be used to identify potentially eligible patients.[127] The electronic health record will also be used to identify individuals with the ICD-10 diagnosis code R03.0 (elevated blood pressure reading, without diagnosis of hypertension). The inclusion criteria will be entered by the data analysts at each healthcare system, which will generate a list of potentially eligible patients.[50,60]

The principal investigator and Milwaukee site investigator will work with programmers to determine who is potentially eligible based on the EHR database. The project manager and student research coordinators will be involved with mailing introductory packets, telephone screening, and inviting them for their first screening visits.

At the screening visit, research coordinators will lead eligibility assessment, informed consent, and enrollment. The PI will oversee all aspects of this study.

4.4.2 Recruitment and Retention Strategies

See appendix for expected numbers of women and minorities to be recruited.

The site coordinators will mail an introductory research packet to patients who have been identified by each electronic health record system as potentially eligible, based upon the WCHQ or ICD 10 diagnosis code R03.0 criteria described above. The packet will include an introductory letter, a flyer summarizing the MyHEART research project, a magnet, bookmark and a pre-paid opt-out postcard. The packet will only be mailed once to the home address listed within the electronic health record system. If a participant contacts us independently and requests a packet, it will be mailed to the address that is provided by them. If an opt-out response is not received after 2 weeks, a research staff member will call patients to perform telephone screening to assess eligibility based on inclusion and exclusion criteria; research staff at UW_Madison will be calling all Madison participants and research staff at Aurora Health Care will be calling all Milwaukee participants (telephone scripts for each site are uploaded to ARROW). The telephone screening for Aurora Health Care participants will also include a question to obtain verbal authorization that the participants give verbal permission for Aurora Health Care research staff to send their

information to the University of Wisconsin-Madison research staff for data entry. At the completion of the telephone screening, the patient will be subsequently invited for a face-to-face visit at the research clinic within their medically homed region (Madison or Milwaukee). We will recruit a total of 340 participants over 25 months. We anticipate an accrual rate of 12-13 eligible participants per month. We estimate having to screen approximately 930 potentially eligible participants.

We will also try to recruit by collaborating with primary care providers to review weekly panels and posting announcements within primary care clinics (upon approval from clinic managers and/or clinic directors).[128] Primary care providers can also invite patients to the study. Healthcare providers will be able to directly ask patients about participating in this study. The PI will ask for permission from the provider to review their clinic schedule to identify potential subjects. The providers will be asked to tell potential subjects to contact the research team by telephone. Providers will not be asked to collect potential subjects' contact information or provide it to study team members.

Other methods include newspaper, radio, TV, newsletters, and social media avenues and advertising to the general public via flyers and community boards. We will publicize the research study on the MyHEART website: myheartmychoice.org (a hypertension education website developed by the PI with funding from ICTR). The information on the website will be limited to what is usually contained in a ClinicalTrials.gov description [purpose, eligibility criteria, who to contact].

To enhance participant retention, we will contact patients with visit reminders via one email and one phone call.

4.5 Vulnerable Populations

TABLE: Vulnerable populations included and excluded from this study:

Include	Exclude	Vulnerable Population Type
	X	Adults unable to consent
	X	Individuals who are not yet adults (*e.g.*, infants, children, teenagers)
	X	Wards of the State (*e.g.*, foster children)
	X	Pregnant women
	X	Prisoners

No vulnerable populations will be enrolled. Children <18 years of age are not eligible to participate in the trial. Although hypertension in children is a critical issue, children will not be included for three reasons: 1) the diagnosis and evaluation of high blood pressure in children and adolescents have different clinical guidelines than adults and are based on the 95th percentile for sex, age, and height,[125] 2) the home and ambulatory blood pressure monitors budgeted for this proposal will not be appropriately sized for children, and 3) the reading level of the educational material was not targeted to children. A separate proposal is needed to study this population. Pregnant women are excluded because the medication management of hypertension is different among pregnant women and there may be greater risk than benefit with starting a new exercise program or making dietary changes among some pregnant women. Prisoners will be excluded as per the Office for Human Research Protections (OHRP) guidelines; in addition, prisoners do not have flexibility in dietary and exercise options, as does the general population.

4.5.1 Subject Capacity

All subjects will have the capacity to give written informed consent.

4.5.2 Subject/Representative Comprehension

To ensure comprehension, after hearing and reading about the study, the subject will be asked to summarize the study and the requested activities for participation in the study. The investigator will then ask the subject if anything could be clarified before written/signed consent is obtained.

Decisionally impaired adults are excluded from this study.

4.6 Informed Consent

The PI will be responsible for ensuring that valid consent is obtained and documented for all subjects unless the IRB waives the requirement for documentation of informed consent for all or part of the study.

The activities involved to review the EHR for potentially eligible subjects are considered “preparatory to research” and will be conducted by administrators of the WCHQ database and programmers for each respective healthcare system. If an opt-out card is not returned, then individuals will be contacted directly by the study team for a recruitment telephone call.

During the recruitment screening phone call for Aurora Health Care participants, individuals will be asked to give verbal permission for Aurora Health Care research staff to pass along their information to the University of Wisconsin- Madison research staff for data entry (an “altered authorization”).

For individuals who express interest in participating during the telephone call and who potentially qualify, screening visit 1 will be scheduled. Written informed consent will be obtained at the start of screening visit 1, which will include authorization for additional screening/eligibility assessment (*e.g.*, 24-hour ambulatory blood pressure monitoring) and gathering data, including information from the patient’s electronic health record and study procedures after enrollment, if eligible. Signed informed consent will be obtained upon the participants arriving for Visit 1 (prior to initiating data collection or physical contact for the visit). The consent process will consist of a detailed verbal description of the study including its risks, potential benefits, and requirements. Potentially eligible participants will be given an IRB-approved/stamped informed consent form with all required elements and factual correctness to read, then ample time to read and reflect on participating. If the individual requests, they will be given additional time to consider participation, including re-scheduling the screening visit. Before obtaining written consent, each individual will be allowed to ask questions until a decision can be made by the individual. When ready, participants will be required to sign the consent form. Study audits by the PI and WiNHR will include review of consent documents for signatures and use of the IRB date-stamped form to ensure compliance.

4.6.1 Process of Consent

Members of the research team including research coordinators and research assistants will be responsible for obtaining consent. The written/signed consent (original copy) will be stored in a locked file cabinet, in a locked office, on site at each research office. Electronically signed copies will be stored on the password protected research network, locked to all non-study staff.

The process of informed consent will be structured to be conducive to rational and thoughtful decision making by the subject – including time for discussion, questions, and the ability to reschedule if additional time for consideration is needed. Legally authorized representatives will not be allowed to sign the consent; patients who require legally authorized representatives are excluded from the study.

Auditors, Witnesses, and translators will not be part of the consent process. Non-English speakers are excluded from the study. Additionally, patients who are unable to read or write in English (*i.e.*, illiterate) are excluded.

4.6.2 Consent Form

See Appendix for Consent Form

A copy will be given to the individual that signed the form, on the same day the individual signs the form.

4.6.3 HIPAA

See Appendix for HIPAA Form

Information about study subjects will be kept confidential and managed according to the requirements of the Health Insurance Portability and Accountability Act of 1996 (HIPAA).

Signed HIPAA authorization will be obtained upon the participants arriving for Visit 1 (prior to initiating data collection for the visit) after reviewing the Consent form. The same process (including extended time) will be given to the individual to complete the HIPAA form. A copy will be given to the individual that signed the form, on the same day the individual signs the form.

4.6.4 Revoking Consent

After study enrollment, in the event that a subject revokes authorization to collect or use PHI, the investigator, by regulation, retains the ability to use all information collected prior to the revocation of subject authorization.

4.6.5 Costs to the Subject

Subjects will not have to pay for study procedures (such as home blood pressure monitors, 24-hour monitoring). Subjects who are randomized to receive a home blood pressure monitor will be able to keep the monitor after study completion. The patient will not be billed by the healthcare system or their health insurance company for any costs related to a study procedure.

Subjects will be responsible for any costs related to their blood pressure follow-up as directed by their healthcare team, such as clinic visits and blood pressure medication, including all out-of-pocket costs.

4.6.6 Payment for Participation

Payments to participants will be provided as the study progresses; subjects do not have to complete the entire study to receive a payment.

All intervention and usual care participants will receive a remuneration up to $170 for study completion. Each “face-to-face visit” will actually be two consecutive visits (screening/baseline [visits 1, 2], 6 month [visits 3, 4], and 12 month [visits 5, 6]). They will receive a total of $50 for each completed face-to-face visit assessment. The payment difference is in relation to the participant’s time commitment for that visit.

Screening Visit: Visit 1: $20

Screening/Baseline Visit: Visit 2: $30

6-month Visits: Visit 3: $30 Visit 4: $20

12-month Visits: Visit 5: $30 Visit 6: $20

If they arrive for Screening Visit 1 and are unable to complete the visit due to exclusion, they will receive $10 for their screening participation.

An additional $20 cash gift card will be provided to all participants at Visit 4 (6-month visit) to offset study-related cell phone/telephone charges.

The remuneration will acknowledge their time and study participation, not behavior change. Primary care providers and clinics will not receive an honorarium. Additional payment will not be available to subjects for travel or other costs (example: childcare) that are incurred.

4.7 Early Withdrawal of Subjects

4.7.1 Premature termination of study

This study may be temporarily suspended or prematurely terminated if there is sufficient reasonable cause. Written notification, documenting the reason for study suspension or termination, will be provided by the suspending or terminating party to all site investigators, the NIH/NHLBI, DSMB (*i.e.*, ICTR DMC), and IRB. If the study is prematurely terminated or suspended, the PI will promptly inform the IRB and will provide the reason(s) for the termination or suspension.

Circumstances that may warrant termination or suspension include, but are not limited to:

- Determination of unexpected, significant, or unacceptable risk to participants
- Demonstration of efficacy that would warrant stopping
- Insufficient compliance to protocol requirements by research study personnel

The study may resume once concerns about safety and protocol compliance are addressed and satisfy the IRB and/or the NIH. All participant data that was collected prior to study termination will be analyzed and continue to be handled/stored per this protocol.

4.7.2 When and How to Withdraw Subjects

Participants will be educated on the consent form that they are able to withdraw at any time without adverse effects to receiving healthcare services.

Women that are currently pregnant or plan to become pregnant during enrollment screening will be excluded from study participation. If a participant becomes pregnant during the study, she is excluded immediately from further participation in all study activities. The presence/absence of birth control is not an inclusion or exclusion criteria. Pregnancy testing is not part of the study protocol. Once enrolled in the study, individuals in either the usual care or intervention group who become ineligible during the study (*e.g.*, self-report of a new condition in the list of exclusion criteria) will be informed that their participation has ended and will be provided the reason for ending their study participation.

No medications will be administered by the study staff. Procedures are not needed to transition subjects of study agents or alternate therapy if withdrawn from this study.

4.7.3 Data Collection and Follow-up for Withdrawn Subjects

Withdrawn patients after enrollment will be analyzed as intention to treat. Missing data will be imputed using multiple imputations along with sensitivity analyses for missingness according to the recommendations given in the National Research Council report.[129]

5 Study Procedures

5.1 Prior and Concomitant Therapy

All concomitant antihypertensive medications prescribed by the patient's healthcare team are permitted during the study. Ongoing antihypertensive medication changes by the patient's healthcare team (including medication initiation, discontinuation, and/or dose changes) are allowed throughout the study.

All other medications, supplements, and lifestyle modifications that were part of the participant's treatment plan prior to the study are included.

The participant may also be started on non-pharmacologic therapy (examples: physical activity, stress management) by their medical team during this study.

5.2 Randomization and Blinding of Intervention

Randomization assignments for both sites will be generated by the UW Biostatistics Clinical Trials Statistical Data Analysis Center stratified by research site, in block sizes of 4 and 6 to ensure equal allocation. The Research Electronic Data Capture (REDCap) clinical data management system will be used to verify eligibility and data completeness prior to randomization. The UW Biostatistics team will develop a randomization list, which will be provided to the MyHEART research staff in a binder. There will be two people responsible for the binder at all times at HIP (PI and research manager) who will give the randomization assignment upon receiving a phone call from the research assessor (from either site) conducting Visit 2. Until the staff receives a phone call, the binder will remain in

a locked file cabinet, separate from other research materials, within a locked office at 800 University Bay Drive (HIP).

All study participants will have contact with research staff (*e.g.*, research coordinators, research visit study assessment staff). To reduce bias, MyHEART 6- and 12-month assessors will be blinded to treatment assignments.[130] Assessors will be trained to treat patients in both study groups identically per protocol.[131] Ambulatory monitoring will be the gold standard for blood pressure measurements.

5.3 *Established Standard of Care*

Usual care and intervention arm participants will receive routine hypertension clinical care per their primary care provider. This includes the possibility of receiving untailored self-management resources (*i.e.,* dietician referral) at their provider's discretion, but this is not systematically tailored to young adults' needs. These patients would be treated according to current hypertension guideline standards including, but not limited to, hypertension lifestyle modification counseling: 1) reduced sodium intake, 2) weight loss, 3) reduced alcohol consumption, and 4) increased physical activity. This may include verbal discussions and/or handouts. Medications would be initiated and/or titrated per hypertension guidelines.

All patients would have clinic blood pressure checks and would be encouraged to have home blood pressure monitoring. Additionally, providers may order 24-hour Ambulatory Blood Pressure Monitoring, per the U.S. Preventive Services Task Force guidelines, to exclude white coat hypertension. Patients would also have additional tests not limited to blood draws, EKGs, echocardiograms, etc.

We will assess the number of healthcare team contacts in the usual care arm by calculating the number of primary care and specialty visits and dietician and exercise referrals after study enrollment (see IRB application with data variable sheet).[50,60] Usual care arm participants will receive AHA's "What is High Blood Pressure"[131] handout (copies of handouts are in the Appendix).[132] It is usual care for patients to receive hypertension educational material when presenting for a hypertension visit. They will not receive home blood pressure monitors. Usual care participants will have the same 6- and 12-month assessments as the intervention arm.

5.4 *Study Visits*

Study Calendar - see appendix

5.4.1 Screening/Baseline:

The same procedures will be followed at both research sites (Madison and Milwaukee).

5.4.1.1 Screening Visits:

Screening Visit 1: Screening Visit 1 occurs no more than 1 month after the telephone screening phone call. Written informed consent and HIPAA authorization will be obtained at the start of Screening Visit 1, which will include authorization for additional screening/eligibility assessment[133] (*e.g.*, 24-hour ambulatory blood pressure monitoring), gathering data, including information from the electronic health record, and all study procedures after enrollment, if eligible. If a potentially eligible patient requests to reschedule to sign the consent and/or HIPAA form, they may do so within 30 days of the screening phone call. Blood pressures will be measured. The patient is not eligible if the between-arm blood pressure difference is ≥20 mmHg. If these findings are noted, the participant will receive an incidental finding notification to discuss with their primary care provider. All patients will be given their clinic blood pressure in written format from Visit 1, regardless of eligibility. Remaining eligible patients will receive an ambulatory blood pressure (AMBP) monitor (example: SpaceLabs; final product to be determined in the appendix)[134] for 24-hour blood pressure

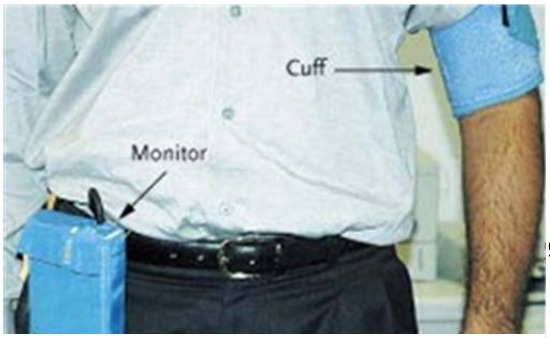

Figure 1. 24-Hour Ambulatory BP Monitoring

monitoring. The selected AMBP device will be FDA approved or have a 510(k) clearance. 24-hour AMBP monitoring is recommended to confirm a hypertension diagnosis (exclude white coat hypertension).[135-138] The U.S. Preventive Services Task Force noted up to 65% of patients with high office blood pressures were not diagnosed with hypertension after AMBP monitoring.[139] Previous young adult focus groups supported AMBP monitoring (see Figure 1) given its mobility and limited time commitment.

If a potentially eligible young adult already completed 24-hour ambulatory blood pressure monitoring through their UW Health or Aurora Health Care Clinic within the past 3 months, they do not need to repeat 24-hour ambulatory blood pressure monitoring for study eligibility. The ambulatory blood pressure monitoring report will be reviewed in their electronic health record to evaluate for white coat hypertension (an exclusion criteria). If eligible, the participant will be able to proceed with the Screening Visit 2/Baseline Assessment Visit. The individual's honorarium for Visit 1 and Visit 2 will not be adjusted/decreased.

Screening Visit 2/Baseline Assessment Visit: After receiving a 24-hour AMBP monitor, participants will be asked to return for Visit 2 on the next business day. Participants will have up to 30 days from Screening Visit 1 to return for Visit 2 without having to restart with the telephone screening process. A research staff member (not the individual acquiring consent or enrolling during this visit) will evaluate the mean 24-hour ambulatory blood pressure. If there is white coat hypertension, the patient is ineligible, and will receive an incidental finding letter to share with their primary care provider. Remaining eligible patients will be enrolled, randomized to MyHEART or usual care, and complete the baseline assessment.

5.4.1.2 Baseline/Follow-Up Data Collection:

All research staff will be trained in all key elements of the protocol and use of the data management system.

Anthropometric Measurements

- **Height** will be determined at each assessment visit using a wall-mounted stadiometer, without shoes or other outdoor footwear. Two measures of height will be taken and the average of the two will be used. We will repeat the measurement of height at each assessment visit because growth may still be occurring in young adults. Height will be measured to the nearest 0.1 cm.
- **Weight** will be determined at each assessment in light indoor clothes, removing all excess layers of clothing, and without shoes at all visits. All scales will be calibrated using standard weights, annually by the Bureau of Weights and Standards and quarterly by trained study personnel. Weight will be measured to the nearest 0.1 kg.
- **Body mass index (BMI).** The weight and height measures will be used to calculate Body Mass Index (weight in kilograms divided by height in meters squared; kg/m^2)
- **Waist Circumference** will be measured at baseline per established protocol/figures in the appendix.

Baseline Self-Reported Measures

All of the following measures will be recorded on paper by the participant, then electronically entered, with confirmation of correct entry, by the research staff via REDCap on an encrypted research computer within the research offices at UWHC and/or HIP. **See appendix for final forms and questions.**

- **Alcohol Use:** Alcohol consumption will be evaluated by alcohol beverage type and quantity as performed in the CARDIA (Coronary Artery Risk Development in Young Adults) cohort study; answered by self-report.[140]
- **Financial Status:** Participants will self-report their financial situation by selecting, for example, whether they: a) had enough money after paying bills for extra things (*e.g.*, dining out), b) enough to pay bills but not to purchase extra things, c) enough money to pay bills by cutting back on things, or d) difficulty paying bills no matter what was done. The first two answer choices are categorized as "adequate income" and the last two choices as "inadequate income".[32] This financial assessment accounts for varying income, which is more reflective of young adulthood.
- **Annual Household Income:**[141] income will be divided into six categories (for example: <$20,000, $20,000-34,999, $35,000-49,999, $50,000-$99,999, ≥$100,000, prefer not to answer) and recorded by

self-report

- **Self-Rated Health Status:** Single-item assessments of self-rated health status have a strong association with health outcomes[142-148] and provide a broad assessment of health.[149] Self-rated health will be measured using the following question/responses similar to: "In general, would you say your health is Excellent, Very Good, Good, Fair, or Poor?", which has been used in nationally representative samples.[147-149]
- **Health literacy** (*i.e.*, the ability to obtain, process, and understand basic information to make appropriate health decisions)[150-152] will be evaluated using a Rapid Estimate of Adult Literacy in Medicine instrument.[33,153,154]
- **Baseline comorbidities** (examples: dyslipidemia,[155] anxiety,[156] depression,[157] diabetes mellitus,[158] chronic kidney disease[159]) will be assessed via self-report and the electronic health record using established ICD codes as per previous research.[50,60,65,66,155-159]
- **Social Support** will be evaluated using a brief multi-dimensional evaluation validated for patients' chronic illnesses (example: The Medical Outcomes Study Social Support Survey).[149,160-162]
- **Global medication adherence** will be assessed using the Morisky adherence scale.[163,164]

Baseline Electronic Health Record and Other Database Data Collection

- **Healthcare utilization** will be evaluated using the John Hopkins Adjusted Clinical Group Case-Mix System, which assesses morbidity burden based on patient age, gender, and patterns of disease;[50,60,66,165] the number of clinic visits will be measured.
- **Baseline antihypertensive medications and other medications:** Number of antihypertensive medication classes will account for varying hypertension treatment at baseline. Data will be acquired from the electronic health record and participant self-report, and will include prescription and non-prescription medications.
- **Primary care provider characteristics** (age, gender, specialty, time since completed medical school, % of panel with hypertension, panel size) will be obtained when available from the electronic health record and/or the American Medical Association Masterfile data.[50,60]

Interviewer-Administered Data Collection (Baseline, 6 months, and 12 months)

- **Physical Activity.** We will assess physical activity using the Godin Exercise Questionnaire to evaluate weekly leisure activity and total leisure activity.
- **Automated Self-Administered 24-Hour (ASA24) Dietary Assessment Tool –** A web-based tool that enables multiple, automatically coded, self-administered 24-hour recalls. This tool will be used to assess, for example, sodium, fruit, and vegetable intake in relation to the DASH-sodium diet.

Blood Pressure Measurements

Blood pressures at each research site will be assessed with a Dinamap Monitor. Cuff size will be determined by arm circumference. An appropriate sized[166] blood pressure cuff will be connected to an automated sphygmomanometer (Dinamap). Each site will have the following cuff sizes available: small adult (for arm circumference of 22-26 cm), regular adult (27-34 cm), and large adult (35-42 cm). A blood pressure measurement will be obtained on the right upper arm, then the left upper arm, for the initial assessment. Right arm blood pressures will be subsequently used for the initial and follow-up visits, unless the left arm systolic blood pressure is ≥10 mmHg higher. The average of the second and third systolic and diastolic pressures, each taken 1 minute apart, will define the baseline clinic blood pressure.[167] If a participant's upper arm is too large, a forearm blood pressure will be obtained. All participants (intervention and control) will receive copies of their baseline, 6-month, and 12-month research clinic blood pressures to share with their usual healthcare provider, and will receive instructions on recommended primary care clinic follow-up.[43,168]

24-Hour Blood Pressure Monitoring:[169-171] 24-hour blood pressure monitoring will take place at baseline, 6 months, and 12 months. Staff trained in the use of the 24-hour ambulatory blood pressure monitor will apply the appropriate sized cuff to the subjects' non-dominant arms unless the systolic blood pressure difference as

measured during the clinic blood pressure assessment is >10 mmHg, in which case the arm with the highest value obtained will be used. ABPM placement procedures and device recording setting will be per the AMBP manufacturing company's instructions and hypertension guideline recommendations. Participants will be instructed to keep their arm still during cuff inflation, and on the use of a diary to record the time of their activities, sleep, symptoms, and medications. Participants will be given instructions on how and when to return the device to study staff. Data from the returned device will be uploaded and time-matched with information from the participants' diaries. Day-time and night-time will preferentially be defined using each participant's diary. All acquired readings will be saved and used for analysis. If the participant is unable to complete ambulatory blood pressure monitoring, they will not be excluded, but will alternatively have their blood pressure evaluated for study eligibility using the automatic office blood pressure (AOBP) technique per the SPRINT protocol[172] (example: have serial blood pressures acquired once every 5 minutes up to 5 times, using an automatic cuff without a research staff member present [*i.e.*, while sitting alone in the research clinic exam room]).

Home Blood Pressure Monitoring: Intervention participants will be provided an Omron home blood pressure monitor with an appropriate cuff size; details of the final model acquired for the study can be found in the appendix.[173,174] The selected monitor will be based upon validation studies in young adults and also across BMI categories.[175] Intervention arm participants will receive training and practice on proper cuff placement and monitor use during their baseline enrollment visit.[36] They will be asked to take their blood pressure at least three days a week, 2 measurements each time.[31,36] A normal home blood pressure (complicated and uncomplicated hypertension) is <135/85 mmHg,[176] which accounts for changes in hypertension guidelines for patients with diabetes and chronic kidney disease.[21,177] Participants will be able to read the recorded blood pressures to their health coach during the scheduled health coach phone calls (see below).[178] Participants will be asked to bring their home blood pressure monitor to the 6- and 12-month visits to review recorded blood pressures. Participants will keep the blood pressure monitor after study completion. Malfunctioned monitors will be replaced during the study.

5.4.2 Intervention

Health Coach Training: Health coaches will receive training that builds on their prior education in motivational interviewing[179] and hypertension management, using self-determination theory concepts. Training intensity is based on prior studies[127] and resources from our pilot: interactive didactic lectures, videos,[180] and the MyHEART health coach guide (see Appendix). Trainers will observe health coaches with role-playing, followed by problem solving and debriefing.[181-183] Health coaches will already be certified as an exercise physiologist (American College of Sports Medicine certification), registered dietician (Academy of Nutrition and Dietetics) with at least 3 years of clinical experience including chronic disease management and adult education/counseling, or a registered nurse. Health coaches will receive training on the MyHEART coaching guide that builds on their prior education in motivational interviewing and hypertension management, using self-determination theory concepts. Training will include interactive didactic lectures and videos and the MyHEART health coach guide. The trainer will also observe health coaches with role playing followed by problem solving and debriefing. The references (scientific articles, etc.) that will be given to the coach are optional reading materials for them to select based upon additional questions or training needs about motivational interviewing, self-determination theory, and/or blood pressure lowering. The individual needs of each coach will be identified during training and they will be directed to appropriate references. All subject handouts developed for this study will be reviewed during training with discussion on when and how to include the handouts to reinforce the telephone calls. Handouts will be distributed after each phone call either by postal mail or email (based upon subject's preference).

Health Coach Fidelity: An audio recorder will be placed on the table during the health coach calls. The goal is to record the coach's comments and adherence to the curriculum and protocol, and use of the self-determination theory. The participant's first name will be heard on the audio recording as he/she communicates with the participant. A recording device will NOT be attached to the phone or phone line. The secure storage and transmission of these audio files is summarized in the "*Protection Against Risks*" section.

Health Coach Phone Calls: Intervention arm participants will receive a health coach phone call every 2 weeks for 6 months, for a total of 12 calls. The first call will be scheduled at the screening visit and subsequent calls will be set

up at the end of each prior call. If a participant is unavailable, the health coach will leave a message and reschedule the call up to 3 times. Once a coach is ready to make calls for the MyHEART intervention: during each call, the health coaches will review and discuss home blood pressures, if available, from home blood pressure monitoring, and address barriers and concerns to home blood pressure monitoring. The MyHEART coaches will also recommend clinic follow-up based on the 2-week average home blood pressures – see coach's guide, which coincides with UW Health's blood pressure follow-up recommendation. During each telephone call, the coach will guide the subject on selecting health behavior goals and completing the goal sheet (uploaded in this application). In addition to home blood pressure monitoring, subjects will be able to choose which topics from the 8 additional hypertension education modules they want to address during a call (options include: hypertension knowledge, low sodium, DASH eating plan, weight loss/maintenance, smoking cessation, moderate alcohol consumption, blood pressure medicine, social support and stress management). The coach may also offer topics to the participant based upon barriers or concerns discussed during the call. A module is a "topic" to discuss which includes a coach's education/counseling, associated handouts, and references (see module overview in table below). The participant may focus on the same topics throughout the intervention based upon their personal needs or change the topics with every call. Home blood pressure monitoring will be discussed during each call by the health coach.

**MyHEART Health Coach Educational Modules**	
**Module**	**Topics**
Home Blood Pressure Monitoring	How to measure blood pressure, include members in household to avoid isolation, what do the numbers mean
Hypertension Knowledge	Define blood pressure, goal blood pressure, long-term health consequences
Low Sodium	Reading labels, effects of sodium on blood pressure, culturally appropriate cooking alternatives, eating with peers, meal planning, shopping on a budget
DASH Eating Plan	DASH components, meal planning, grocery shopping on a budget, DASH with peers; Cultural and personal food preferences; Discuss cultural meaning of dietary practices
Weight Loss/ Maintenance	Relationship of weight with hypertension, dietary and activity options to lose weight, time management, social support to assist with weight loss, fitness apps
Smoking Cessation*	Negative effects of tobacco on heart health, social/peer influence
Moderate Alcohol Consumption	Negative effects on heart health; define quantity for types of alcohol
Blood Pressure Medicine	Why medications may be needed, side effects, adherence, possible lifelong commitment, remembering medication, cost
Social Support	Local resources for support, reducing clinic no-shows, peer/social support
Stress Management	Identifying ways to reduce stress, stress with life's transitions and new challenges
*Only for active tobacco users	

The content of coach-participant interactions will focus on: 1) highlighting discrepancies between participants' current health behaviors and their desired behavior goals to promote internal motivation, 2) sharing reference points for guideline-recommended behaviors to lower blood pressure, and 3) discussing short-term goals and developing congruent action plans. Coaches will promote autonomy by individualizing the order and depth of educational content based on the behavioral goals chosen by the subject. Subjects will be coached on practical skill building (*e.g.*, label reading) and coaches will encourage patients to set goals that are motivating but not overwhelming. The "Coach's Guide" is to build on the coach's knowledge of blood pressure lowering behavioral techniques and how to use the self-determination theory and motivational interviewing to guide the subject as they initiate and try to maintain their new health behaviors. Abbreviated guides in the appendix highlight important "talking points" for the coach to address during each call. Hypertension guidelines on exercise and dietary changes will be given to the participant by the health coach, based upon the updated guidelines available at the time. Coach references will be updated if new guidelines and scientific recommendations become available during this study.

All intervention participants will start with the home blood pressure monitoring module. The first follow-up phone call will address hypertension knowledge and review home blood pressure monitoring. Home blood pressure monitoring

education will also be provided during all follow-up phone calls. The order of the remaining modules will be guided by participant's choice and tailored to their goals.[170,171] Fewer modules than calls allows some topics to be repeated as needed and per the participants' requests. Current tobacco users will be referred to the Wisconsin Tobacco Quit Line (http://www.ctri.wisc.edu/quitline.html) when ready to attempt cessation and their primary care provider will be notified of their intention to quit via the EHR. The participant will be encouraged to also discuss alcohol reduction/cessation with their primary care provider if they report consuming >14 alcohol beverages/week.[122] The MyHEART team created handouts to include specific topics requested by our young adult focus groups (see Appendix). The MyHEART handouts were formatted with a Flesch-Kincaid readability of ≤6th grade.[172]

5.4.3 Follow up:

Table. Acceptable Window for Study Visits (including weekends)

Visit	Window	Activities
Baseline (Visit 1)	No more than 30 days from telephone screening phone call	Written, informed consent, clinic blood pressure, 24-hour AMBP placement, provide participant remuneration
Baseline (Visit 2)	No sooner than 24 hours after Visit 1 and no more than 21 days after Visit 1	Clinic blood pressure, 24-hour AMBP removal, if eligible, enrollment, randomization, baseline screening questions and assessment, provide participant remuneration
6-month (Visit 3)	No later than 30 days since the last health coach call (intervention arm) or the scheduler call (routine/usual care)	Clinic blood pressure, 24-hour AMBP placement, 6-month follow-up assessment (ex: BMI), questionnaires, provide participant remuneration
6-month (Visit 4)	No sooner than 24 hours from Visit 3 and no more than 21 days from Visit 3	24-hour AMBP removal, provide participant remuneration
12-month (Visit 5)	No later than 30 days since scheduler call (both intervention and usual care arm)	Clinic blood pressure, 24-hour AMBP placement, 6-month follow-up assessment (ex: BMI), questionnaires, provide participant remuneration
12-month (Visit 6)	No sooner than 24 hours from Visit 5 and no more than 21 days from Visit 5	24-hour AMBP removal, provide participant remuneration

5.4.4 Unscheduled:

- Unscheduled visits are not included in this study.
- See the appendix for the study calendar with procedures and data collection
- Participants will be encouraged to continue routine blood pressure follow-up with their primary and routine healthcare team. Otherwise, no additional concomitant therapies be advised or suggested.

5.4.5 Final Study Visit

The final study evaluation will be two visits (as above in the visit table) with the first visit occurring 12 months after study enrollment. See appendix for the study calendar of study endpoints and evaluations.

During the 12-month visit, participants will be informed of their treatment assignments, final weight, clinic and 24-hour blood pressures, and body mass index. Additional data from the 12-month visit will not be analyzed in time to be provided to the participant.

Additional follow-up for adverse or serious adverse events will continue for 30-days after the final 12-month visit with one telephone follow-up by the research team. The participant will be responsible for scheduling any additional clinic visits and medical care for adverse or serious adverse events.

6 Study Analysis

6.1 Sample Size Determination

For Aim 1, approximately 930 individuals will be screened to get to a total of 310 enrolled participants (155 participants per randomized arm). This is designed under the assumption that there will be a further 15% dropout rate at the 6-month mark following randomization, to result in an effective sample size of 264.[184] In Nidich et al.,[185] the 3-month transcendental meditation program showed a mean difference of 6.3 and 4.0 in systolic and diastolic blood pressure change with a standard deviation of 13.6 and 9.7, respectively, as compared to a case of usual control among young adults at hypertension risk. With an effective sample size of 264, there is power of 0.94 and 0.87 for systolic and diastolic blood pressure, respectively, each at a two-tailed 0.025 test for an overall significance level of 0.05. For Aim 2, the effective sample size of 264 subjects will provide 90% power to detect a difference in the population mean difference from baseline to 6 months between treatment groups of about 0.4 standard deviations of the differences (moderate effect size). Aim 3 is an exploratory aim.

6.2 Statistical Methods

See Appendix for the full Statistical Analysis Plan (SAP)

Statistical Analysis Plan Overview:

Data will be analyzed by intent to treat. Missing data will be imputed using multiple imputations.

For Aim 1, the comparisons for the primary outcomes of systolic and diastolic blood pressure change from baseline to 6 months will be done using analysis of covariance. The primary comparisons for the secondary outcome of hypertension control at 6 months will be based on Fisher's exact tests. Sequential hypothesis testing will also be performed. The 12-month analysis will focus on *maintenance* of behavior change, to assess whether significant differences seen between baseline and 6 months are retained. We will also assess the differential effectiveness of the intervention across subgroups by developing exploratory regression models.

For Aim 2, behavioral outcomes will be analyzed in a similar manner as with the clinical outcomes in Aim 1 depending on whether they are continuous or categorical. Analyses (t-tests or Wilcoxon tests and ANCOVA for continuous variables; Fisher's exact test for the categorical variable) will be performed for differences from baseline to 6 months between treatment groups. Linear or generalized linear mixed-effects regression models will be fit to describe longitudinal behavioral measurements over time.

For Aim 3, analyses will examine hypothesized mediators of the MyHEART intervention (for example, autonomy support, internal motivation, perceived competence, and patient activation). We will estimate mediation effects in multilevel models[186-190] where mediation is assessed by fitting two models. Standard errors and 95% confidence intervals for the mediation effect and percent-mediated estimates will be calculated. This analysis will be pivotal to guide future replication and dissemination of MyHEART.

6.3 Planned Interim Analysis:

There is no interim analysis planned. However, we are prepared, if requested by the Data Safety and Monitoring Committee at any point, to calculate interim statistical power for its review. Projections of interim power can be made under several scenarios for future data, including assumptions that current trends continue or that the future

data reflect the relative effects used in the design of the trial. Safety reports will tally adverse events by intervention assignment and postulated relationship to the trial interventions; event rates will be reported per person year of follow-up. Should excessive risk to study participants be determined during the data safety monitoring review, the study will be stopped and all participants notified in a manner appropriate to the nature of the risk as defined by the IRB and DSMB.

7 Data Collection, Handling, and Record Keeping

7.1 Data Confidentiality

Protecting Participant Confidentiality: Every effort will be made to protect participant confidentiality. Subject confidentiality will be protected with the coded patient IDs whenever possible. Linking information will be maintained separately from other study documents in a password-protected database on password-protected media. Only the principal investigator, HIP programmers, and the research coordinators responsible for mailing the study invitations and remuneration will have access to directly identifiable subject data. Once these activities have ceased, identifying information will be destroyed. Analysis datasets will be limited datasets and the research team will not have access to identifiers other than those in a limited dataset (zip codes and dates), or any cross-walk information.

Identifiable Data: HIP and Aurora Health Care Programmers will identify potential participants using the criteria described in this protocol. Each subject will be assigned a study-specific ID number, maintained separately from the subject's medical record number (MRN). Only research team members listed in the IRB application shall have access to the list.

Security levels for the UW Ob/Gyn and Health Innovation Program's data systems that include patient-identifiable data are housed within a UW Health data center (and benefit from the same security configuration as other UW Health servers housing fully identifiable clinical and administrative patient data) and include:

1. All new user account requests and access to any resource is reviewed and approved by the Health Innovation Program Director or UW Ob/Gyn IT director.
2. Resource administration is managed by the UW Ob/Gyn or Health Innovation Program's (HIP's) Compliance Officer and Information Technology personnel. UW Health user account creation is requested by HIP's Compliance Officer and fulfilled by UW Health Information Technology personnel.
    a. A combination of Active Directory (AD) and New Technology File System (NTFS) folder permissions enforce authentication and file access policies.
    b. Folder access permissions are routinely reviewed and compared to the project's list of "Key Personnel" and/or those persons specified elsewhere to have privileges to view/use the data (*e.g.*, data use agreement or confidentiality agreement signatories).
3. These data systems are located behind UW Health's firewall.
4. These data systems are accessed via remote desktop connections originating from a subnet of Internet Protocol (IP) addresses reserved only for UW Ob/Gyn or Health Innovation Program computer workstations.
5. These HIP data systems at UW Health are secured in a data center behind a series of two locked doors; access is restricted to key UW Health Information Technology personnel under the supervision of the UW Health server administrator.

PC workstations used to access the server with identifiable data must be within HIP's subnet, which is preapproved by UW Health to access this server and have an additional layer of password protection.

The Aurora Health Care Research Analytics team and the Aurora Health Care Information Technology personnel oversee the security, user access, and data systems that include patient-identifiable data and transfer of encrypted, limited data sets to the UW Ob/Gyn or Health Innovation Program. The research analytics team has received extensive training and numerous certifications on obtaining data from Aurora's electronic health record. Each member of the research analytics team may serve as honest brokers and are skilled in de-identifying protected health information.

For Aurora Health Care:

1. Aurora Health Care Research Analytics, Aurora IRB/Compliance, and Aurora Information Technology teams review all new user account requests and access to any resource.
2. Aurora Health Care's database servers are secured via firewall, hardened to remove nonessential access credentials, and strong password compliance. Hosted systems are constantly monitored for latencies and intrusion.
3. There are differentiated user roles and privileges, password and user authentication security, electronic signatures, SSL encryption, and a comprehensive auditing record and monitoring of access and data changes to support the security of data transfer from Aurora Health Care to the Health Innovation Program.

At the Aurora site, paper source documents will be kept in a clinical research office at Aurora Health Care (Milwaukee, WI) which is double locked and only accessible by key card and/or key. After REDCap entry, source documents will be stored in subject binders in a locked filing cabinet and maintained for the length of time approved by the IRB.

Analysis Datasets: All research and analysis from the combined research sites will be conducted using a limited dataset (no direct identifiers; includes zip codes and dates only), provided by the Health Innovation Program.

Each individual patient record in the dataset is assigned a "pseudo ID" during dataset construction, which allows HIP and Aurora Health Care Programmers to link patient records from multiple sources and/or across different file types (*e.g.*, demographics, laboratory results, medications). The pseudo ID also allows Programmers to cross-walk the data back to the Electronic Health Record ("EHR"; example: UW Health Link) in the event data need to be verified or additional elements extracted from the source. Information allowing data to be cross-walked with identifiers will never be shared with the study team.

Following data aggregation, linking, and variable creation, the datasets used for day-to-day analysis will be securely transferred to a second server with equivalent security. Data access is restricted to only IRB-approved personnel conducting study analysis and with passwords for computer drive and project folder access. The limited datasets are stored on a specified HIP server, housed in the Centennial Office Building managed by the UW SMPH IT, and is connected via fiber optic cable to the HIP suite at 800 University Bay Drive. As of May 2022, all data will be transferred from HIP and stored on the UW Department of Obstetrics and Gynecology server. No individual PHI will be released for analysis, in presentation or publication. Only aggregate statistical output representing groups of subjects will be released. The completed study data set will be securely transferred, using the latest Secure File Transfer Protocol, to the Bioinformatics Computing Group (BCG) at the UW-Madison to the lead statistician. The levels of security for the server are five-fold and include:

1) Physical Security: server is located in an enterprise level secure data center under control of UW School of Medicine and Public Health (SMPH) ITS, which is a dedicated computer machine room requiring keycard and PIN for access. The room is equipped with camera surveillance, a hot and cold isle chilling system and an automatic fire detection and suppression system. SMPH ITS does not have access to the server. Ob-Gyn servers are supplied power by redundant sources and are protected by both an uninterruptible power supply and a backup generator.

2) The data resides behind an SMPH managed hardware based firewall and a computer based software firewall

3) Access controls: Data directory access is limited to project PI and designees she approves (currently none at this time)

4) Domain access restrictions: access to Ob-Gyn computing resources is restricted to individuals with an Ob-Gyn logon

5) Authentication: Password protection is used at the network level for all transactions that allow entry and editing of data, provide access to EPHI data, or administrative activities.

Online Collaboration Tool: The Health Innovation Program developed a secure SharePoint-based Intranet. This web-based platform will be available to IRB-approved MyHEART investigators and staff to securely access this study's materials (examples: up-to-date protocols, contact information, calendars). Access to the UW Ob/Gyn or HIP Intranet follows the same secure measures as defined above.

Online Reporting Tool: This proposal will use the REDCap data management program. The REDCap application and data are hosted by the Bioinformatics Computing Group (BCG) at the UW-Madison on its server farm with redundant systems. To gain access to the REDCap system, study personnel will need to fill out a REDCap account request through ICTR using the following link: https://redcap.ictr.wisc.edu/surveys/?s=MPHF4FWP4D.

Once approved, each user will have their own account. To ensure that REDCap users have access only to data and information that they are supposed to have access to within the application, the each user's privileges will be assigned by the study's principal investigator. The *Data Access Groups* functionality will also be implemented to allow site-specific research staff to only access records created at their respective site.

Additional REDCap security features that will be used in this study include:

- authentication to validate the identity of end-users that log into the system
- auto-logout setting
- Limit number of failed login attempts before a user is locked out of the system

REDCap data entry, views, and modification will be audited for each user. A user's status will be immediately suspended if an audit demonstrates that they are not complying with the protocol and the IRB and DSMB will be notified per the IRB policy.

Disaster recovery plans include a back-up server at the UW Centennial Medical Building, independently managed by the UW School of Medicine and Public Health, Department of Medicine.

We also have policies and procedures in place to protect the confidentiality and security of patient data, and our data protection measures (for protected health information, or PHI) are consistent with the Federal Health Insurance Portability and Protection Act's privacy and security rules (45 CFR §160.103; 45 CFR §160 and 164, Subparts A and C, respectively). Only persons directly involved in the research will have access to identifiable research data. All study data will be maintained in password-protected, secured computer files or in locked cabinets within locked offices.

7.1.1 Confidentiality of Subject Records

By signing the protocol, the Investigator agrees that the NIH/NHLBI or IRB representative may consult and/or copy study documents in order to verify CRF data. By signing the consent form, the subject agrees to this process. If study documents will be photocopied during the process of verifying CRF information, the subject will be identified by unique code only and full names and similar identifying information (such as medical record number or social security number) will be masked.

The Clinical Site Investigators will ensure that the identity of subjects will be protected. All study records will be maintained in a secure fashion with access limited to essential study personnel only. All study documents submitted to the Coordinating Center will have identifiers removed other than dates of birth and service and subjects will be identified with a study-specific identification number only. The Clinical Site Investigators will maintain, in a secure location, an enrollment log that includes subject identifying information and links subjects to their study-specific identification number.

7.2 Data Capture

7.2.1 Source Documents

All source data will be kept and merged and/or entered into the electronic clinical trial software (REDCap). Source data is all information, original records of clinical findings, observations, or other activities in a clinical trial necessary for the reconstruction and evaluation of the trial. Source data are contained in source documents. Examples of these original documents and data records include: hospital records, clinical and office charts, laboratory notes, memoranda, subjects' diaries or evaluation checklists, recorded data from automated instruments, copies or transcriptions certified after verification as being accurate and complete, subject files, and records kept at the pharmacy, at the laboratories, and at medico-technical departments involved in the clinical trial.

Data will be collected at both study sites during the Screening Visit (Visit 1), Screening/Baseline assessment (Visit 2), 6-month Visits (Visits 3 and 4), 12-month Visits (Visits 5 and 6), and during the health coach phone calls.

In addition, 24-ambulatory blood pressure monitoring will be performed at each site with data downloaded via Spacelabs software and summary data transferred to REDCap. Data from these visits including participant demographic information, vital signs, and other participant information will be uploaded into REDCap clinical research management software.

7.2.2 Case Report Forms

The study case report form (CRF) is a data reporting instrument for the study. All data requested on the CRF must be recorded. All missing data must be explained.

- All entries should be printed legibly in black ink.
- If any entry error has been made, to correct such an error, draw a single straight line through the incorrect entry and enter the correct data above it.
- All such changes must be initialed and dated.

NOTE:

- If a space on the CRF is left blank because the procedure was not done or the question was not asked, write “N/D”.
- If the item is not applicable to the individual case, write “N/A”.
- DO NOT ERASE OR WHITE OUT ERRORS.
- For clarification of illegible or uncertain entries, print the clarification above the item, then initial and date it.

7.3 Data Management

The Madison site, under the direction of MyHEART’s lead statistician, will serve as the data coordinating center and the primary site to oversee timely and accurate data collection.

7.4 Data Monitoring

REDCap reports will be used to provide up-to-the-minute access to all entered data. This will allow verification of completeness, timeliness, reliability, and accuracy of collection and coding of data. Quality reports will also be sent to the MyHEART Steering Committee and will include comprehensive data on all quality control activities, including protocol adherence and violation, training, retraining, certification, and site visit reporting. The Steering Committee, along with the UW Biostatistics and Medical Informatics’ lead MyHEART statistician, will develop and maintain standards to identify outlying values, and initiate and coordinate separate review of these observations for accuracy. Consistency checks and range checks have been built into the REDCap System.

7.5 Records Retention

For this non-FDA regulated study, all study data will be retained for at least 2 years after the last manuscript published under this clinical trials protocol number. If the analysis data will be retained beyond this window (example: for another study), a separate protocol and IRB approval will be obtained. The analysis data sets will only be shared with the Aurora Health Care research site as per the study structure outlined above. Any other outside institution will need to establish a data use agreement per UW Health’s, University of Wisconsin School of Medicine & Public Health, and any other applicable party’s protocol to acquire a copy of the de-identified dataset.

8 Assessment of Safety

Centralized safety oversight will be coordinated by the PI. The site PI will be notified of any adverse/serious adverse events for their specific site and will notify the PI, to ensure that all notifications (IRB, DSMB, etc.) occur according to protocol.

8.1 Specifications of Safety Parameters

8.1.1 Definition of Adverse Events (AE)

An *adverse event* (AE) is any symptom, sign, illness, or experience that develops or worsens in severity during the course of the study. Intercurrent illnesses or injuries should be regarded as adverse events. Abnormal results of diagnostic procedures are considered to be adverse events if the abnormality:

- results in study withdrawal
- is associated with a serious adverse event
- is associated with clinical signs or symptoms
- leads to additional treatment or to further diagnostic tests
- is considered by the investigator to be of clinical significance

8.1.2 Definition of Serious Adverse Events (SAE)

Adverse events are classified as serious or non-serious. A *serious adverse event* is any AE that fulfills at least one of the following criteria:

- is fatal
- is life-threatening
- requires or prolongs hospital stay
- results in persistent or significant disability or incapacity
- is a congenital anomaly or birth defect
- is an important medical event

Important medical events are those that may not be immediately life threatening, but are clearly of major clinical significance. They may jeopardize the subject, and may require intervention to prevent one of the other serious outcomes noted above. For example, treatment of bronchospasm in an emergency department would typically be considered serious. The primary pre-defined serious adverse events related to this study include hypoglycemia among patients with diabetes mellitus starting a new exercise program. All adverse events that do not meet any of the criteria for 'serious' should be regarded as *non-serious adverse events*. Throughout the study, any new clinically significant findings/abnormalities that meet the definition of an adverse event must also be recorded and documented as an adverse event.

Post-Study Adverse Event: All unresolved adverse events will be followed by the investigator until the events are resolved, the participant is lost to follow-up, or the adverse event is otherwise explained. At the last scheduled visit, the research visit investigator will instruct each participant to report any subsequent event(s) that the participant, or the participant's personal physician, believes might be related to participation in this study; the staff will contact the participant 30-days after study completion to inquire about any adverse events. The research visit investigator should notify the site-PI and the principal investigator (if different from the site-PI). The principal investigator will notify the study sponsor and IRB of any serious adverse event or death occurring up to 30 days after the participant has discontinued or terminated study participation that may be related to this study.

Hospitalization, Prolonged Hospitalization, or Surgery: Any adverse event that results in hospitalization or prolonged hospitalization will be documented and reported as a serious adverse event unless specifically instructed

otherwise in this protocol. Any condition responsible for surgery should be documented as an adverse event if the condition meets the criteria for an adverse event.

Neither the condition, hospitalization, prolonged hospitalization, nor surgery are reported as an adverse event in the following circumstance:

- Hospitalization or prolonged hospitalization for diagnostic or elective surgical procedures for a preexisting condition. Surgery should ***not*** be reported as an outcome of an adverse event if the purpose of the surgery was elective or diagnostic and the outcome was uneventful.

8.1.3 Definition of Unanticipated Problems (UP)

OHRP considers unanticipated problems involving risks to participants or others to include, in general, any incident, experience, or outcome that meets **all** of the following criteria:

- Unexpected in terms of nature, severity, or frequency given (a) the research procedures that are described in the protocol-related documents, such as the IRB-approved research protocol and informed consent document; and (b) the characteristics of the participant population being studied;
- Related or possibly related to participation in the research ("possibly related" means there is a reasonable possibility that the incident, experience, or outcome may have been caused by the procedures involved in the research); and
- Suggests that the research places participants or others at a greater risk of harm (including physical, psychological, economic, or social harm) than was previously known or recognized.

This study will use the OHRP definition of UP.

Corrective actions or changes that will be considered in response to an UP will include:

- Modification of inclusion or exclusion criteria to mitigate the newly identified risks
- Implementation of additional safety monitoring procedures
- Suspension of enrollment of new participants or halting of study procedures for enrolled participants
- Modification of informed consent documents to include a description of newly recognized risks
- Provision of additional information about newly recognized risks to previously enrolled participants.

8.2 Classification of an Adverse Event

8.2.1 Severity of Event

The following guidelines will be used to describe severity:

Mild – Events require minimal or no treatment and do not interfere with the participant's daily activities.

Moderate – Events result in a low level of inconvenience or concern with the therapeutic measures. Moderate events may cause some interference with functioning.

Severe – Events interrupt a participant's usual daily activity and may require systemic drug therapy or other treatment. Severe events are usually potentially life threatening or incapacitating.

8.2.2 Expectedness

The principal investigator will be responsible for determining whether an AE is expected or unexpected. An AE will be considered unexpected if the nature, severity, or frequency of the event is not consistent with the risk information previously described for the study agent.

8.3 Time Period and Frequency for Event Assessment and Follow-Up

The occurrence of an AE or SAE may come to the attention of study personnel during study visits and interviews of a study participant presenting for medical care, or upon review by a study monitor. All AEs including local and systemic reactions not meeting the criteria for SAEs will be captured on the appropriate CRF. Information to be collected includes event description, time of onset, clinician's assessment of severity, relationship to study product (assessed only by those with the training and authority to make a diagnosis), and time of resolution/stabilization of the event. All AEs occurring while on study must be documented appropriately regardless of relationship. All AEs will be followed to adequate resolution.

Any medical condition that is present at the time that the participant is screened will be considered as baseline and not reported as an AE. However, if the study participant's condition deteriorates at any time during the study, it will be recorded as an AE. UPs will be recorded in the data collection system throughout the study.

Changes in the severity of an AE will be documented to allow an assessment of the duration of the event at each level of severity to be performed. AEs characterized as intermittent require documentation of onset and duration of each episode.

The PI will record all reportable events with start dates occurring any time after informed consent is obtained until 30 days (for non-serious AEs and SAEs) after the last day of study participation. At each study visit, the investigator will inquire about the occurrence of AE/SAEs since the last visit. Events will be followed for outcome information until resolution or stabilization. Breaches of confidentiality and emotional upset will be reported to the PI who will then review the incident with the study staff, information technology groups, and follow-up with the participant. The PI is responsible for making the reports to the DSBM, IRB and NHLBI.

8.4 Reporting procedures

This study will follow the HS-IRB reporting and submission timeframes:
https://kb.wisc.edu/images/group78/18324/ReportingTimeframesJanuary2013.pdf

Reporting time frames are in the Appendix.

This document will take priority for timeframes. The PI is responsible for reporting to the IRB, DSMB, and NIH.

8.4.1 Adverse Event (AE) Reporting

Adverse events must be reported once the participant undergoes any study procedures and adverse events must be reported during the entire active study period and for 30 days following the last administration of study treatment. The IRB, DSMB and NHLBI will be notifications about AEs.

8.4.2 Serious Adverse Event (SAE) Reporting

The study clinician will complete a SAE Form within the following timelines:

All deaths and immediately life-threatening events, whether related or unrelated, will be recorded on the SAE Form and submitted to the DCC/study sponsor within 24 hours of site awareness. See **Section 1, Key Roles** for contact information. Other SAEs, regardless of relationship, will be submitted to the DCC/study sponsor within 72 hours of site awareness.

All SAEs will be followed until satisfactory resolution or until the site investigator deems the event to be chronic or the adherence to be stable. Other supporting documentation of the event may be requested by the DCC/study sponsor and should be provided as soon as possible.

8.4.3 Unanticipated Problem (UP) Reporting

The site investigator will be responsible for creating and completing a UP report form. Incidents that meet the OHRP criteria for UPs will be reported promptly per the HS IRB timeline (see Appendix).

- All UPs should be reported to appropriate institutional officials as required by the HS-IRB, the NHLBI, DSMB, and OHRP upon receipt of the report of the problem from the investigator per the HS IRB policy: https://kb.wisc.edu/images/group78/18324/ReportingTimeframesJanuary2013.pdf

The UP report will include the following information:

- Protocol identifying information: protocol title and number, PI's name, and the IRB project number;
- A detailed description of the event, incident, experience, or outcome;
- An explanation of the basis for determining that the event, incident, experience, or outcome represents an UP;
- A description of any changes to the protocol or other corrective actions that have been taken or are proposed in response to the UP.

8.4.4 Reporting of Pregnancy

Subjects will also be immediately withdrawn from the study if they report being pregnant or planning to become pregnancy. Study data acquired up to the withdrawal date will be analyzed. No additional routine pregnancy reporting is indicated for this study.

8.5 Study Halting Rules

This study may be temporarily suspended or prematurely terminated if there is sufficient reasonable cause. Written notification, documenting the reason for study suspension or termination, will be provided by the suspending or terminating party to all site investigators, the NIH/NHLBI, DSMB, and IRB. If the study is prematurely terminated or suspended, the PI will promptly inform the IRB and will provide the reason(s) for the termination or suspension.

Circumstances that may warrant termination or suspension include, but are not limited to:

- Determination of unexpected, significant, or unacceptable risk to participants
- Demonstration of efficacy that would warrant stopping
- Insufficient compliance to protocol requirements
- Data that are not sufficiently complete and/or evaluable
- Determination of futility

Study may resume once concerns about safety, protocol compliance, data quality are addressed and satisfy the sponsor, IRB, and/or DSMB. All participant data that was collected prior to study termination will be analyzed and continue to be handled/stored per this protocol.

Subsequent review of serious, unexpected, and related AEs by the DSMB and/or IRB may also result in suspension of the study.

8.6 Safety Oversight

Independent safety oversight will be provided by The UW ICTR Data Monitoring Committee (DMC). It is an independent team supported in its mission of safety and compliance by experienced ICTR staff to provide administrative assistance, experienced members representing a diversity of backgrounds (clinicians, biostatisticians, bioethicist), skills, and knowledge, and the use of the Research Electronic Data Capture (REDCap) tool, which provides data management functionality by allowing the development of eCRFs and surveys to support data capture. In providing oversight for the conduct of this study, the ICTR DMC will meet annually (every 12

months) during the 5-year study with opportunities to meet face-to-face or via phone and web depending on each individual's availability. The number of individuals will be determined by the UW ICTR. Additional meetings may be scheduled as determined by the DMC or as requested by the PI. The DMC members will review protocol-specific reports created by statisticians that serve a non-voting member role on the DMC using data pulled from REDCap. These standard reports will include an overview of study objectives, a review of actual and projected accrual rates, an evaluation of patient demographics for balance of randomization, and a summary of the number and seriousness of adverse events. An interim analysis of study results may be performed and source documents may be reviewed to allow the DMC to independently judge whether the overall integrity and conduct of the protocol remain acceptable based on data provided and reported by the Principal Investigator. The DMC will make recommendations to the Principal Investigator that could include actions of continuation, modification, suspension, or termination.

Data will be summarized and provided directly to the DSMB through the REDCap clinical trial software used for data collection during the study. The DSMB will keep minutes of its meetings, and the PIs and all study staff will receive verbal and written summaries of their reviews and recommendations.

See the DSMB charter in the Appendix

8.7 Unblinding Procedure

Although unlikely in this study, unblinding will be done in emergent medical circumstances. All efforts will be made to maintain blinding except in the case of urgent medical necessity. If a subject needs to be unblinded, the study site must document who broke the blind and the reason, and report the event to a member of the trial's executive leadership team (principal investigator, site investigator, and senior biostatistician) within 24 hours, who will instruct the data coordinating center to unblind.

9 Study Monitoring, Auditing, and Inspecting

9.1 Medical Monitoring

9.1.1 Study Monitoring Plan

Clinical site monitoring is conducted to ensure that the rights and well-being of human subjects are protected, that the reported trial data are accurate, complete, and verifiable, and that the conduct of the trial is in compliance with the currently approved protocol/amendment(s) and with applicable regulatory requirement(s).

- The PI and site PI will conduct monitoring, including on-site and centralized (via REDCap), initially weekly for the early/initial enrollment procedures, and then monthly. The monitoring will be comprehensive (100% data verification) via REDCap with random review of health coach calls. Monitoring reports will be available for the DSMB.
- Each clinical site will also perform internal quality management of study conduct, data collection, documentation, and completion as directed by ICTR/WINHR. An individualized quality management plan will be developed to describe a site's quality management.

  See appendix for the auditing tool.

9.2 Protocol Deviations

A protocol deviation is any noncompliance with the clinical trial protocol or MOP requirements. The noncompliance may be either on the part of the participant, the investigator, or the study site staff. As a result of deviations, corrective actions are to be developed by the site and implemented promptly.

These will be reported per the HS IRB requirements (see appendix – reporting timeline). All deviations must be addressed in study source documents, reported to the NIH Program Official, DSMB, and IRB. Protocol deviations will be sent to the local IRB per their guidelines. The PI, site PI, and study staff are responsible for knowing and adhering to the IRB requirements.

9.2.1 Internal Data and Safety Monitoring Board

The Data Safety and Monitoring Plan (DSMP) for this research comprises research conducted at all sites for this proposal. All investigators and site principal investigators must agree to comply with the procedures outlined in this DSMP. This DSMP does not reduce any investigator's obligation to comply with the requirements of the Institutional Review Board (IRB).

All research activities conform to the NIH definition of a clinical trial. The UW ICTR Data Monitoring Committee (DMC) is an independent team supported in its mission of safety and compliance by experienced ICTR staff to provide administrative assistance, experienced members representing a diversity of backgrounds (clinicians, biostatisticians, bioethicist), skills, and knowledge, and the use of the Research Electronic Data Capture (REDCap) tool, which provides data management functionality by allowing the development of eCRFs and surveys to support data capture. In providing oversight for the conduct of this study, the ICTR DMC will meet annually (every 12 months) during the 5-year study with opportunities to meet face-to-face or via phone and web depending on each individual's availability. The number of individuals will be determined by the UW ICTR. Additional meetings may be scheduled as determined by the DMC or as requested by the PI. The DMC members will review protocol-specific reports created by statisticians that serve a non-voting member role on the DMC using data pulled from REDCap. These standard reports will include an overview of study objectives, a review of actual and projected accrual rates, an evaluation of patient demographics for balance of randomization, and a summary of the number and seriousness of adverse events. An interim analysis of study results may be performed and source documents may be reviewed to allow the DMC to independently judge whether the overall integrity and conduct of the protocol remain acceptable based on data provided and reported by the Principal Investigator. The DMC will make recommendations to the Principal Investigator that could include actions of continuation, modification, suspension, or termination.

Data will be summarized and provided directly to the DSMB through the REDCap clinical trial software used for data collection during the study. The DSMB will keep minutes of its meetings, and the PIs and all study staff will receive verbal and written summaries of their reviews and recommendations.

See the DSMB charter in the Appendix section of the protocol.

9.3 Auditing and Inspecting

The investigator will permit study-related monitoring, audits, and inspections by the IRB (or their representatives), and/or the NIH of all study related documents (*e.g.*, source documents, regulatory documents, data collection instruments, study data etc.). The investigator will ensure the capability for inspections of applicable study-related facilities (*e.g.*, pharmacy, diagnostic laboratory, etc.).

9.4 Subject Compliance Monitoring

The study team will assess and track subject compliance through previously published methods.[191] This will include:

- Intervention arm: Assessing the frequency that patients answer and complete health coach calls
- Intervention arm: Reviewing home blood pressure monitor directly during follow-up research visits and assessing the presence/absence of home reading availability during health coach calls
- Usual care and intervention arms: Frequency upon returning for study visits, completing phone surveys

After at least 3 call attempts, each 1-week apart, and 1 mailed letter attempt, if the participant does not return for the 6-month research study visit they will be withdrawn from the study and analyzed as intention to treat.

10 Ethical Considerations

This study is to be conducted according to NIH and Institutional research policies and procedures.

This protocol and any amendments will be submitted to a properly constituted independent Institutional Review Board (IRB), in agreement with local legal prescriptions, for formal approval of the study conduct. The decision of the IRB concerning the conduct of the study will be made in writing to the investigator and a copy of this decision will be provided to the sponsor before commencement of this study. The investigator should provide a list of IRB members and their affiliate to the sponsor.

All subjects for this study will be provided a consent form describing this study and providing sufficient information for subjects to make an informed decision about their participation in this study. See Appendix for a copy of the Subject Informed Consent Form. This consent form will be submitted with the protocol for review and approval by the IRB for the study. The formal consent of a subject, using the IRB-approved consent form, must be obtained before that subject undergoes any study procedure. The consent form must be signed by the subject and the investigator-designated research professional obtaining the consent.

11 Study Finances

11.1 Funding Source

This study is financed through a grant from the US National Institutes of Health.

11.2 Conflict of Interest

Any investigator who has a conflict of interest with this study (patent ownership, royalties, or financial gain greater than the minimum allowable by their institution, etc.) must have the conflict reviewed by a properly constituted Conflict of Interest Committee with a Committee-sanctioned conflict management plan that has been reviewed and approved by the study sponsor prior to participation in this study. All UW investigators will follow the UW conflict of interest policy.

12 Publication Plan

Neither the complete nor any part of the results of the study carried out under this protocol will be published or passed on to any third party without the consent of the principal investigator. The principal investigator holds the primary responsibility for publication of the results of the study. Study data and results will be published and shared as mandated by NIH and Federal data sharing regulations. We will share data generated from this study in the form of manuscripts submitted for peer-review and publication in appropriate journals. The timely release and sharing of data will coincide with dates on which manuscripts are accepted and subsequent page proofs are shared on PubMed/Medline as “Epub ahead of print” documents.

13 References

1. Go AS, Bauman MA, Coleman King SM, et al. An effective approach to high blood pressure control: a science advisory from the American Heart Association, the American College of Cardiology, and the Centers for Disease Control and Prevention. *J Am Coll Cardiol.* 2014;63(12):1230-1238.

2. U.S. Department of Health and Human Services. Healthy People 2020: Heart Disease and Stroke - Increase the proportion of adults with hypertension whose blood pressure is under control. http://www.healthypeople.gov/node/4555/data_details. Accessed March 23, 2017.

3. Centers for Disease Control and Prevention. High Blood Pressure Facts. http://www.cdc.gov/bloodpressure/facts.htm. Accessed March 23, 2017.

4. Egan BM, Zhao Y, Axon RN. US trends in prevalence, awareness, treatment, and control of hypertension, 1988-2008. *JAMA.* 2010;303(20):2043-2050.

5. Mozaffarian D, Benjamin EJ, Go AS, et al. Heart Disease and Stroke Statistics-2016 Update: A Report From the American Heart Association. *Circulation.* 2016;133(4):e38-e360.

6. Nguyen QC, Tabor JW, Entzel PP, et al. Discordance in national estimates of hypertension among young adults. *Epidemiology.* 2011;22(4):532-541.

7. Baker DW, Williams MV, Parker RM, Gazmararian JA, Nurss J. Development of a brief test to measure functional health literacy. *Patient Educ Couns.* 1999;38(1):33-42.

8. Grubbs V, Lin F, Vittinghoff E, et al. Body mass index and early kidney function decline in young adults: a longitudinal analysis of the CARDIA (Coronary Artery Risk Development in Young Adults) study. *Am J Kidney Dis.* 2014;63(4):590-597.

9. Mitchell AB, Cole JW, McArdle PF, et al. Obesity increases risk of ischemic stroke in young adults. *Stroke.* 2015;46(6):1690-1692.

10. Schold JD, Goldfarb DA, Buccini LD, et al. Comorbidity burden and perioperative complications for living kidney donors in the United States. *Clin J Am Soc Nephrol.* 2013;8(10):1773-1782.

11. Centers for Disease Control and Prevention. Vital signs: awareness and treatment of uncontrolled hypertension among adults--United States, 2003-2010. *MMWR Morb Mortal Wkly Rep.* 2012;61:703-709.

12. Smith TW, Ruiz JM. Psychosocial influences on the development and course of coronary heart disease: current status and implications for research and practice. *J Consult Clin Psychol.* 2002;70(3):548-568.

13. Appel LJ, Moore TJ, Obarzanek E, et al. A clinical trial of the effects of dietary patterns on blood pressure. DASH Collaborative Research Group. *N Engl J Med.* 1997;336(16):1117-1124.

14. Jones DW, Peterson ED. Improving hypertension control rates: technology, people, or systems? *JAMA.* 2008;299(24):2896-2898.

15. Kearney PM, Whelton M, Reynolds K, et al. Global burden of hypertension: analysis of worldwide data. *Lancet.* 2005;365(9455):217-223.

16. Bautista LE. High Blood Pressure. In: Remington PL, Brownson RC, Wegner MV, eds. *Chronic Disease, Epidemiology, and Control*. 3rd ed. Washington, DC: American Public Health Association; 2010:335-362.

17. Brosius FC, 3rd, Hostetter TH, Kelepouris E, et al. Detection of chronic kidney disease in patients with or at increased risk of cardiovascular disease: a science advisory from the American Heart Association Kidney and Cardiovascular Disease Council; the Councils on High Blood Pressure Research, Cardiovascular Disease in the Young, and Epidemiology and Prevention; and the Quality of Care and Outcomes Research Interdisciplinary Working Group: Developed in Collaboration With the National Kidney Foundation. *Hypertension.* 2006;48(4):751-755.

18. Kernan WN, Dearborn JL. Obesity increases stroke risk in young adults: opportunity for prevention. *Stroke.* 2015;46(6):1435-1436.

19. Lane C, Huws-Thomas M, Hood K, et al. Measuring adaptations of motivational interviewing: the development and validation of the behavior change counseling index (BECCI). *Patient Educ Couns.* 2005;56(2):166-173.

20. Lloyd-Jones DM, Leip EP, Larson MG, et al. Prediction of lifetime risk for cardiovascular disease by risk factor burden at 50 years of age. *Circulation.* 2006;113(6):791-798.

21. Chobanian AV, Bakris GL, Black HR, et al. The Seventh Report of the Joint National Committee on Prevention, Detection, Evaluation, and Treatment of High Blood Pressure: the JNC 7 report. *JAMA.* 2003;289(19):2560-2572.

22. Ford ES, Ajani UA, Croft JB, et al. Explaining the decrease in U.S. deaths from coronary disease, 1980-2000. *N Engl J Med.* 2007;356(23):2388-2398.

23. Lloyd-Jones DM, Hong Y, Labarthe D, et al. Defining and setting national goals for cardiovascular health promotion and disease reduction: the American Heart Association's strategic Impact Goal through 2020 and beyond. *Circulation.* 2010;121(4):586-613.

24. Paramore LC, Halpern MT, Lapuerta P, et al. Impact of poorly controlled hypertension on healthcare resource utilization and cost. *Am J Manag Care.* 2001;7(4):389-398.

25. Yano Y, Stamler J, Garside DB, et al. Isolated Systolic Hypertension in Young and Middle-Aged Adults and 31-Year Risk for Cardiovascular Mortality: The Chicago Heart Association Detection Project in Industry Study. *J Am Coll Cardiol.* 2015;65(4):327-335.

26. Holland N, Segraves D, Nnadi VO, et al. Identifying barriers to hypertension care: implications for quality improvement initiatives. *Dis Manag.* 2008;11(2):71-77.

27. James PA, Oparil S, Carter BL, et al. 2014 evidence-based guideline for the management of high blood pressure in adults: report from the panel members appointed to the Eighth Joint National Committee (JNC 8). *JAMA.* 2014;311(5):507-520.

28. Ma J, Urizar GG, Jr., Alehegn T, Stafford RS. Diet and physical activity counseling during ambulatory care visits in the United States. *Prev Med.* 2004;39(4):815-822.

29. Weber MA, Schiffrin EL, White WB, et al. Clinical practice guidelines for the management of hypertension in the community: a statement by the American Society of Hypertension and the International Society of Hypertension. *J Clin Hypertens (Greenwich).* 2014;16(1):14-26.

30. Artinian NT, Flack JM, Nordstrom CK, et al. Effects of nurse-managed telemonitoring on blood pressure at 12-month follow-up among urban African Americans. *Nurs Res.* 2007;56(5):312-322.

31. Bosworth HB, Olsen MK, Grubber JM, et al. Two self-management interventions to improve hypertension control: a randomized trial. *Ann Intern Med.* 2009;151(10):687-695.

32. Bosworth HB, Olsen MK, Grubber JM, Powers BJ, Oddone EZ. Racial differences in two self-management hypertension interventions. *Am J Med.* 2011;124(5):468 e461-468.

33. Bosworth HB, Olsen MK, Neary A, et al. Take Control of Your Blood Pressure (TCYB) study: a multifactorial tailored behavioral and educational intervention for achieving blood pressure control. *Patient Educ Couns.* 2008;70(3):338-347.

34. Bosworth HB, Powers BJ, Olsen MK, et al. Home blood pressure management and improved blood pressure control: results from a randomized controlled trial. *Arch Intern Med.* 2011;171(13):1173-1180.

35. Friedberg JP, Rodriguez MA, Watsula ME, et al. Effectiveness of a tailored behavioral intervention to improve hypertension control: primary outcomes of a randomized controlled trial. *Hypertension.* 2015;65(2):440-446.

36. Green BB, Cook AJ, Ralston JD, et al. Effectiveness of home blood pressure monitoring, Web communication, and pharmacist care on hypertension control: a randomized controlled trial. *JAMA.* 2008;299(24):2857-2867.

37. Jackson GL, Oddone EZ, Olsen MK, et al. Racial differences in the effect of a telephone-delivered hypertension disease management program. *J Gen Intern Med.* 2012;27(12):1682-1689.

38. Kim KB, Han HR, Huh B, et al. The effect of a community-based self-help multimodal behavioral intervention in Korean American seniors with high blood pressure. *Am J Hypertens.* 2014;27(9):1199-1208.

39. Margolius D, Bodenheimer T, Bennett H, et al. Health coaching to improve hypertension treatment in a low-income, minority population. *Ann Fam Med.* 2012;10(3):199-205.

40. Mehos BM, Saseen JJ, MacLaughlin EJ. Effect of pharmacist intervention and initiation of home blood pressure monitoring in patients with uncontrolled hypertension. *Pharmacotherapy.* 2000;20(11):1384-1389.

41. Ralston JD, Cook AJ, Anderson ML, et al. Home blood pressure monitoring, secure electronic messaging and medication intensification for improving hypertension control: a mediation analysis. *Appl Clin Inform.* 2014;5(1):232-248.

42. Rudd P, Miller NH, Kaufman J, et al. Nurse management for hypertension. A systems approach. *Am J Hypertens.* 2004;17(10):921-927.

43. Appel LJ, Champagne CM, Harsha DW, et al. Effects of comprehensive lifestyle modification on blood pressure control: main results of the PREMIER clinical trial. *JAMA.* 2003;289(16):2083-2093.

44. Boulware LE, Daumit GL, Frick KD, et al. An evidence-based review of patient-centered behavioral interventions for hypertension. *Am J Prev Med.* 2001;21(3):221-232.

45. Glynn LG, Murphy AW, Smith SM, Schroeder K, Fahey T. Interventions used to improve control of blood pressure in patients with hypertension. *Cochrane Database Syst Rev.* 2010(3):CD005182.

46. Ogedegbe G, Tobin JN, Fernandez S, et al. Counseling African Americans to Control Hypertension (CAATCH) trial: a multi-level intervention to improve blood pressure control in hypertensive blacks. *Circ Cardiovasc Qual Outcomes.* 2009;2(3):249-256.

47. Senesael E, Borgermans L, Van De Vijver E, Devroey D. Effectiveness of a quality improvement intervention targeting cardiovascular risk factors: are patients responsive to information and encouragement by mail or post? *Vasc Health Risk Manag.* 2013;9:13-20.

48. Watson AJ, Singh K, Myint UK, et al. Evaluating a web-based self-management program for employees with hypertension and prehypertension: a randomized clinical trial. *Am Heart J.* 2012;164(4):625-631.

49. Magid DJ, Ho PM, Olson KL, et al. A multimodal blood pressure control intervention in 3 healthcare systems. *Am J Manag Care.* 2011;17(4):e96-103.

50. Johnson HM, Thorpe CT, Bartels CM, et al. Antihypertensive medication initiation among young adults with regular primary care use. *J Gen Intern Med.* 2014;29(5):723-731.

51. Pinto E. Blood pressure and ageing. *Postgrad Med J.* 2007;83(976):109-114.

52. Allen NB, Siddique J, Wilkins JT, et al. Blood pressure trajectories in early adulthood and subclinical atherosclerosis in middle age. *JAMA.* 2014;311(5):490-497.

53. Michie S, Abraham C, Whittington C, McAteer J, Gupta S. Effective techniques in healthy eating and physical activity interventions: a meta-regression. *Health Psychol.* 2009;28(6):690-701.

54. Sher T, Braun L, Domas A, et al. The partners for life program: a couples approach to cardiac risk reduction. *Fam Process.* 2014;53(1):131-149.

55. Voils CI, Gierisch JM, Yancy WS, Jr., et al. Differentiating behavior initiation and maintenance: theoretical framework and proof of concept. *Health Educ Behav.* 2014;41(3):325-336.

56. Agency for Healthcare Research and Quality (AHRQ). *Patient Self-Management Support Programs: An Evaluation*. Rockville, MD: Agency for Healthcare Research and Quality (AHRQ):48. 08-0011.

57. Magid DJ, Olson KL, Billups SJ, et al. A pharmacist-led, American Heart Association Heart360 Web-enabled home blood pressure monitoring program. *Circ Cardiovasc Qual Outcomes.* 2013;6(2):157-163.

58. Margolis KL, Asche SE, Bergdall AR, et al. Effect of home blood pressure telemonitoring and pharmacist management on blood pressure control: a cluster randomized clinical trial. *JAMA.* 2013;310(1):46-56.

59. Magid DJ, Farmer SA. Hypertension self-management: a home run for patients and payers. *Circ Cardiovasc Qual Outcomes.* 2014;7(2):205-206.

60. Johnson HM, Thorpe CT, Bartels CM, et al. Undiagnosed hypertension among young adults with regular primary care use. *J Hypertens.* 2014;32(1):65-74.

61. Johnson HM, Warner RC, LaMantia JN, Bowers BJ. "I have to live like I'm old." Young adults' perspectives on managing hypertension: a multi-center qualitative study. *BMC Fam Pract.* 2016;17(1):31.

62. O'Brien K, Venn BJ, Perry T, et al. Reasons for wanting to lose weight: different strokes for different folks. *Eat Behav.* 2007;8(1):132-135.

63. Underbakke G, McBride PE. Office systems for heart disease prevention. *Prim Care.* 2005;32(4):883-900.

64. Wolpert HA, Anderson BJ. Young adults with diabetes: need for a new treatment paradigm. *Diabetes Care.* 2001;24(9):1513-1514.

65. Johnson HM, Bartels CM, Thorpe CT, et al. Differential Diagnosis and Treatment Rates Between Systolic and Diastolic Hypertension in Young Adults: A Multidisciplinary Observational Study. *J Clin Hypertens (Greenwich).* 2015;17(11):885-894.

66. Johnson HM, Olson AG, LaMantia JN, et al. Documented lifestyle education among young adults with incident hypertension. *J Gen Intern Med.* 2014;30(5):556-564.

67. Mozaffarian D, Benjamin EJ, Go AS, et al. Heart disease and stroke statistics—2015 update: a report from the American Heart Association. *Circulation.* 2015;131(4):e29-e322.

68. Johnson HM, Warner RC, Bartels CM, LaMantia JN. "They're younger... it's harder." Primary providers' perspectives on hypertension management in young adults: a multicenter qualitative study. *BMC Res Notes.* 2017;10(1):9.

69. Bodenheimer T, Lorig K, Holman H, Grumbach K. Patient self-management of chronic disease in primary care. *JAMA.* 2002;288(19):2469-2475.

70. Jolly K, Duda JL, Daley A, et al. Evaluation of a standard provision versus an autonomy promotive exercise referral programme: rationale and study design. *BMC Public Health.* 2009;9:176.

71. Kinnafick FE, Thogersen-Ntoumani C, Duda JL. Physical activity adoption to adherence, lapse, and dropout: a self-determination theory perspective. *Qual Health Res.* 2014;24(5):706-718.

72. Markland D, Ryan RM, Tobin VJ, Rollnick S. Motivational interviewing and self-determination theory. *J Soc Clin Psychol.* 2005;24(6):811-831.

73. Scales R, Miller JH. Motivational techniques for improving compliance with an exercise program: skills for primary care clinicians. *Curr Sports Med Rep.* 2003;2(3):166-172.

74. Carroll JK, Fiscella K, Epstein RM, Sanders MR, Williams GC. A 5A's communication intervention to promote physical activity in underserved populations. *BMC Health Serv Res.* 2012;12:374.

75. Zoellner J, Thomson JL, Landry AS, et al. Improvements in blood pressure among undiagnosed hypertensive participants in a community-based lifestyle intervention, Mississippi, 2010. *Prev Chronic Dis.* 2014;11:E53.

76. Kupst MJ, Butt Z, Stoney CM, et al. Assessment of stress and self-efficacy for the NIH Toolbox for Neurological and Behavioral Function. *Anxiety Stress Coping.* 2015:1-14.

77. Molton IR, Jensen MP, Nielson W, Cardenas D, Ehde DM. A preliminary evaluation of the motivational model of pain self-management in persons with spinal cord injury-related pain. *J Pain.* 2008;9(7):606-612.

78. Deci EL, Eghrari H, Patrick BC, Leone DR. Facilitating internalization: the self-determination theory perspective. *J Pers.* 1994;62(1):119-142.

79. Deci EL, Ryan RM. *Intrinsic Motivation and Self-Determination in Human Behavior*. New York, NY: Plenum Press; 1985.

80. Deci EL, Ryan RM. Human Autonomy: The Basis for True Self-Esteem. In: Kernis MH, ed. *Efficacy, Agendy, and Self-Esteem*. New York, NY: Plenum Press; 1995:31-49.

81. Deci EL, Ryan RM. The "what" and "why" of goal pursuits: human needs and the self-determination of behavior. *Psychol Inq.* 2000;11(4):227-268.

82. Deci EL, Ryan RM. Self-determination theory: a macrotherapy of human motivation, development, and health. *Can Psychol.* 2008;49(3):182-185.

83. Li K, Iannotti RJ, Haynie DL, Perlus JG, Simons-Morton BG. Motivation and planning as mediators of the relation between social support and physical activity among U.S. adolescents: a nationally representative study. *Int J Behav Nutr Phys Act.* 2014;11(1):42.

84. Lorig KR, Holman H. Self-management education: history, definition, outcomes, and mechanisms. *Ann Behav Med.* 2003;26(1):1-7.

85. Lorig KR, Sobel DS, Ritter PL, Laurent D, Hobbs M. Effect of a self-management program on patients with chronic disease. *Eff Clin Pract.* 2001;4(6):256-262.

86. Roth G. Perceived parental conditional regard and autonomy support as predictors of young adults' self- versus other-oriented prosocial tendencies. *J Pers.* 2008;76(3):513-534.

87. Ryan RM, Frederick CM, Lepes D, Rubio N, Sheldon KM. Intrinsic motivation and exercise adherence. *Int J Sport Psychol.* 1997;28:335-354.

88. Silva MN, Markland D, Minderico CS, et al. A randomized controlled trial to evaluate self-determination theory for exercise adherence and weight control: rationale and intervention description. *BMC Public Health.* 2008;8:234.

89. Williams GC, Deci EL, Ryan RM. Building Health-Care Partnerships by Supporting Autonomy: Promoting Maintained Behavior Chage and Positive Health Outcomes. In: Suchman AL, Botelho RJ, Hinton-Walker P, eds. *Partnerships in Healthcare: Transforming Relational Process*. Rochester, NY: University of Rochester Press; 1998:67-87.

90. Bosworth HB, Oddone EZ, Weinberger M. *Patient Treatment Adherence: Concepts, Interventions, and Measurement*. Mahwah, NJ: Lawrence Erlbaum Associates, Inc.; 2006.

91. Institute of Medicine (US) Committee on Public Health Priorities to Reduce and Control Hypertension. *A Population-Based Policy and Systems Change Approach to Prevent and Control Hypertension*. Washington, D.C.: National Academies Press (US); 2010.

92. Ho PM, Rumsfeld JS. Beyond inpatient and outpatient care: alternative model for hypertension management. *BMC Public Health.* 2006;6:257.

93. Walsh JM, McDonald KM, Shojania KG, et al. Quality improvement strategies for hypertension management: a systematic review. *Med Care.* 2006;44(7):646-657.

94. The Diabetes Prevention Program (DPP): description of lifestyle intervention. *Diabetes Care.* 2002;25(12):2165-2171.

95. Bokhour BG, Cohn ES, Cortes DE, et al. The role of patients' explanatory models and daily-lived experience in hypertension self-management. *J Gen Intern Med.* 2012;27(12):1626-1634.

96. Willard-Grace R, DeVore D, Chen EH, et al. The effectiveness of medical assistant health coaching for low-income patients with uncontrolled diabetes, hypertension, and hyperlipidemia: protocol for a randomized controlled trial and baseline characteristics of the study population. *BMC Fam Pract.* 2013;14:27.

97. Bosworth HB, Olsen MK, Gentry P, et al. Nurse administered telephone intervention for blood pressure control: a patient-tailored multifactorial intervention. *Patient Educ Couns.* 2005;57(1):5-14.

98. Lovejoy TI, Heckman TG, Suhr JA, et al. Telephone-administered motivational interviewing reduces risky sexual behavior in HIV-positive late middle-age and older adults: a pilot randomized controlled trial. *AIDS Behav.* 2011;15(8):1623-1634.

99. Madlensky L, Natarajan L, Flatt SW, et al. Timing of dietary change in response to a telephone counseling intervention: evidence from the WHEL study. *Health Psychol.* 2008;27(5):539-547.

100. Miller SM, Hui SK, Wen KY, et al. Tailored telephone counseling to improve adherence to follow-up regimens after an abnormal pap smear among minority, underserved women. *Patient Educ Couns.* 2013;93(3):488-495.

101. Funk KL, Elmer PJ, Stevens VJ, et al. PREMIER--a trial of lifestyle interventions for blood pressure control: intervention design and rationale. *Health Promot Pract.* 2008;9(3):271-280.

102. Frohlich ED. Detection, evaluation, and treatment of hypertension: JNC-5 (Joint National Committee on Detection, Evaluation, and Treatment of High Blood Pressure). *Heart Dis Stroke.* 1993;2(6):459-460.

103. The Sixth Report of the Joint National Committee on Prevention, Detection, Evaluation, and Treatment of High Blood Pressure. In: National Heart L, and Blood Institute, ed. Bethesda, MD: National Institutes of Health; 1997:70.

104. Intersalt: an international study of electrolyte excretion and blood pressure. Results for 24 hour urinary sodium and potassium excretion. Intersalt Cooperative Research Group. *BMJ.* 1988;297(6644):319-328.

105. Cutler JA, Follmann D, Allender PS. Randomized trials of sodium reduction: an overview. *Am J Clin Nutr.* 1997;65(2 Suppl):643S-651S.

106. Elliott P, Stamler J, Nichols R, et al. Intersalt revisited: further analyses of 24 hour sodium excretion and blood pressure within and across populations. Intersalt Cooperative Research Group. *BMJ.* 1996;312(7041):1249-1253.

107. Appel LJ, Brands MW, Daniels SR, et al. Dietary approaches to prevent and treat hypertension: a scientific statement from the American Heart Association. *Hypertension.* 2006;47(2):296-308.

108. Appel LJ. Lifestyle modification as a means to prevent and treat high blood pressure. *J Am Soc Nephrol.* 2003;14(7 Suppl 2):S99-S102.

109. Svetkey LP, Harsha DW, Vollmer WM, et al. Premier: a clinical trial of comprehensive lifestyle modification for blood pressure control: rationale, design and baseline characteristics. *Ann Epidemiol.* 2003;13(6):462-471.

110. Liese AD, Nichols M, Sun X, D'Agostino RB, Jr., Haffner SM. Adherence to the DASH Diet is inversely associated with incidence of type 2 diabetes: the insulin resistance atherosclerosis study. *Diabetes Care.* 2009;32(8):1434-1436.

111. Stamler J. Epidemiologic findings on body mass and blood pressure in adults. *Ann Epidemiol.* 1991;1(4):347-362.

112. Flegal KM, Carroll MD, Kuczmarski RJ, Johnson CL. Overweight and obesity in the United States: prevalence and trends, 1960-1994. *Int J Obes Relat Metab Disord.* 1998;22(1):39-47.

113. Ainsworth BE, Keenan NL, Strogatz DS, Garrett JM, James SA. Physical activity and hypertension in black adults: the Pitt County Study. *Am J Public Health.* 1991;81(11):1477-1479.

114. Reaven PD, Barrett-Connor E, Edelstein S. Relation between leisure-time physical activity and blood pressure in older women. *Circulation.* 1991;83(2):559-565.

115. Kelley G, McClellan P. Antihypertensive effects of aerobic exercise. A brief meta-analytic review of randomized controlled trials. *Am J Hypertens.* 1994;7(2):115-119.

116. Kelley GA, Kelley KA, Tran ZV. Aerobic exercise and resting blood pressure: a meta-analytic review of randomized, controlled trials. *Prev Cardiol.* 2001;4(2):73-80.

117. Eckel RH, Jakicic JM, Ard JD, et al. 2013 AHA/ACC guideline on lifestyle management to reduce cardiovascular risk: a report of the American College of Cardiology/American Heart Association Task Force on Practice Guidelines. *J Am Coll Cardiol.* 2014;63(25 Pt B):2960-2984.

118. Halanych JH, Safford MM, Kertesz SG, et al. Alcohol consumption in young adults and incident hypertension: 20-year follow-up from the Coronary Artery Risk Development in Young Adults Study. *Am J Epidemiol.* 2010;171(5):532-539.

119. MacMahon S. Alcohol consumption and hypertension. *Hypertension.* 1987;9(2):111-121.

120. Cushman WC. Alcohol consumption and hypertension. *J Clin Hypertens (Greenwich).* 2001;3(3):166-170.

121. Puddey IB, Beilin LJ, Vandongen R, Rouse IL, Rogers P. Evidence for a direct effect of alcohol consumption on blood pressure in normotensive men. A randomized controlled trial. *Hypertension.* 1985;7(5):707-713.

122. Puddey IB, Beilin LJ, Vandongen R. Regular alcohol use raises blood pressure in treated hypertensive subjects. A randomised controlled trial. *Lancet.* 1987;1(8534):647-651.

123. Viera AJ, Lingley K, Hinderliter AL. Tolerability of the Oscar 2 ambulatory blood pressure monitor among research participants: a cross-sectional repeated measures study. *BMC Med Res Methodol.* 2011;11:59.

124. Baral-Grant S, Haque MS, Nouwen A, Greenfield SM, McManus RJ. Self-Monitoring of Blood Pressure in Hypertension: A UK Primary Care Survey. *Int J Hypertens.* 2012;2012:582068.

125. U.S. Department of Health and Human Services. *The Fourth Report on the Diagnosis, Evaluation, and Treatment of High Blood Pressure in Children and Adolescents*. Bethesda, MD: National Institutes of Health - National Heart, Lung, and Blood Institute; 2005:1-60.

126. Sheehy AM, Flood GE, Tuan WJ, et al. Analysis of guidelines for screening diabetes mellitus in an ambulatory population. *Mayo Clin Proc.* 2010;85(1):27-35.

127. Blonstein AC, Yank V, Stafford RS, et al. Translating an evidence-based lifestyle intervention program into primary care: lessons learned. *Health Promot Pract.* 2013;14(4):491-497.

128. Rocha-Goldberg Mdel P, Corsino L, Batch B, et al. Hypertension Improvement Project (HIP) Latino: results of a pilot study of lifestyle intervention for lowering blood pressure in Latino adults. *Ethn Health.* 2010;15(3):269-282.

129. Rubin DB. *Multiple Imputation for Nonresponse in Surveys*. New York, NY: John Wiley & Sons; 1987.

130. Friedberg JP, Lipsitz SR, Natarajan S. Challenges and recommendations for blinding in behavioral interventions illustrated using a case study of a behavioral intervention to lower blood pressure. *Patient Educ Couns.* 2010;78(1):5-11.

131. Margolis KL, Kerby TJ, Asche SE, et al. Design and rationale for Home Blood Pressure Telemonitoring and Case Management to Control Hypertension (HyperLink): a cluster randomized trial. *Contemp Clin Trials.* 2012;33(4):794-803.

132. Department of Health and Human Services. Interventions to Improve Hypertension Control Rates in African Americans. http://grants.nih.gov/grants/guide/rfa-files/RFA-HL-04-007.html. Accessed March 23, 2017.

133. Fielding RA, Rejeski WJ, Blair S, et al. The Lifestyle Interventions and Independence for Elders Study: design and methods. *J Gerontol A Biol Sci Med Sci.* 2011;66(11):1226-1237.

134. Spacelabs Healthcare. OnTrak, the NEW Evolution of ABPM from Spacelabs Healthcare. http://www.spacelabshealthcare.com/diagnostic-cardiology/abp-monitoring/abp-monitors/ontrak#.VUuCLE10zct. Accessed March 23, 2017.

135. Green BB, Kaplan RC, Psaty BM. How do minor changes in the definition of blood pressure control affect the reported success of hypertension treatment? *Am J Manag Care.* 2003;9(3):219-224.

136. Little P, Barnett J, Barnsley L, et al. Comparison of agreement between different measures of blood pressure in primary care and daytime ambulatory blood pressure. *BMJ.* 2002;325(7358):254.

137. Pickering TG. 24 Hour Ambulatory Blood Pressure Monitoring: Is it Necessary to Establish a Diagnosis Before Instituting Treatment of Hypertension? *J Clin Hypertens (Greenwich).* 1999;1(1):33-40.

138. Piper MA, Evans CV, Burda BU, et al. Diagnostic and predictive accuracy of blood pressure screening methods with consideration of rescreening intervals: a systematic review for the U.S. preventive services task force. *Ann Intern Med.* 2015;162(3):192-204.

139. U.S. Preventive Services Task Force. Final Recommendation Statement: High Blood Pressure in Adults: Screening. http://www.uspreventiveservicestaskforce.org/Page/Document/RecommendationStatementFinal/high-blood-pressure-in-adults-screening. Accessed March 23, 2017.

140. Gaffo AL, Roseman JM, Jacobs DR, Jr., et al. Serum urate and its relationship with alcoholic beverage intake in men and women: findings from the Coronary Artery Risk Development in Young Adults (CARDIA) cohort. *Ann Rheum Dis.* 2010;69(11):1965-1970.

141. Fitzpatrick AL, Rapp SR, Luchsinger J, et al. Sociodemographic Correlates of Cognition in the Multi-Ethnic Study of Atherosclerosis (MESA). *Am J Geriatr Psychiatry.* 2015;23(7):684-697.

142. Idler EL, Benyamini Y. Self-rated health and mortality: a review of twenty-seven community studies. *J Health Soc Behav.* 1997;38(1):21-37.

143. Idler EL, Angel RJ. Self-rated health and mortality in the NHANES-I Epidemiologic Follow-up Study. *Am J Public Health.* 1990;80(4):446-452.

144. Idler EL, Kasl S. Health perceptions and survival: do global evaluations of health status really predict mortality? *J Gerontol.* 1991;46(2):S55-65.

145. Ho SC. Health and social predictors of mortality in an elderly Chinese cohort. *Am J Epidemiol.* 1991;133(9):907-921.

146. Idler EL, Russell LB, Davis D. Survival, functional limitations, and self-rated health in the NHANES I Epidemiologic Follow-up Study, 1992. First National Health and Nutrition Examination Survey. *Am J Epidemiol.* 2000;152(9):874-883.

147. DeSalvo KB, Jones TM, Peabody J, et al. Health care expenditure prediction with a single item, self-rated health measure. *Med Care.* 2009;47(4):440-447.

148. DeSalvo KB, Fan VS, McDonell MB, Fihn SD. Predicting mortality and healthcare utilization with a single question. *Health Serv Res.* 2005;40(4):1234-1246.

149. McDowell I. *Measuring Health: A Guide to Rating Scales and Questionnaires*. 3rd ed. New York, NY: Oxford University Press; 2006.

150. Nielsen-Bohlman L, Panzer AM, Kindig DA. *Health Literacy: A Prescription to End Confusion*. Washington, DC: The National Academies Press; 2004.

151. Kripalani S, Henderson LE, Chiu EY, et al. Predictors of medication self-management skill in a low-literacy population. *J Gen Intern Med.* 2006;21(8):852-856.

152. DeWalt DA, Pignone MP. Reading is fundamental: the relationship between literacy and health. *Arch Intern Med.* 2005;165(17):1943-1944.

153. Bass PF, 3rd, Wilson JF, Griffith CH. A shortened instrument for literacy screening. *J Gen Intern Med.* 2003;18(12):1036-1038.

154. Bass SB, Wolak C, Rovito GM, Gordon TF, Ward L. Use of REALM-R vs. S-TOFHLA in an urban African American clinic population to assess health literacy: Practical implications. *American Public Health Association 138th Annual Meeting & Expo: Social Justice*. Denver, CO; 2010.

155. Borzecki AM, Wong AT, Hickey EC, Ash AS, Berlowitz DR. Identifying hypertension-related comorbidities from administrative data: what's the optimal approach? *Am J Med Qual.* 2004;19(5):201-206.

156. Marciniak MD, Lage MJ, Dunayevich E, et al. The cost of treating anxiety: the medical and demographic correlates that impact total medical costs. *Depress Anxiety.* 2005;21(4):178-184.

157. Elixhauser A, Steiner C, Harris DR, Coffey RM. Comorbidity measures for use with administrative data. *Med Care.* 1998;36(1):8-27.

158. Hebert PL, Geiss LS, Tierney EF, et al. Identifying persons with diabetes using Medicare claims data. *Am J Med Qual.* 1999;14(6):270-277.

159. Foley RN, Murray AM, Li S, et al. Chronic kidney disease and the risk for cardiovascular disease, renal replacement, and death in the United States Medicare population, 1998 to 1999. *J Am Soc Nephrol.* 2005;16(2):489-495.

160. Shelbourne CD. Social Functioning: Social Activity Limitations Measure. In: Stewart AL, Ware JE, Jr., eds. *Measuring Functioning and Well-Being: The Medical Outcomes Study Approach*. Durham, NC: Duke University Press; 1992:173-181.

161. Shelbourne CD, Stewart AL, Wells KB. Role Functioning Measures. In: Stewart AL, Ware JE, Jr., eds. *Measuring Functioning and Well-Being: The Medical Outcomes Study Approach*. Durham, NC: Duke University Press; 1992:205-219.

162. Sherbourne CD, Stewart AL. The MOS social support survey. *Soc Sci Med.* 1991;32(6):705-714.

163. Morisky DE, Green LW, Levine DM. Concurrent and predictive validity of a self-reported measure of medication adherence. *Med Care.* 1986;24(1):67-74.

164. Bosworth HB, Powers B, Grubber JM, et al. Racial differences in blood pressure control: potential explanatory factors. *J Gen Intern Med.* 2008;23(5):692-698.

165. Starfield B, Weiner J, Mumford L, Steinwachs D. Ambulatory care groups: a categorization of diagnoses for research and management. *Health Serv Res.* 1991;26(1):53-74.

166. Pickering TG, Hall JE, Appel LJ, et al. Recommendations for blood pressure measurement in humans and experimental animals: part 1: blood pressure measurement in humans: a statement for professionals from the Subcommittee of Professional and Public Education of the American Heart Association Council on High Blood Pressure Research. *Circulation.* 2005;111(5):697-716.

167. Unger E, Diez-Roux AV, Lloyd-Jones DM, et al. Association of neighborhood characteristics with cardiovascular health in the multi-ethnic study of atherosclerosis. *Circ Cardiovasc Qual Outcomes.* 2014;7(4):524-531.

168. Victor RG, Ravenell JE, Freeman A, et al. Effectiveness of a barber-based intervention for improving hypertension control in black men: the BARBER-1 study: a cluster randomized trial. *Arch Intern Med.* 2011;171(4):342-350.

169. Grossman E. Ambulatory blood pressure monitoring in the diagnosis and management of hypertension. *Diabetes Care.* 2013;36 Suppl 2:S307-311.

170. Fravel MA, Ernst ME, Weber CA, et al. Influence of patient characteristics on success of ambulatory blood pressure monitoring. *Pharmacotherapy.* 2008;28(11):1341-1347.

171. Vollmer WM, Appel LJ, Svetkey LP, et al. Comparing office-based and ambulatory blood pressure monitoring in clinical trials. *J Hum Hypertens.* 2005;19(1):77-82.

172. Bakris GL. The Implications of Blood Pressure Measurement Methods on Treatment Targets for Blood Pressure. *Circulation.* 2016;134(13):904-905.

173. Coleman A, Freeman P, Steel S, Shennan A. Validation of the Omron 705IT (HEM-759-E) oscillometric blood pressure monitoring device according to the British Hypertension Society protocol. *Blood Press Monit.* 2006;11(1):27-32.

174. O'Brien E, Waeber B, Parati G, Staessen J, Myers MG. Blood pressure measuring devices: recommendations of the European Society of Hypertension. *BMJ.* 2001;322(7285):531-536.

175. Takahashi H, Yoshika M, Yokoi T. Validation of two automatic devices for the self-measurement of blood pressure according to the ANSI/AAMI/ISO81060-2:2009 guidelines: the Omron BP765 (HEM-7311-ZSA) and the Omron BP760N (HEM-7320-Z). *Vasc Health Risk Manag.* 2015;11:49-53.

176. Bosworth HB, Olsen MK, McCant F, et al. Hypertension Intervention Nurse Telemedicine Study (HINTS): testing a multifactorial tailored behavioral/educational and a medication management intervention for blood pressure control. *Am Heart J.* 2007;153(6):918-924.

177. American Diabetes Association. Standards of medical care in diabetes--2015. *Diabetes Care.* 2015;38 (Suppl 1):S1-S93.

178. Anthony CA, Polgreen LA, Chounramany J, et al. Outpatient blood pressure monitoring using bi-directional text messaging. *J Am Soc Hypertens.* 2015;9(5):375-381.

179. Miller WR, Rose GS. Toward a theory of motivational interviewing. *Am Psychol.* 2009;64(6):527-537.

180. Madson MB, Loignon AC, Lane C. Training in motivational interviewing: a systematic review. *J Subst Abuse Treat.* 2009;36(1):101-109.

181. Bellg AJ, Borrelli B, Resnick B, et al. Enhancing treatment fidelity in health behavior change studies: best practices and recommendations from the NIH Behavior Change Consortium. *Health Psychol.* 2004;23(5):443-451.

182. Carroll JK, Fiscella K, Epstein RM, et al. Physical activity counseling intervention at a federally qualified health center: improves autonomy-supportiveness, but not patients' perceived competence. *Patient Educ Couns.* 2013;92(3):432-436.

183. Spillane V, Byrne MC, Byrne M, et al. Monitoring treatment fidelity in a randomized controlled trial of a complex intervention. *J Adv Nurs.* 2007;60(3):343-352.

184. Wing RR, Tate DF, Espeland MA, et al. Innovative Self-Regulation Strategies to Reduce Weight Gain in Young Adults: The Study of Novel Approaches to Weight Gain Prevention (SNAP) Randomized Clinical Trial. *JAMA Intern Med.* 2016;176(6):755-762.

185. Nidich SI, Rainforth MV, Haaga DA, et al. A randomized controlled trial on effects of the Transcendental Meditation program on blood pressure, psychological distress, and coping in young adults. *Am J Hypertens.* 2009;22(12):1326-1331.

186. Bauer DJ, Preacher KJ, Gil KM. Conceptualizing and testing random indirect effects and moderated mediation in multilevel models: new procedures and recommendations. *Psychol Methods.* 2006;11(2):142-163.

187. Kenny DA, Korchmaros JD, Bolger N. Lower level mediation in multilevel models. *Psychol Methods.* 2003;8(2):115-128.

188. Krull JL, MacKinnon DP. Multilevel mediation modeling in group-based intervention studies. *Eval Rev.* 1999;23(4):418-444.

189. Krull JL, MacKinnon DP. Multilevel Modeling of Individual and Group Level Mediated Effects. *Multivariate Behav Res.* 2001;36(2):249-277.

190. Preacher KJ, Zyphur MJ, Zhang Z. A general multilevel SEM framework for assessing multilevel mediation. *Psychol Methods.* 2010;15(3):209-233.

191. Spiker B. Methods of assessing and improving patient compliance in clinical trials. *IRB: Ethics & Human Research.* 1992;14(3):1-6.

Attachments/Appendix (see separate table of contents for complete contents)

- Study Procedures Flowchart/Table
- Delegation log
- Study Calendar
- DMC/DSMB charter and composition
- Health coach curriculum, call logs, instructions
- AMBP instructions
