# Supplementary material for: Effect of a Telephone Health Coaching Intervention on Hypertension Control in Young Adults: The MyHEART Randomized Clinical Trial
Source: JAMA Netw Open. 2023 Feb 3;6(2):e2255618. doi: 10.1001/jamanetworkopen.2022.55618 (PMC9898821; doi:10.1001/jamanetworkopen.2022.55618)
Supplement: Supplement 2. — eTable 1. Baseline Participant Characteristics eTable 2. ASA-24 Dietary Component Evaluation of Sodium, Combined Fruit and Vegetables, Whole Grain, and Saturated Fat Intake, Comparing Baseline With 6 and 12 Months eTable 3. Sensitivity Analyses [file jamanetwopen-e2255618-s002.pdf]

# Supplemental Online Content

Hoppe KK, Smith M, Birstler J, et al. Effect of a telephone health coaching intervention on hypertension control in young adults: the MyHEART randomized clinical trial. *JAMA Netw Open.* 2023;6(2):e2255618. doi:10.1001/jamanetworkopen.2022.55618

**eTable 1.** Baseline Participant Characteristics
**eTable 2.** ASA-24 Dietary Component Evaluation of Sodium, Combined Fruit and Vegetables, Whole Grain, and Saturated Fat Intake, Comparing Baseline With 6 and 12 Months
**eTable 3.** Sensitivity Analyses

This supplemental material has been provided by the authors to give readers additional information about their work.

© 2023 Hoppe KK et al. *JAMA Network Open.*

eTable 1. Baseline Participant Characteristics

| Variable | Overall | 6-month follow-up | No 6-month follow-up | p-value |
|---|---|---|---|---|
| Total | 316 | 204 | 112 | |
| Age | 33.8 [18.0-39.0] | 33.8 [18.0-39.0] | 33.6 [19.0-39.0] | 0.541 |
| Alcohol beverages/week | 5.2 [0.0-32.0] | 5.6 [0.0-32.0] | 4.6 [0.0-30.0] | 0.192 |
| Ambulatory diastolic BP (mmHg) | 86.7 [68.0-120.0] | 86.6 [68.0-110.0] | 86.9 [71.0-120.0] | 0.786 |
| Ambulatory systolic BP (mmHg) | 132.8 [108.0-183.0] | 132.6 [108.0-172.0] | 133.2 [110.0-183.0] | 0.858 |
| Body mass index (kg/m2) | 34.5 [19.3-63.1] | 34.0 [19.3-61.9] | 35.3 [19.5-63.1] | 0.274 |
| Number of children at home | 1.0 [0.0-6.0] | 1.1 [0.0-6.0] | 0.9 [0.0-6.0] | 0.56 |
| Office diastolic BP (mmHg) | 91.3 [66.0-136.3] | 90.6 [66.0-130.3] | 92.6 [74.0-136.3] | 0.554 |
| Office systolic BP (mmHg) | 138.1 [106.0-192.0] | 137.8 [106.0-192.0] | 138.8 [109.7-187.7] | 0.929 |
| Waist circumference (cm) | 109.4 [65.0-178.0] | 109.0 [65.0-162.0] | 110.3 [68.0-178.0] | 0.554 |
| Weight (kg) | 103.2 [45.0-206.0] | 101.9 [53.0-200.0] | 105.8 [45.0-206.0] | 0.446 |
| Cigarette tobacco status | | | | 0.003 |
| I currently smoke cigarettes | 40 (12.7) | 17 (8.3) | 23 (20.5) | |
| I have never smoked cigarettes | 212 (67.1) | 149 (73.0) | 63 (56.2) | |
| I used to smoke cigarettes | 59 (18.7) | 38 (18.6) | 21 (18.8) | |
| Godin-Shephard Physical Activity | | | | 0.95 |
| Active | 149 (47.2) | 98 (48.0) | 51 (45.5) | |
| Insufficiently active | 162 (51.3) | 106 (52.0) | 56 (50.0) | |
| Highest level of education | | | | 0.62 |
| I have not finished high school | 15 (4.7) | 11 (5.4) | 4 (3.6) | |
| I have completed high school | 42 (13.3) | 19 (9.3) | 23 (20.5) | |
| I have not finished college or vocation school | 52 (16.5) | 32 (15.7) | 20 (17.9) | |
| I finished college or vocational school | 137 (43.4) | 97 (47.5) | 40 (35.7) | |
| Some/completed Graduate or Professional School | 65 (20.6) | 45 (22.1) | 20 (17.9) | |
| Marital status | | | | 0.345 |
| Single | 104 (32.9) | 63 (30.9) | 41 (36.6) | |
| Married/partnered | 200 (63.3) | 137 (67.2) | 63 (56.2) | |

© 2023 Hoppe KK et al. *JAMA Network Open.*

| | | | | |
|---|---|---|---|---|
| Divorced/widower | 7 (2.2) | 4 (2.0) | 3 (2.7) | |
| Number of antihypertensive medications | | | | 0.421 |
| 0 | 162 (51.3) | 100 (49.0) | 62 (55.4) | |
| 1 | 97 (30.7) | 66 (32.4) | 31 (27.7) | |
| 2 | 43 (13.6) | 28 (13.7) | 15 (13.4) | |
| 3 | 11 (3.5) | 9 (4.4) | 2 (1.8) | |
| greater than or equal to 4 | 3 (0.9) | 1 (0.5) | 2 (1.8) | |
| Race | | | | < 0.001 |
| Black | 72 (22.8) | 42 (20.6) | 30 (26.8) | |
| Other | 22 (7.0) | 7 (3.4) | 15 (13.4) | |
| White non-Hispanic | 222 (70.3) | 155 (76.0) | 67 (59.8) | |
| Self-perceived health status | | | | 0.352 |
| Excellent | 7 (2.2) | 5 (2.5) | 2 (1.8) | |
| Very good or good | 153 (48.4) | 106 (52.0) | 47 (42.0) | |
| Fair | 111 (35.1) | 64 (31.4) | 47 (42.0) | |
| Poor or no response | 39 (12.3) | 28 (13.7) | 11 (9.8) | |
| Sex | | | | 0.144 |
| Female | 145 (45.9) | 89 (43.6) | 56 (50.0) | |
| Male | 166 (52.5) | 115 (56.4) | 51 (45.5) | |
| Comorbidities | | | | |
| Anxiety and/or depression | 160 (50.6) | 99 (48.5) | 61 (54.5) | 0.313 |
| Chronic kidney disease | 3 (0.9) | 2 (1.0) | 1 (0.9) | 0.939 |
| Diabetes | 18 (5.7) | 12 (5.9) | 6 (5.4) | 0.847 |
| Dyslipidemia | 73 (23.1) | 53 (26.0) | 20 (17.9) | 0.101 |
| Other chronic comorbidity | 121 (38.3) | 81 (39.7) | 40 (35.7) | 0.485 |
| Risk factors | | | | |
| e-cigarette/vaping use in past 6 months | 26 (8.2) | 13 (6.4) | 13 (11.6) | 0.105 |
| Ever been on Medicaid | 67 (21.2) | 39 (19.1) | 28 (25.0) | 0.221 |
| Family history of heart disease or stroke | 113 (35.8) | 75 (36.8) | 38 (33.9) | 0.615 |
| Financial status: inadequate income | 248 (78.5) | 161 (78.9) | 87 (77.7) | 0.797 |

© 2023 Hoppe KK et al. *JAMA Network Open.*

Continuous variables are described by median [range] and tested with Mann-Whitney-Wilcoxon tests. Categorical variables are described by count (percent) and tested with chi-square tests, with the exception of education, number of HTN medications, and self-percieved health status which were ordered and tested with Mann-Whitney-Wilcoxon tests.

© 2023 Hoppe KK et al. *JAMA Network Open.*

eTable 2. ASA-24 Dietary Component Evaluation of Sodium, Combined Fruit and Vegetables, Whole Grain, and Saturated Fat Intake, Comparing Baseline With 6 and 12 Months

| **Dietary component evaluated** | **Time** | **Arm** | **N** | **Mean** | **Standard deviation** | **ANCOVA, P-value** |
|---|---|---|---|---|---|---|
| **Sodium (mg)** | Baseline | Control | 155 | 3673.81 | 1812.82 | ref |
| | | Intervention | 149 | 3512.82 | 1765.59 | |
| | 6-month | Control | 104 | 3968.20 | 1725.17 | **0.003** |
| | | Intervention | 99 | 3354.72 | 1365.75 | |
| | 12-month | Control | 90 | 4213.67 | 1972.37 | 0.138 |
| | | Intervention | 86 | 3682.94 | 1874.53 | |
| **Combined fruit and vegetables (cup equivalent)** | Baseline | Control | 155 | 2.50 | 1.98 | ref |
| | | Intervention | 149 | 2.34 | 2.02 | |
| | 6-month | Control | 104 | 2.57 | 1.89 | 0.639 |
| | | Intervention | 99 | 2.43 | 1.75 | |
| | 12-month | Control | 90 | 2.55 | 1.88 | 0.954 |
| | | Intervention | 86 | 2.50 | 1.92 | |
| **Whole grain (ounces)** | Baseline | Control | 155 | 0.85 | 1.41 | ref |
| | | Intervention | 149 | 0.61 | 1.05 | |
| | 6-month | Control | 104 | 1.12 | 1.62 | 0.278 |
| | | Intervention | 99 | 0.88 | 1.49 | |
| | 12-month | Control | 90 | 1.14 | 2.24 | 0.544 |
| | | Intervention | 86 | 0.82 | 1.25 | |
| **Saturated fat (%kcal)** | Baseline | Control | 155 | 12.54 | 4.32 | ref |
| | | Intervention | 149 | 11.91 | 4.19 | |
| | 6-month | Control | 104 | 13.12 | 6.56 | 0.979 |
| | | Intervention | 99 | 12.97 | 4.62 | |
| | 12-month | Control | 90 | 13.46 | 4.52 | 0.069 |
| | | Intervention | 86 | 12.03 | 4.72 | |

Continuous variables are described by mean (sd) and tested with t- tests.

© 2023 Hoppe KK et al. *JAMA Network Open.*

**eTable 3.** Sensitivity Analyses

| Measure | Godin 6mo | Godin 12mo | Sodium 6mo | Sodium 12mo | Alcohol 6mo | Alcohol 12mo |
|---|---|---|---|---|---|---|
| Clinic Systolic | -3.24 (-9.88 to 3.40), p = 0.340 | 0.35 (-7.18 to 7.88), p = 0.928 | -0.55 (-2.39 to 1.29), p = 0.558 | -0.43 (-2.70 to 1.85), p = 0.714 | -0.10 (-0.65 to 0.46), p = 0.734 | 0.07 (-0.54 to 0.68), p = 0.829 |
| Clinic Diastolic | -1.21 (-6.15 to 3.73), p = 0.632 | -0.56 (-6.65 to 5.52), p = 0.856 | 0.02 (-1.35 to 1.39), p = 0.979 | 0.15 (-1.68 to 1.98), p = 0.874 | -0.04 (-0.46 to 0.39), p = 0.871 | 0.06 (-0.44 to 0.55), p = 0.823 |
| AMBP Systolic | -5.10 (-10.47 to 0.28), p = 0.064 | -1.80 (-8.74 to 5.15), p = 0.613 | 0.61 (-0.90 to 2.12), p = 0.429 | -0.15 (-2.28 to 1.99), p = 0.891 | -0.40 (-0.85 to 0.05), p = 0.085 | 0.05 (-0.53 to 0.63), p = 0.868 |
| AMBP Diastolic | -4.21 (-8.06 to -0.35), p = 0.034 | -3.60 (-8.48 to 1.28), p = 0.150 | 0.62 (-0.46 to 1.70), p = 0.261 | 1.02 (-0.47 to 2.51), p = 0.183 | -0.26 (-0.59 to 0.07), p = 0.123 | 0.10 (-0.31 to 0.51), p = 0.643 |

Listed results include are the coefficients, (95% CI), and p-value. The way our models are set up & the nature of the coefficients we used, the F-test p-value is the same as the interaction coefficient's p-value.

The coefficients for sodium are per +1,000mg, alcohol is +1 drink/week so negative coefficients mean larger BP decrease with more sodium or more drinks. The Godin coefficient is the difference from active & inactive groups, so a negative coefficient means larger BP decrease in the inactive group.

© 2023 Hoppe KK et al. *JAMA Network Open.*
