# Supplementary material for: Effect of a Telephone Health Coaching Intervention on Hypertension Control in Young Adults: The MyHEART Randomized Clinical Trial
Source: JAMA Netw Open. 2023 Feb 3;6(2):e2255618. doi: 10.1001/jamanetworkopen.2022.55618 (PMC9898821; doi:10.1001/jamanetworkopen.2022.55618)
Supplement: Supplement 3. — Data Sharing Statement [file jamanetwopen-e2255618-s003.pdf]

Data Sharing Statement

Hoppe. Effect of a Telephone Health Coaching Intervention on Hypertension Control in Young Adults. *JAMA Netw Open*. Published February 03, 2023.
doi:10.1001/jamanetworkopen.2022.55618

Data
Data available: No

Additional Information
Explanation for why data not available: We are currently drafting other manuscripts with the data, it can be available upon request.
